# Phylogenetic comparative methods improve the selection of characters for generic delimitations in a hyperdiverse Neotropical orchid clade

**DOI:** 10.1038/s41598-019-51360-0

**Published:** 2019-10-22

**Authors:** Diego Bogarín, Oscar A. Pérez-Escobar, Adam P. Karremans, Melania Fernández, Jaco Kruizinga, Franco Pupulin, Erik Smets, Barbara Gravendeel

**Affiliations:** 1 Jardín Botánico Lankester, Universidad de Costa Rica, Cartago, P.O. Box 302-7050 Costa Rica; 2 Herbarium UCH, Universidad Autónoma de Chiriquí, David, Chiriquí Panama; 3 Naturalis Biodiversity Center, Endless Forms group, Leiden, The Netherlands; 4 Comparative Plant and Fungal Biology, Royal Botanic Gardens, Kew, Richmond, Surrey TW9 3AB UK; 5 Gothenburg Global Biodiversity Centre, Gothenburg, Sweden; 6 Department of Plant and Soil Science, Texas Tech University, Lubbock, TX 79409 USA; 7 Hortus botanicus, Leiden University, Leiden, The Netherlands; 8 Harvard University Herbaria, 22 Divinity Avenue, Cambridge, Massachusetts USA; 9 Marie Selby Botanical Gardens, 811 South Palm Avenue, Sarasota, Florida 34236 USA; 10 University of Applied Sciences Leiden, Faculty of Science and Technology, Leiden, The Netherlands; 11 Institute of Biology Leiden, Leiden University, Leiden, The Netherlands; 12 KU Leuven, Ecology, Evolution and Biodiversity Conservation, Leuven, Belgium

**Keywords:** Plant evolution, Biodiversity

## Abstract

Taxonomic delimitations are challenging because of the convergent and variable nature of phenotypic traits. This is evident in species-rich lineages, where the ancestral and derived states and their gains and losses are difficult to assess. Phylogenetic comparative methods help to evaluate the convergent evolution of a given morphological character, thus enabling the discovery of traits useful for classifications. In this study, we investigate the evolution of selected traits to test for their suitability for generic delimitations in the clade *Lepanthes*, one of the Neotropical species-richest groups. We evaluated every generic name proposed in the *Lepanthes* clade producing densely sampled phylogenies with Maximum Parsimony, Maximum Likelihood, and Bayesian approaches. Using Ancestral State Reconstructions, we then assessed 18 phenotypic characters that have been traditionally employed to diagnose genera. We propose the recognition of 14 genera based on solid morphological delimitations. Among the characters assessed, we identified 16 plesiomorphies, 12 homoplastic characters, and seven synapomorphies, the latter of which are reproductive features mostly related to the pollination by pseudocopulation and possibly correlated with rapid diversifications in *Lepanthes*. Furthermore, the ancestral states of some reproductive characters suggest that these traits are associated with pollination mechanisms alike promoting homoplasy. Our methodological approach enables the discovery of useful traits for generic delimitations in the *Lepanthes* clade and offers various other testable hypotheses on trait evolution for future research on Pleurothallidinae orchids because the phenotypic variation of some characters evaluated here also occurs in other diverse genera.

## Introduction

Taxonomic delimitation is essential to understand, document, and quantify biodiversity. This is particularly true for species, which are regarded as the fundamental units of biological systems. Species delimitations and their numerous corresponding concepts are still hotly debated, yet relatively little has been discussed regarding supra-specific taxon delimitations^1–3^. Among such higher taxonomic ranks, the genera are important because they inform about discernable trait patterns shared among related species^4^, and are widely used as biodiversity indicators of biogeographical areas^5^, and even biomes^6^. Generic delimitations are based on several criteria that are often informed by morphological, chemical or physiological traits, the principle of monophyly, internal support (bootstrap, posterior probabilities) statistical node support in phylogenies, and even clade size (i.e., species number). Among these, morphology is perhaps the most commonly invoked criterion to segregate or subsume species aggregates^4^, yet morphological characters are often variable and converge across the angiosperm tree of life^7^, thus rendering the selection of suitable morphological characters for generic delimitations difficult.

The orchid family includes about 25,000 species and *ca* 750 genera. Its generic classification is dynamic, with hundreds of genera having been subsumed and segregated during the last decade^8^. Among recalcitrant orchid clades with complicated generic delimitations are the Pleurothallidinae, the most species-rich subtribe in the Neotropics (5,200 species^9–11^). The high species diversity derived from recent and rapid diversifications and the exceptionally wide spectrum of morphological features have made the classification of this group challenging^12^. Previous cladistic and contemporary systematic studies were largely based on morphology^13,14^. Using these studies as a framework, Pridgeon^9^ proposed the first molecular phylogenetic classification of the subtribe based on nuclear and plastid regions of 185 selected taxa (3.5% of the species in Pleurothallidinae). This study laid the foundation for the classification followed in *Genera Orchidacearum*^15^ which divided the subtribe into nine main clades. In the past 10 years, several phylogenetic studies aimed to increase taxon sampling or add more markers to the previous phylogenetic reconstructions, supported or redefined most of the taxonomic and generic concepts proposed by Pridgeon^9^ and Luer^16^. These phylogenetic re-evaluations covered almost all clades in the subtribe^17–21^.

**Figure 1:**
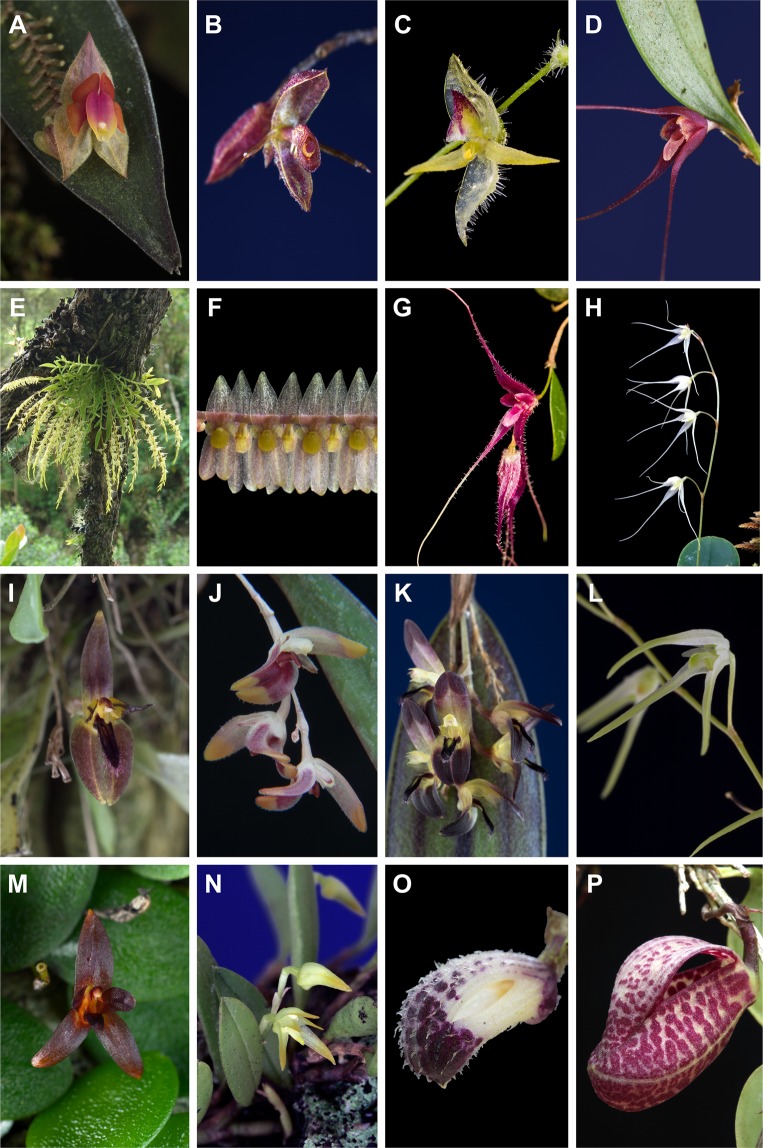
Flower morphology of the representatives of the *Lepanthes* clade: (**A**) *Lepanthes*; (**B**) *Draconanthes*; (**C**) *Pseudolepanthes*; (**D**) *Stellamaris*; (**E**) *Frondaria*; (**F**) *Lepanthopsis*; (**G**) *Gravendeelia*; (**H**) *Opilionanthe*; (**I**) *Lankesteriana*; (**J**) *Pendusalpinx*; (**K**) *Trichosalpinx*; (**L**) *Tubella*; (**M**) *Anathallis*; (**N**) *Anathallis*; (**O**) *Zootrophion*; (**P**) *Zootrophion* (*Epibator*). Photographs (**A**,**B**,**D**,**F**,**I**,**K–O**) by D.Bogarín, (**C,G**) by S. Vieira-Uribe, (**E**) by J. Portilla (Ecuagenera), (**H**,**J**,**P**) by W. Driessen.

One of the few remaining puzzling groups with poorly understood phylogenetic relationships in the Pleurothallidinae is the *Lepanthes* clade^9,11,22,23^ (Fig. 1). In its current circumscription, it comprises the genera *Anathallis* Barb.Rodr. (116 spp.), *Draconanthes* (Luer) Luer (two), *Epibator* Luer (three), *Frondaria* Luer (one), *Lankesteriana* Karremans (21), *Lepanthes* Sw. (>1,200), *Lepanthopsis* (Cogn.) Ames (44), *Trichosalpinx* Luer (24) and *Zootrophion* Luer (26). Moreover, four generic concepts needed to maintain monophyly, were recently erected by Bogarín *et al*.^23^: *Gravendeelia* Bogarín & Karremans (1), *Pendusalpinx* Karremans & Mel.Fernández (seven), *Stellamaris* Mel.Fernández & Bogarín (one), and *Opilionanthe* Karremans & Bogarín (one) as well as the reinstatement of *Pseudolepanthes* (Luer) Archila (10) and *Tubella* (Luer) Archila (79). The species largest genus is *Lepanthes*, which comprises more than 77% of the species of the clade.

**Figure 2:**
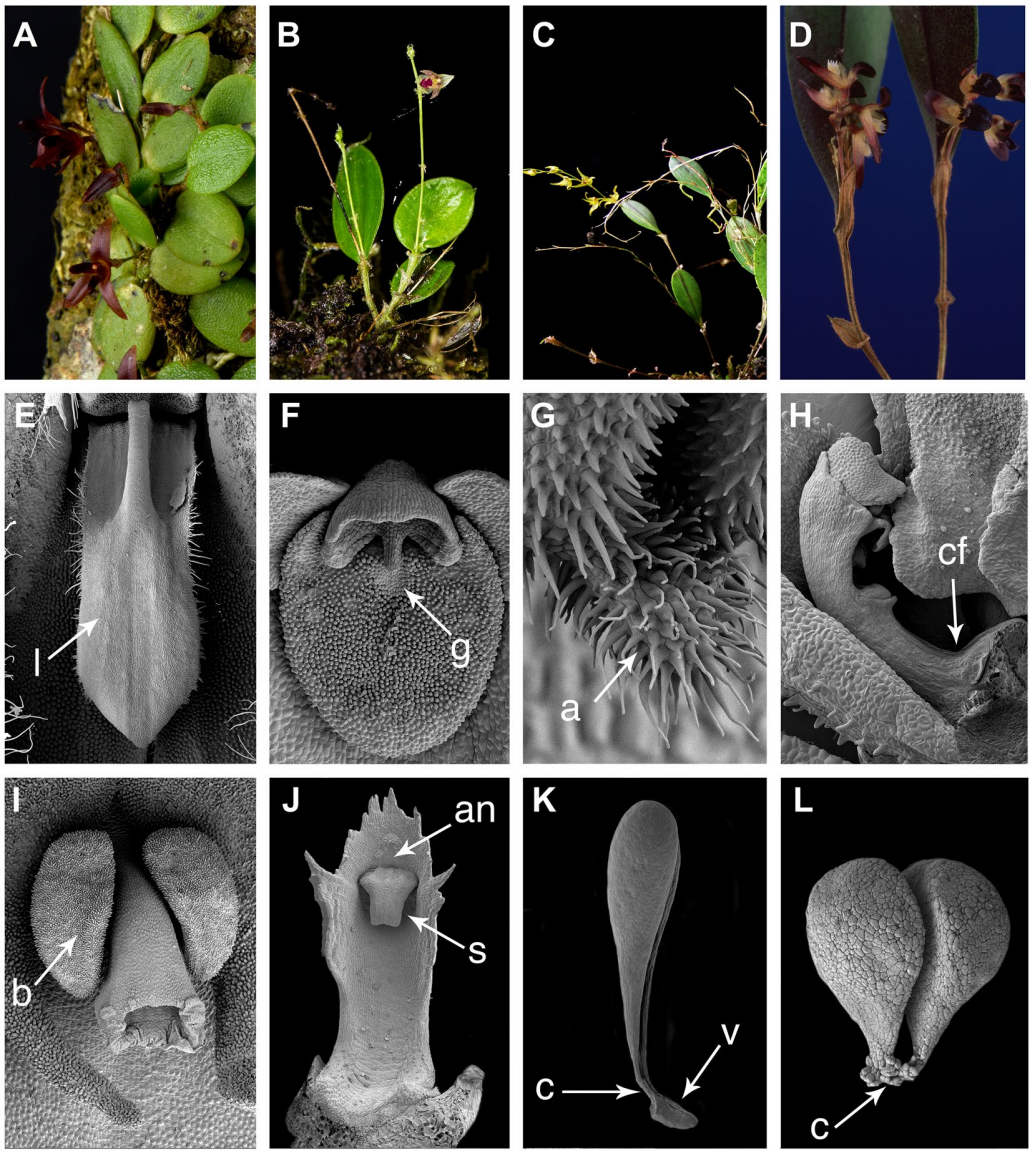
Vegetative and flower morphology of the characters evaluated: (**A**) creeping habit in *Anathallis*; (**B**) caespitose habit with longer inflorescences than leaf in *Pseudolepanthes*; (**C**) proliferating ramicauls in *Tubella*; (**D**) ornamented lepanthiform bracts in *Trichosalpinx*; (**E**) laminar, motile lip (**I**) of *Trichosalpinx*; (**F**) bilobed stigma and glenion (**G**) in *Lepanthopsis*; (**G**) Appendix (**A**) at the lip base of *Lepanthes*; (**H**) Column foot (cf) and ventral anther in *Gravendeelia*; (**I**) Bilobed lip (**B**) and apical anther in *Lepanthes*; (**J**) Ventral anther (an) and stigma (**S**) in *Anathallis*; (**K**) Pollinarium with viscidium (**V**) and caudicles (**C**) in *Lepanthe;* (**L**) Pollinarium with caudicles (**C**) in *Trichosalpinx*. Photographs (**A**–**I**) by D.Bogarín, (**B**) by S. Vieira-Uribe.

The *Lepanthes* clade is widely distributed in the Neotropics ranging from Mexico and Florida to southern Brazil and Argentina, including the Antilles. The species are characterized by infundibular sheaths along the ramicauls, also called “lepanthiform sheaths” of unknown functionality^9,24^. These sheaths are unornamented-papyraceous and imbricating in *Anathallis*, *Lankesteriana* and *Zootrophion*, foliaceous with expanded leaf sheaths in *Frondaria* and sclerotic with ornamentations (spiculate or muriculate) along the ramicauls in the remaining genera (Figs 1–2). Regardless of the relative uniformity in plant vegetative characters, flower morphology is highly dissimilar among genera, and no single diagnostic floral character distinguishing the group has been recognized. Floral trait variation is most evident in the flower shape (spread, flattened or cupped sepals and petals), color (red, yellow, white, green, purple or spotted), anthesis (simultaneous or successive), shape of sepals, petals and lip (elongate, flat, ciliate, bilobed), anther position (apical or ventral), pollinaria-associated structures (with or without viscidium), and presence/absence of a column foot, synsepal, and viscidium^13,15,22^ (Fig. 1).

Previous multi-locus analyses strongly supported the monophyly of the *Lepanthes* clade^8,15^, yet the number of genera to be recognized and their phylogenetic relationships are still unclear. This is likely due to the widespread homoplasy in reproductive characters in the clade and the insufficient phylogenetic taxon sampling. Earlier phylogenetic studies in the Pleurothallidinae did not investigate morphological evolutionary patterns, homoplasy and contrasting differences in reproductive traits by combining ancestral state reconstructions (ASR) and a solid phylogenetic framework^9,11^. This is essential to test hypotheses of morphological evolution and to disentangle recalcitrant generic delimitations due to phenotypic similarities. More importantly, theory predicts that synapomorphies or homoplastic characters are attributed to shifts or convergences due to dipteran pollination, but this remains yet to be tested due to the scarce pollination observations across the subtribe. The role of pollinator interactions in the evolution of the *Lepanthes* clade is currently unknown because only two pollination systems have been reported so far for *Lepanthes* and *Trichosalpinx*^25,26^.

Here, we explore the utility of molecular trees and phylogenetic comparative methods to discover suitable morphological characters for generic delimitation. To achieve this, we evaluate the relationships among members of the *Lepanthes* clade by assessing morphological characters within a phylogenetic framework. We performed ASRs on 18 floral morphological characters using a well-resolved phylogenetic inference from nuclear nrITS and plastid *matK* markers of 122 species covering all recognized genera within the clade^23^. We want to answer the following questions: (1) which monophyletic genera can be recognized based on a phylogenetic framework? (2) what are the phylogenetically informative characters of each clade based on ASRs? (3) how did such diagnostic morphological characters evolve in the clade? We also provide a detailed generic circumscription of *Lepanthes*.

## Results

Matrix statistics of the 148 accessions from the 120 species (including two outgroup accessions) and parsimony information for nrITS, *mat*K and concatenated datasets are summarized in Appendices S1,S2.

### Gene trees

The inferences of the BI, ML and MP from the nrITS dataset yielded similar topologies and high support for the 14 genera recognized as members of the *Lepanthes* clade but with some differences in the topology among the relationships of those clades (Appendices S3, S4). Some differences were observed in the placement of *Anathallis*, *Lankesteriana*, *Pendusalpinx*, *Trichosalpinx* and *Tubella* and in the position of *L*. *obliquipetala*, which was sister to *Lepanthopsis* + *Gravendeelia*. The relationships among *Lepanthes*, *Draconanthes*, *Pseudolepanthes*, and *Stellamaris* were consistent. In contrast, the inferences from the *matK* dataset showed several polytomies and low support values for most of the clades (Appendices S4, S5).

### Incongruence between nuclear and plastid datasets

A total of 24 terminals were detected as incongruent with ML and 34 with BI. Of those, 20 terminals were retrieved as incongruent by both inferences (Appendices S1, S6). The topology of the BI, MP and ML trees inferred from the concatenated datasets excluding/including the plastid conflicting sequences recognized essentially the same generic clades but showed some differences in the topology and support for intergeneric relationships (Appendices S1, S6, S7).

### Concatenated approach (nrITS + *mat*K)

**Figure 3:**
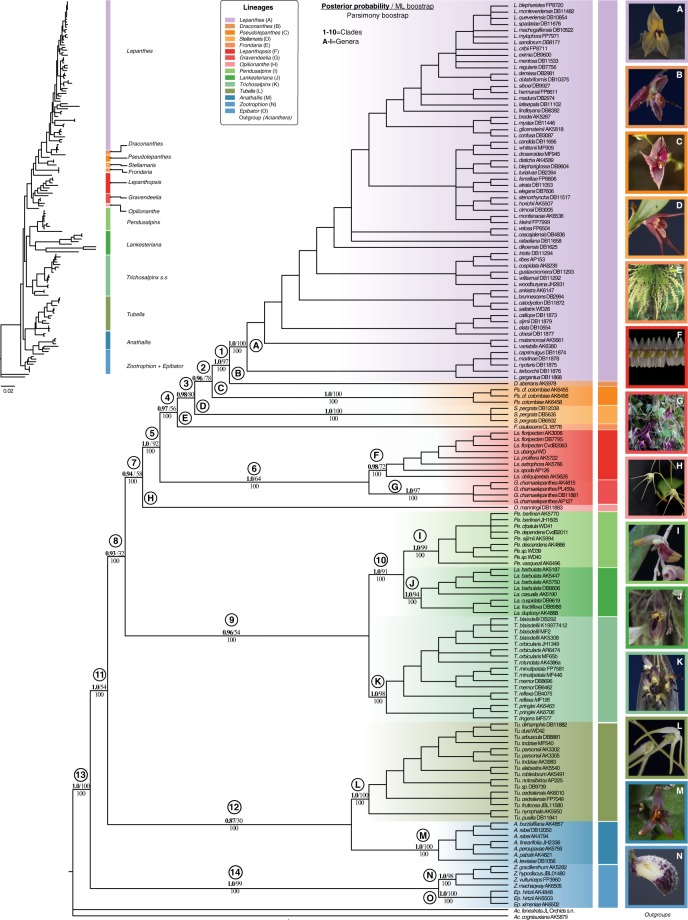
The 14 genera recognized in the *Lepanthes* clade in the 50% majority-rule consensus tree based on BI analysis of concatenated dataset. Labels of the genera follow the revised generic names as outlined in the discussion. Plotted branch values for PBP, LPB and PP are given for each well-supported clade of interest. Letters represent genera and numbers clades grouping the genera. Photographs (**A**,**B**,**D**,**F**–**G**,**J**–**N**) by. D. Bogarín, (**C**) by S. Vieira-Uribe, (**E**) by J. Portilla (Ecuagenera), (**H**–**I**) by W. Driessen.

Consistent with the inferences based on nrITS, the BI, ML and MP analyses from the concatenated dataset retrieved in the same generic groupings with high support values for all the genera of the *Lepanthes* clade (Fig. 2 and Appendix S1). The support slightly increased after removing the potential outliers from the plastid dataset. In contrast, despite the consistent topologies and high support obtained for all genera, the relationships among them differed using the original datasets (as well as the nrITS dataset alone). However, these relationships received higher support in the analyses after removing the detected potential outliers from the *matK* dataset and the phylogenetic relationships obtained were topologically most similar among BI, ML and MP (Fig. 3, Appendices S6,S7). Also, we show support for inferences with/without PACo in Appendix S6. Consistent with the high support obtained with BI, the inferred network did not show phylogenetic uncertainty.

### Phylogenetic relationships and generic clades

**Figure 4:**
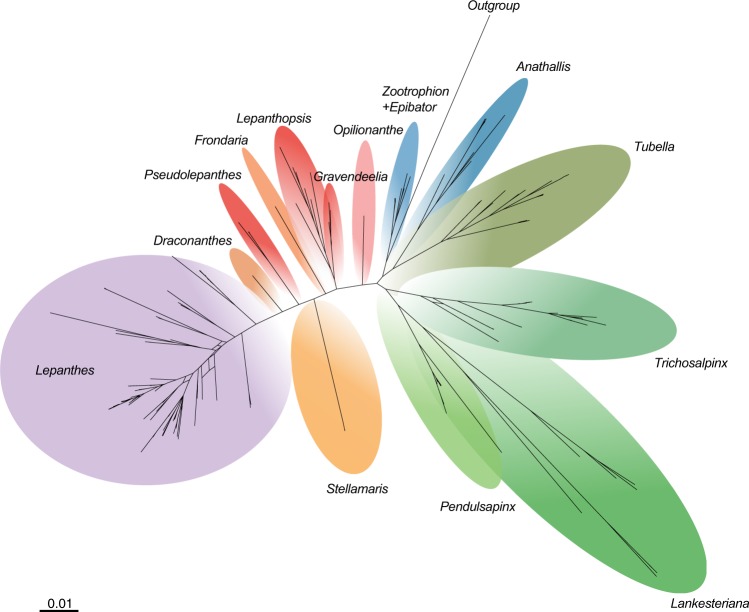
Split network showing the 14 genera of the *Lepanthes* clade inferred from 3,000 tree replicates of the BI inference. The network shows well supported groups without uncertainty in the relationships.

We obtained strong support for recognizing 14 subclades within the *Lepanthes* clade (Figs 3,4). *Lepanthes* (clade A) was supported as monophyletic in all the analyses (PBP = 100, LPB = 100 and PP = 1.0) and sister to *Draconanthes* (clade B). The clustering of *Lepanthes* + *Draconanthes* was well supported in all the analysis: parsimony bootstrap percentage = 100 (PBP), LPB maximum likelihood boostrap percentage = 100 (LPB) and, Bayesian posterior probability = 1.0 (PP). The accessions of *Pseudolepanthes* (clade C) grouped with high support (PBP = 100, LPB = 100, and PP = 1.0) and this genus was sister to *Lepanthes* + *Draconanthes* (clade 1). The accessions of *Stellamaris pergrata* (Ames) Mel.Fernández & Bogarín (clade D) were well supported and the group was sister to *Lepanthes* + *Draconanthes* + *Pseudolepanthes* (clade 2) (LPB = 80% and PP = 0.98). When phylogenetic incongruence was not considered, *Pseudolepanthes* and *Stellamaris* clustered in a clade with strong support in the MP tree (PBP = 100). The genus *Frondaria* (clade E) was found to be related to *Lepanthes*, *Draconanthes*, *Pseudolepanthes*, *Stellamaris* (clade 3), well supported (PBP = 100 and PP = 0.97) but lacking support in the ML analysis (LPB = 56%). Clade 4 made up by clade 3 + *Frondaria* and comprised the species more related to the core of *Lepanthes* whereas *Lepanthopsis* (clade F) and *Gravendeelia* (clade G) both clustered in clade 6. The clades 1–4 and 6 are clustered (clade 5) with high support. Most nodes of these clades were well supported (PBP > 100, LPB > 72 and PP > 0.98) with the only exception being clade 6 with low LPB support but well supported by PBP > 100 and PP > 0.98. The genus *Opilionanthe* was sister to clade 5 + clade 6 with high support for PBP = 100, unsupported by BI (PP = 0.94) and low support for ML (LPB = 58). Topologically, *Opilionanthe* always clustered apart from the other generic clades discussed here. Related to the groups of clade 7 (members of the core of *Lepanthes* and *Lepanthopsis*) was a group consisting of species related to *Trichosalpinx* s.s. (clade K), *Pendusalpinx* (clade J) and *Lankesteriana* (clade I) all highly supported (PBP = 100, LPB ≥94 and PP = 1.0). This topology was retrieved with high to moderate support (PBP = 100, LPB ≥54 and, PP ≥ 0.96) after removing incongruences using the Procrustean Approach to Cophylogeny (PACo) application^27^. *Tubella* (clade L) and *Anathallis* (clade M) were highly supported (PBP = 100%, LPB = 100 and PP = 1.0). The internal relationships of clade 12 received low support with ML (PBP ≤ 30) and BI (PP ≤ 0.87) but high support by MP (PBP = 100%). Clade 14, comprising *Zootrophion* (clade N) and *Epibator* (clade O), was well supported in all the analyses (PBP = 100, LPB ≥98 and PP = 1.0). The most constant well-supported relationships among all the analyses were the clustering of *Zootrophion* (PBP = 100%, LPB ≥99, PP = 1.0), *Lankesteriana* and *Pendusalpinx* (PBP = 100, LPB ≥91, PP = 1.0), *Lepanthes* + *Draconanthes* (PBP = 100, LPB ≥88%, PP = 1.0) and the clustering of the genera related to the core of *Lepanthes* (clade 4) with *Lepanthopsis* + *Gravendeelia* (clade 6) (PBP = 100, LPB ≥8 and, PP = 1.0).

### Character evolution

**Figure 5:**
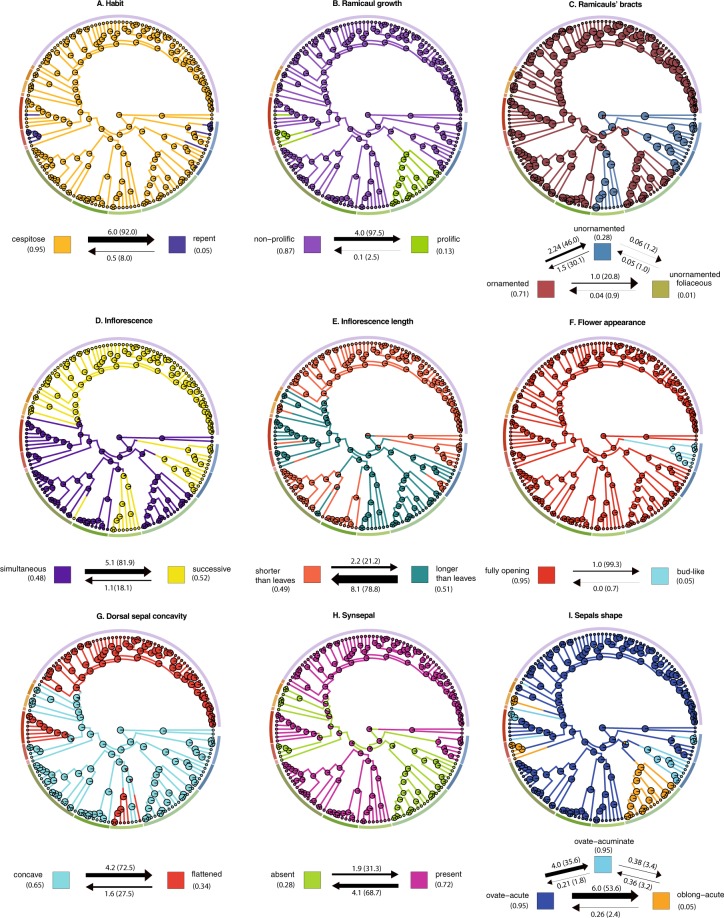
Ancestral state reconstructions of selected morphological characters from stochastic mapping analyses based on joint sampling (10,000 mapped trees). Arrows represent transitions between states and numbers represent the estimated number of evolutionary changes with proportion in parenthesis and the time spent in each state. Posterior probabilities (pie charts) are mapped in a random stochastic character map. External subdivided ring represents the 14 recognized genera. (**A**) Habit; (**B**) Ramicauls growth; (**C**) Bracts of ramicauls; (**D**) Inflorescence; (**E**) Inflorescence length; (**F**) Flower appearance; (**G**) Dorsal sepal concavity; (**H**) Synsepal. (**I**) Sepal shape.

**Figure 6:**
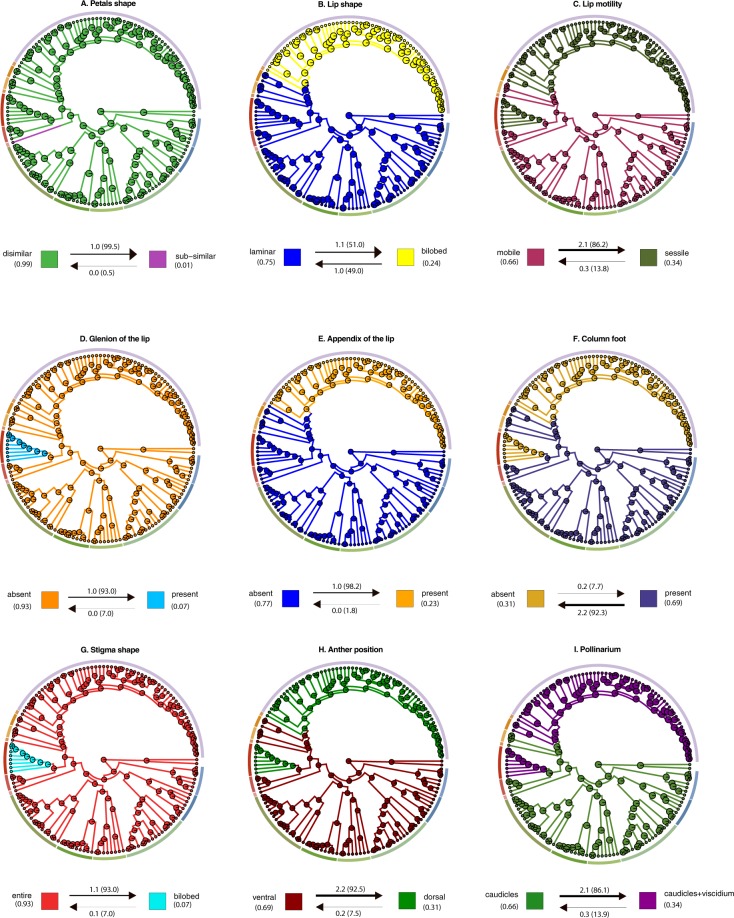
Ancestral state reconstructions of selected morphological characters: (**A**) Petals shape; (**B**) Lip shape; (**C**) Lip motility; (**D**) Glenion of the lip; (**E**) Appendix of the lip; (**F**) Column foot; (**G**) Stigma shape; (**H**) Anther position; (**I**) Pollinarium.

ASR was based on the one-rate model ER that was consistently better than the SYM and ARD models (Appendices S8, S10). These estimations were obtained using phylograms from MrBayes and ultrametric trees from BEAST calculated under the Birth Death as the best speciation model according to a Bayes factors test (Appendix S11). Estimations based on the reversible-jump Markov Chain Monte Carlo (MCMC) model yielded similar results compared to the rates obtained with SCM (Appendices S12, S14). For the MCMC approach with BayesTraits V3 the best results were obtained with the hyperprior adjusted to the previously obtained ML transition rates (from 0 to 0.03). The ACE, SIMMAP and re-rooting methods yielded identical scaled-likelihoods at the root state and the estimations with MCMC revealed essentially the same results obtained with ACE and SIMMAP with ambiguous estimations for the characters of inflorescence length and synsepal (Table 1). Characters states of the common ancestor suggest that plesiomorphic features are a caespitose habit with non-proliferating, unornamented-papyraceous ramicauls, simultaneously flowering inflorescences, fully opening flowers with concave, ovate-acute dorsal sepals, dissimilar petals, column with a foot, a laminar, motile lip without glenion and a ventral anther with entire stigma (Table 2). The most common character state transitions are: a caespitose to creeping/pendent habit, ornamented to unornamented-papyraceous bracts, non-proliferating to proliferating ramicauls, simultaneously flowering to successively flowering inflorescences, shortening of inflorescences, fully opening flowers to bud-like flowers, ovate-acute to ovate-acuminate/oblong-acute sepals, concave to flattened dorsal sepals, dissimilar to subsimilar petals, loss of a column foot and synsepal, movable to sessile lip, entire to bilobed stigma, ventral to dorsal anther and pollinarium with naked caudicles to caudicles with a viscidium (Figs 5, 6). Probabilities favoring reversal transitions from proliferating to non-proliferating ramicauls, foliaceous to ornamented/unornamented-papyraceous bracts, creeping to caespitose habit, bud-like to opening flowers, subsimilar to dissimilar petals, oblong-acute to ovate-acuminate/ovate-acute sepals, presence of a glenion to absence, sessile to motile lip, absence of a column foot to presence, dorsal to apical anther, bilobed to entire stigma and pollinarium with caudicles and a viscidium to lack of a viscidium, were found to be unlikely. Lip shape from laminar to bilobed and vice-versa showed a similar probability (Figs 5, 6). Twelve homoplastic characters and seven synapomorphic characters were detected (Table 2). The combination of a sessile lip, absence of a column foot, dorsal anther and pollinarium with caudicles and viscidium are features only observed in *Lepanthes*, *Draconanthes*, *Pseudolepanthes* and *Lepanthopsis*, whereas motile lips, a column foot, ventral anther and pollinarium with caudicles are observed in all other genera investigated.

**Table 1:** Marginal probability of the root state as estimated with ACE, SCM (ER model) and Bayesian Inference.

Characters	ML(ACE)			SCM (SIMMAP)			BI (RevJump)		
	state 0	state 1	state 2	state 0	state 1	state 2	state 0	state 1	state 2
Habit: (0) caespitose; (1) creeping	0.99	0.01	—	0.99	0.01	—	0.99	0.01	—
Ramicaul growth: (0) non-proliferating; (1) proliferating	1.00	0.00	—	1.00	0.00	—	1.00	0.00	—
Bracts of ramicauls: (0) unornamented-papyraceous; (1) ornamented; (2) unornamented-foliaceous	0.78	0.21	0	0.82	0.18	0.00	0.73	0.19	0.08
Inflorescence: (0) simultaneously flowering; (1) successive flowering	0.95	0.05	—	0.97	0.03	—	0.98	0.02	—
Inflorescence length: (0) shorter; (1) longer (than leaves)	0.43	0.57	—	0.46	0.54	—	0.07	0.93	—
Flower appearance: (0) fully opening; (1) bud-like	1.00	0.00	—	1.00	0.00	—	1.00	0.00	—
Dorsal sepal concavity: (0) concave; (1) flattened	1.00	0.00	—	1.00	0.00	—	1.00	0.00	—
Synsepal: (0) absent; (1) present	0.07	0.93	—	0.06	0.94	—	0.47	0.53	—
Sepals shape: (0) oblong-acute; (1) ovate-acuminate (2) ovate-acute	0.01	0.01	0.98	0.01	0.01	0.98	0.29	0.08	0.63
Petals shape: (0) dissimilar; (1) subsimilar	1.00	0.00	—	1.00	0.00	—	1.00	0.00	—
Lip shape: (0) laminar; (1) bilobed	1.00	0.00	—	1.00	0.00	—	1.00	0.00	—
Lip motility: (0) motile; (1) sessile	1.00	0.00	—	1.00	0.00	—	1.00	0.00	—
Glenion of the lip: (0) absent; (1) present	1.00	0.00	—	1.00	0.00	—	1.00	0.06	—
Appendix of the lip: (0) absent; (1) present	1.00	0.00	—	1.00	0.00	—	1.00	0.00	—
Column foot: (0) absent; (1) present	0.00	1.00	—	0.00	1.00	—	0.00	1.00	—
Stigma shape: (0) entire; (1) bilobed	1.00	0.00	—	1.00	0.00	—	1.00	0.00	—
Anther position: (0) ventral; (1) dorsal	1.00	0.00	—	1.00	0.00	—	1.00	0.00	—
Pollinarium: (0) with caudicles; (1) with caudicles + viscidium	1.00	0.00	—	1.00	0.00	—	1.00	0.00	—

**Table 2:** Cladistic classification of the 18 morphological characters assessed.

Characters	Plesiomorphy	Synapomorphy	Homoplasy
Habit	caespitose	—	creeping
Ramicauls	non-proliferating	—	proliferating
Ramicauls’ bracts	unornamented-papyraceous	unornamented-foliaceous (*Frondaria*)	ornamented
Inflorescence	simultaneous	—	successively flowering
Inflorescence length	*	—	shorter/longer than leaves
Flower appearance	fully opening	bud-like (*Zootrophion*)	—
Dorsal sepal concavity	concave	—	flattened
Synsepal	*	—	absent/present
Sepal shape	ovate-acute	—	oblong-acute/ovate-acuminate
Petals shape	dissimilar	subsimilar (Opilionanthe)	—
Lip shape	laminar	bilobed (*Lepanthes*)	—
Lip motility	motile	—	sessile
Glenion of the lip	absent	present (*Lepanthopsis*)	—
Appendix of the lip	absent	present (*Lepanthes*)	—
Column foot	present	—	absent
Stigma shape	entire	bilobed (*Lepanthopsis*)	—
Anther position	ventral	—	dorsal
Pollinarium	with caudicles	—	caudicles + viscidium

Plesiomorphic characters detected with marginal probability at the root state (Table 1) * = ambiguous character at the root state. Synapomorphic and homoplastic characters based on SCM calculations.

## Discussion

### Phylogenetics of the *Lepanthes* clade

In this section, we discuss the nomenclatural changes needed to redefine the *Lepanthes* clade as proposed by Bogarín *et al*.^23^ as well as the relationships among these genera based on the phylogenetic insights and morphological evolution of key characters as presented in this study. The *Lepanthes* clade comprises four main subclades (clades 7, 9, 12 and 14 as shown in Fig. 3). *Zootrophion* is plus *Epibator*, sister to the rest of the subclades. The next successively diverging clade is *Anathallis*, plus *Tubella* (see further discussion on *Trichosalpinx* s.l). *Anathallis* was initially re-established for the species of *Pleurothallis* subgenus *Acuminatia* sect. *Alata* and *Pleurothallis* subgenus *Specklinia* sect. *Muscosae*^28^. The clade composed of *Anathallis* plus *Panmorphia* Luer is confirmed monophyletic, and the exclusion of members of *Pleurothallis* subgenus *Acuminatia* sect. *Acuminatae*, which are related to *Stelis* s.l.^17^. Also, Karremans^29^ established the genus *Lankesteriana* because its members were closely related to *Pendusalpinx* rather than to *Anathalllis* s.s. as suggested by Pridgeon *et al*.^9^. *Trichosalpinx* as previously circumscribed^15,30^ is confirmed as polyphyletic, and therefore re-circumscribed. The species belonging to *Pendusalpinx* and *Tubella* are confirmed to be unrelated to *Trichosalpinx* and therefore excluded, whereas *Gravendeelia*, *Opilionanthe*, *Pseudolepanthes* and *Stellamaris* are placed for the first time in a phylogenetic framework and recognized as distinct^23^. The polyphyly of *Trichosalpinx* was suggested in previous studies but the newly proposed genera were not evaluated or the sampling was too incomplete to allow a redefinition of these groups^11^. The relationships recovered here also suggest that only *Pendusalpinx* and *Lankesteriana* are closely related to *Trichosalpinx* s.s. As suggested by Pridgeon^9^, members of *Tubella* are isolated from *Trichosalpinx* and *Pendusalpinx* but these relationships were not supported^12^. Here, with the inclusion of members of the clade not previously evaluated (*Gravendeelia*, *Opilionanthe*, *Pseudolepanthes*, and *Stellamaris*), *Tubella* is now sister to *Anathallis*.

The most recently diverging clade of the *Lepanthes* clade consists of *Lepanthes* and its allied genera: *Draconanthes*, *Gravendeelia*, *Lepanthopsis*, *Opilionanthe*, *Pseudolepanthes* and *Stellamaris*. With the exception of *Draconanthes* and *Lepanthopsis*, these genera were formerly all treated under *Trichosalpinx* s.l. However, we confirm here that they are closely related to *Lepanthes* and *Lepanthopsis* rather than to *Trichosalpinx* s.s. In addition, a clade composed of *Lepanthopsis* plus *Expedicula* was found monophyletic. In the next sections, we discuss the morphological characters supporting the new classification for the *Lepanthes* clade proposed here.

### Morphological evolution

Our character reconstructions improved the understanding of the evolution of phenotypic traits used to classify the genera of the *Lepanthes* clade. We identified homoplastic characters, that are not suitable for generic circumscriptions, as well as synapomorphies (Table 2). Plant habit (caespitose or creeping) evolved several times with a higher transition frequency from caespitose to creeping. This was found for other groups within Pleurothallidinae as well, possibly as an adaptation to different environments. Proliferating ramicauls evolved from non-proliferating ones independently in four clades. The lack of ornamentation of the ramicauls confused taxonomists as the close relationship of *Zootrophion*, *Anathallis*, and *Lankesteriana* with *Lepanthes*, *Lepanthopsis* and *Trichosalpinx* s.l. was not recognized previously. In addition, a combination of plesiomorphic and homoplastic characters in *Trichosalpinx* s.l., such as the ornamentation of the ramicauls, concave dorsal sepals, ovate-acuminate, caudate petals, motile, laminar lips with a column foot and ventral anthers caused misclassifications of the now separated genera *Gravendeelia*, *Pendusalpinx*, *Opilionanthe*, and *Stellamaris*. Assessment of other potential diagnostic traits was needed for these genera to complement a classification based solely on homoplastic characters. For example, the synapomorphic sub-similar petals in *Opilionanthe* are a diagnostic feature of the genus, showing a low probability of transition back to the ancestral state, dissimilar petals.

Inflorescence type and length are also variable characters in Pleurothallidinae^13^. Although groups show trends towards the presence of only one of the states, there are always exceptions. For example, all species of *Lepanthes* studied here have inflorescences shorter than the leaves but some species (not sampled in this study) have inflorescences longer than the leaf. The opposite is observed in *Trichosalpinx*^30^. The ancestral traits recovered for the anther position, column foot, pollinarium type, and lip motility suggest that these are associated with pollinators that enter the flower using the laminar lip. When ﻿trying to depart the flower, the dorsal part of the insect scrapes the anther of the column in the area of the caudicles and removes the pollinarium^26,31–33^. This mechanism predominates in *Zootrophion*, *Tubella*, *Anathallis*, *Trichosalpinx*, *Lankesteriana*, *Pendusalpinx*, *Opilionanthe*, *Gravendeelia*, *Frondaria* and *Stellamaris*. The recent discovery of biting midges in *Forcipomyia* (Ceratopogonidae) as pollinators of two species of *Trichosalpinx* highlights the importance of the motile, papillose, ciliate lip for their pollination^26^. Additional floral micromorphological characters of these three genera, such as the papillose surface of the lip with striated cuticles and secretions of proteins as possible rewards support a hypothesis of floral convergence^26^. The flowers of some species of *Anathallis*, *Tubella* and *Opilionanthe* are similar to other pleurothallids, such as the white-flowered *Specklinia calyptrostele*, visited by biting midges (Ceratopogonidae)^21^, suggesting that floral similarities are prone to convergent pollination.

The predominance of an ancestral morphology adapted to pollination by biting midges makes these characters unsuitable for generic classification. The combination of a sessile lip, without a column foot, dorsal anther and pollinarium with caudicles and viscidium is only observed in *Lepanthes*, *Draconanthes*, *Pseudolepanthes* and *Lepanthopsis*, whereas motile lips, a column foot, ventral anther and pollinarium with caudicles are observed in all other genera^24,30^. Synapomorphic characters of *Lepanthes*, such as an appendix, in combination with a viscidium and a sessile lip are key features for pollination by sexual deception^25^. Even though pollination observations are documented only for a handful of species of this genus, the floral synapomorphies indicate that pseudocopulation is likely to be predominant in the group. *Lepanthes*-like flowers are also found in species of the former *Lepanthes* subgenus *Brachycladium* Luer, today known to belong to the distantly related *Andinia*^34^. Floral convergence is probably due to selective pressure as suggested by Wilson *et al*.^34^ based on pollination observations by Álvarez^35^.

In *Lepanthopsis*, autapomorphic characters such as a glenion and bilobed stigma suggest an adaptation to different, yet unknown pollinators as compared to *Lepanthes* and *Trichosalpinx*^25,26^. *Lepanthopsis* and *Gravendeelia* are grouped in the same clade and the need for recognition of *Gravendeelia* is supported by autapomorphic characters of *Lepanthopsis* such as the glenion and bilobed stigma. As transitions of these characters to the ancestral state are unlikely, it seems that floral evolution in *Lepanthopsis* and *Gravendeelia* took a different path. Floral morphology of *Lepanthopsis* resembles that of *Platystele* Schltr. and the autapomorphic characters such as the presence of a glenion and bilobed stigma suggest an adaptation to different, yet unknown pollinators. In contrast, *Gravendeelia* has a floral morphology oriented towards pollination that likely involves a similar behavior of insects as described in *Trichosalpinx* s.s^26^.

Ambiguous results obtained for inflorescence length, the formation of a synsepal at the root state and, the higher frequency of transitions between different states indicates that these traits evolved independently in several groups within Pleurothallidinae^15^. The synsepal is made up of fused lateral sepals, and this condition varies from unfused to fully fused. A possible correlation between sexual mimicry and successive flowering in *Lepanthes* suggests that all flowers opening at the same time might not be an optimal strategy to fool male fungus gnats (Sciaridae), because several female-mimicking flowers together may accelerate males not being tricked^36,37^. In contrast, the meagre rewards for female biting midges in *Trichosalpinx* flowers suggest that several flowers opening at the same time might be more advantageous for attracting pollinators^26^.

### Circumscription of the genera in the *Lepanthes* clade

*Lepanthes* has been consistently supported as comprising a clade in previous studies^9,12,23^ (Fig. 3, Appendix S15). Species of the genus are known for their caespitose habit with lepanthiform sheaths. Among its close relatives, the transversely bilobed petals, bilobed lip with a basal appendix, elongate column with apical anther, and viscidium are diagnostic for our favored circumscription of the genus. Several earlier proposed subgeneric divisions of *Lepanthes*^38^ were not supported by our molecular phylogenetic analyses and will require re-evaluation whenever a broader sampling becomes available. *Draconanthes* was based on the former *Lepanthes* subgenus *Draconanthes*^38^, currently made up of two species known only from high elevations. It is sister to *Lepanthes s*.*s*., *Draconanthes* and *Lepanthes* are morphologically similar but the former may be distinguished by the rigid sepals, linear elongate, unlobed petals and a fleshy lip with a rudimentary appendix-like structure in contrast with the elaborate appendixes of *Lepanthes*. As we did not evaluate the evolution of these particular traits, an alternative option is to treat *Draconanthes* under *Lepanthes* based on similarities instead of the differences here discussed. *Pseudolepanthes* is sister to *Lepanthes* plus *Draconanthes*, rather than being related to *Trichosalpinx* as was previously assumed^9^. *Pseudolepanthes* resembles species of *Lepanthes* in plant architecture, but its species are immediately set aside by the spreading, linear to narrowly ovate petals, and the laminar, appendix-free lip with a prominent warty callus, which suggest a different pollination strategy as compared to pseudocopulation recorded in *Lepanthes*^30^. *Stellamaris* currently includes a single species, *Stellamaris pergrata*, previously believed to belong to *Trichosalpinx*^39^. It is sister to *Lepanthes* plus *Draconanthes* and *Pseudolepanthes* instead. With the last, it shares the caespitose habit, but it can be distinguished by a short, few-flowered inflorescence, long-caudate sepals, a callose lip, an elongate column with an incumbent anther and a prominent column foot, and no viscidium^23,30^. *Frondaria* can be distinguished by the synapomorphic conspicuous foliaceous sheaths along the stems. Contrary to the terminal leaf, the smaller leafy bracts do not have an abscission layer which is consistent with them being overgrown, green bracts rather than true leaves. *Frondaria* produces elongate inflorescences with simultaneously opening, white flowers with spreading, acuminate sepals that are virtually indistinguishable from those of the distantly related genera *Anathallis* and *Tubella*. *Lepanthopsis* plus *Gravendeelia* are is sister to *Lepanthes*, *Draconanthes*, *Pseudolepanthes*, *Stellamaris* and *Frondaria*. Species of the genus are recognized by the inflorescences with simultaneously opening, flattened flowers, provided with a fleshy, simple lip with a glenion at the base and a short column with a bilobed stigma^40^. A few exceptions to this scheme are found in *Lepanthopsis* subgen. *Microlepanthes* Luer^40^. *Gravendeelia* is monotypic and sister to *Lepanthopsis*. *Gravendeelia chamaelepanthes* (Rchb.f.) Bogarín & Karremans, undoubtedly represents a species complex in need of further revision. It differs from *Lepanthopsis* in the chain-like, pendent habit, the few-flowered inflorescences with tubular flowers with elongate sepals, an elongate lip without a glenion and the elongate column with a distinct foot and unlobed stigma^23,30^. Both plants and flowers of *Gravendeelia* are different from *Lepanthopsis* that their close phylogenetic relationship is one of the most unexpected results of this study (Fig. 3, Appendix S15). The flowers resemble those of the unrelated genera *Anathallis*, *Stellamaris* and *Tubella*. *Opilionanthe*, formerly placed in *Trichosalpinx*, is sister to *Lepanthes*, *Draconanthes*, *Pseudolepanthes*, *Stellamaris*, *Frondaria*, *Lepanthopsis* and *Gravendeelia*. The lepanthiform bracts, caespitose habit and more or less tubular white flowers are reminiscent of *Tubella*, thus the isolated phylogenetic placement of this species was unexpected. However, *O*. *manningii* (Luer) Karremans & Bogarín is immediately distinguished from species belonging to other genera by the sub-orbicular leaves and the long-caudate petals, which are subsimilar to the sepals^23^.

*Lankesteriana*, *Pendusalpinx*, and *Trichosalpinx* are florally similar as they share purplish flowers with a motile, ciliate lip, attached to a column foot, and an ventral anther and stigma^23,29,30^. The vegetative morphology, however, is distinct. Species of *Lankesteriana* can be easily distinguished from *Trichosalpinx* and *Pendusalpinx* by the extremely small habit with ramicauls that lack ornamented lepanthiform bracts shorter than the leaves and the successively flowering inflorescences^29^.

*Trichosalpinx* and *Pendusalpinx* are vegetatively similar to each other, with a large size long ramicauls and simultaneously flowered inflorescences. *Pendusalpinx* differs in its pendent habit with large, whitish lepanthiform bracts and glaucous leaves^23^. Based on vegetative morphology alone it is unexpected that *Lankesteriana* and *Pendusalpinx* should be sister to each other. However, these findings are congruent with those of previous studies^20,29^. On the other hand, contrary to what was found by those authors^20,29^, *Lankesteriana* and *Pendusalpinx* are here found to be sister to *Trichosalpinx* as previously supported^12^. Due to the contradictory inferences, the relatively long branches of the *Lankesteriana* accessions, and the highly diverging morphologies, we remain cautious as to the stable phylogenetic relationships between these three genera. It is possible that the similar floral morphology was caused by convergent evolution due to a similar pollination strategy rather than common ancestry^23^. An alternative hypothesis is to treat these genera under *Trichosalpinx* based on the high support obtained and floral similarities discussed above (Appendix S15).

*Anathallis* and *Tubella* are each well supported but moderately to weakly supported as sister genera in the BI and ML analyses, and therefore their relationship remains weakly supported. *Anathallis* is distinguished by the non-lepanthiform sheaths, caespitose ramicauls, and the free, star-shaped perianth^16,29^. Some species have purple flowers with motile lips, whereas others share similar micromorphological characters with *Lankesteriana*, *Pendusalpinx* and *Trichosalpinx* s.s. such as the striated cuticles and secretion of proteins^26^. Members of *Pleurothallis* subgenus *Acuminatia* sect. *Acuminatia* are phylogenetically related to *Stelis* s.l. and should therefore not be considered as part of *Anathallis*^17^. Allied to *Lepanthes*, *Lepanthopsis*, *Trichosalpinx* and their allies (clade 8) are members of *Tubella*, a group previously recognized as a subgenus of *Trichosalpinx*^30,39^. It comprises mostly slender plants with creeping ramicauls, simultaneously flowering inflorescences with whitish flowers and elongate sepals.

*Zootrophion* plus *Epibator* are placed as sister to all other members of the *Lepanthes* clade. They can be distinguished by the partial opening of the flowers due to the apical fusion on the sepals. As a consequence, the flowers have a single opening on each side, giving them a unique appearance. This feature, present in all species of *Zootrophion*, is not present in the other members of the *Lepanthes* clade; however, it is present in other unrelated genera of the Pleurothallidinae. The synsepal is thick and verrucose, and the lip is minute. The bracts are large, unornamented-papyraceous and loose.

## Conclusions

Generic delimitations of orchids based on morphological traits is made challenging daunting because of extensive homoplasy in characters previously used for circumscriptions. The *Lepanthes* clade has long challenged systematists and taxonomists due to the floral homoplasy possibly resulting from similar pollination systems. Assessing homoplasy, synapomorphies, and symplesiomorphies on a single major clade within Pleurothallidinae could be regarded as misleading because some characters such as a synsepally, glenion or viscidium are present in other clades of the subtribe. Therefore, we use here the *Lepanthes* clade and the combination of diagnostic traits coupled with ASRs as an example of an effective strategy for further characterizing its subclades in a novel way. Based on the results of the ASRs, members of these subclades can be either classified as genera, subgenera, or sections under the different views of systematists. Here, we propose the recognition of 14 genera in the *Lepanthes* clade based on a combination of molecular phylogenetics, and a morphological assessment of characters previously used in the taxonomy of Pleurothallidinae. We acknowledge that our findings can be interpreted differently, producing alternative hypotheses such as, for example, reducing the number of genera recognized here even further or delimiting them using morphological similarities instead of differences. All formerly proposed classifications of the Pleurothallidinae have been published without assessing the evolution of any of the traits. In contrast, and for the first time, we propose a classification based on an ASRs. The strategy followed in our study allows for the detection of those potentially linked to pollination or environmental pressures that can lead to mistaken delimitations. Thus, future research should focus on assessing novel morphological traits not previously used for classifications and including continuous characters that complement the discrete ones. For instance, micro-morphological traits such as cell wall lignification might be promising because they have been overlooked by orchid taxonomists.

Concerning species sampling, future studies should focus on members of *Trichosalpinx* subgenus *Xenia*, which are extremely scarce in the wild but need to be phylogenetically evaluated to obtain a complete evolutionary scenario for the *Lepanthes* clade. Following morphological observations, we suspect that some members might be related to *Lepanthopsis* and allies but this hypothesis needs further evaluation. Then, it is desirable to increase sampling in other groups such as *Lepanthopsis* (mainly the Antillean species) and *Tubella* because of floral similarities. Our phylogenetic framework and methodological approach enables the discovery of useful traits for generic classifications and paves the way for more comprehensive assessments on generic delimitations of similar recalcitrant lineages based on DNA sequences and morphological characters to further improve the systematics of the subtribe.

## Methods

### Taxon sampling

We sampled 148 accessions of 120 species from every generic name erected in the group. We included *Anathallis* (six spp)., *Draconanthes* (one sp.), *Frondaria* (one sp.), *Gravendeelia* (one sp.), *Lankesteriana* (five spp.), *Lepanthes* (61 spp.), *Lepanthopsis* (six spp.), *Opilionanthe* (one sp.), *Pendusalpinx* (eight spp.), *Pseudolepanthes* (two sp.), *Stellamaris* (one sp.), *Trichosalpinx* (eigth spp.), *Tubella* (14 spp.) and *Zootrophion* (six spp.). The type species was sampled for *Draconanthes*, *Frondaria*, *Gravendeelia*, *Lankesteriana*, *Lepanthopsis*, *Opilionanthe*, *Pendusalpinx*, *Pseudolepanthes* and, *Stellamaris*. Members of the *Trichosalpinx* subgenus *Xenia* Luer (five spp.) were not sampled due to unavailability of material. Voucher information, NCBI GenBank accessions, and references for each DNA sequence are listed in Appendix S1. A total of 88 sequences were newly generated (49 nrITS and 39 *mat*K) and complemented these with sequences from previous studies^9,12,29^. *Acianthera cogniauxiana* (Schltr.) Pridgeon & M.W.Chase and *Acianthera fenestrata* (Barb.Rodr.) Pridgeon & M.W.Chase were chosen as outgroups based on Pridgeon^9^.

### Phenotypic character selection

We scored 18 macro-morphological characters (Table 3) that have been considered taxonomically informative or ecologically important to characterize some of the genera (see references in Table 3). Data were obtained by direct observations from herbarium material (CR, AMES, JBL, K, L, PMA, UCH, W herbaria) and living material collected in the field or cultivated at Lankester Botanical Garden, the Hortus botanicus Leiden or private orchid collections. Observations were complemented with morphological data compiled from monographs on the Pleurothallidinae^13,16,22,24,30,40–43^ and with digital documentation (photographs and drawings) from the herbarium JBL. We generated additional macro-morphological data with a scanning electron microscope (SEM) using fixed flowers dehydrated in a series of ethanol solutions (70–96%– ≥99.9%) and acetone ≥99.8%. Critical-point drying was performed in an automated critical point dryer Leica EM CPD300 (Leica Microsystems, Wetzlar, Germany) following the manufacturer’s procedures. Samples were sputter-coated with 20 nm of Pt/Pd in a Quorum Q150TS sputter-coater and observed with a JEOL JSM-7600F (Tokyo, Japan) field emission SEM, at an accelerating voltage of 10 kV. For macro-photography we used a Nikon® D7100 (Tokyo, Japan) digital camera and a PB-6 Nikon bellows. We edited the images in Adobe Photoshop® CC (Adobe Systems Inc., California, U.S.A).

**Table 3:** Characters and scoring of the 18 morphological traits assessed with ancestral character estimations and the main references illustrating or discussing these characters.

Characters	States	References
Habit	(0) caespitose; (1) creeping	^13, 71, 72^
Ramicauls	(0) non-proliferating; (1) proliferating	^13, 71, 72^
Ramicauls’ bracts	(0) unornamented-papyraceous; (1) ornamented; (2) unornamented-foliaceous	^40, 73^
Inflorescence	(0) simultaneously flowering; (1) successively flowering	^13, 39^
Inflorescence length	(0) shorter than leaves; (1) longer than leaves	^13, 39^
Flowers	(0) fully opening; (1) bud-like	^74^
Dorsal sepal concavity	(0) concave; (1) flattened	^16, 24^
Synsepal	(0) absent; (1) present	^13, 24, 30^
Sepal shape	(0) oblong-acute; (1) ovate-acuminate (2) ovate-acute	^13, 16, 24^
Petals shape	(0) dissimilar; (1) subsimilar	^13, 16, 30^
Lip shape	(0) laminar; (1) bilobed	^16, 24^
Lip motility	(0) motile; (1) sessile	^16, 26^
Glenion of the lip	(0) absent; (1) present	^40^
Appendix of the lip	(0) absent; (1) present	^24^
Column foot	(0) absent; (1) present	^13, 75^
Stigma shape	(0) entire; (1) bilobed	^40, 73^
Anther position	(0) ventral; (1) dorsal	^24^
Pollinaria-associated structures	(0) with caudicles; (1) with caudicles + viscidium	^17, 76^

### DNA extraction

We extracted total genomic DNA from about 50–100 mg of silica gel dried leaf/flower tissue. Each sample was placed in 2 ml Eppendorf® tube with three glass beads (7 mm) and sterile sand. The tubes were frozen in liquid nitrogen for about 1–2 minutes and powdered in a Retsch MM 300 shaker for 3 minutes. We followed the 2 × CTAB (Hexadecyltrimethylammonium bromide) protocol for isolating DNA^44^. DNA was quantified with a Qubit 3.0 Fluorometer (TermoFischer Scientific®).

### Amplification, sequencing and alignment

The polymerase chain reaction (PCR) mixture, the primers for the nrITS (17SE and 26SE)^45^ and plastid *mat*K (2.1 aF and 5 R)^9^ regions and amplification profiles followed. Sanger sequencing of both regions was conducted by BaseClear (https://www.baseclear.com) on an ABI 3730xl genetic analyzer (Applied Biosystems, Foster City, California, U.S.A). Sequences were deposited in NCBI GenBank (Appendix S1). We used Geneious® R9 (Biomatters Ltd., Auckland, New Zealand^46^) for the editing of chromatograms and pairwise alignment. Sequences were aligned in the online MAFFT platform (Multiple Alignment using Fast Fourier Transform, http://mafft.cbrc.jp/alignment/server/) using default settings. We adjusted and trimmed the resulting alignment manually. The concatenated dataset (nrITS + *mat*K) was built with Sequence Matrix v100.0^47^. When *mat*K sequences were not available, they were included as missing data in the concatenated matrix.

### Phylogenetic analyses

We analyzed the individual and concatenated datasets of nrITS and *mat*K with Bayesian inference (BI), maximum likelihood (ML) and maximum parsimony (MP) analyses. The model of evolution and the parameters were calculated using the Akaike Information Criterion (AIC) in jModelTest2 v2.1.7^48^. All analyses were run in the CIPRES Science Gateway V. 3.1 (http://www.phylo.org/sub_sections/portal/)^49^. To evaluate the incongruence between plastid and nuclear datasets we followed the pipeline implemented^12^ using the Procrustean Approach to Cophylogeny (PACo) application^27^ in R (http://datadryad.org/review?doi=doi:10.5061/dryad.q6s1f). This procedure identifies potential conflicting outliers contributing to incongruent phylogenies. The *mat*K sequences from the retrieved conflicting terminals were removed and replaced by missing data because inferences derived from plastid markers are usually more in conflict with morphological observations as compared with inferences derived from nuclear markers^50^. A new concatenated matrix was re-aligned using the cleaned *mat*K dataset and then analyzed with BI, ML, and MP approaches. These analyses were contrasted with the original inferences from concatenated datasets.

We performed the Bayesian inference analyses with MrBayes v.3.2.6 on XSEDE^51^ with the following parameters: number of generations Ngen = 50 × 10^6^ for the combined and individual datasets, number of runs (nruns = 2), number of chains to run (nchains = 4), temperature parameter (temp = 2) and sampling frequency of 1000 yielding 50,001 trees per run. The log files from MrBayes were inspected in Tracer v.1.6 to check the convergence of independent runs (i.e. with estimated sample size (ESS) > 200). The initial 25% of trees were discarded as burn-in and the resulting trees were used to obtain a 50% majority-rule consensus tree. Maximum likelihood analyses were performed with RAxML-HPC2 on XSEDE (8.2.10)^52^ choosing the GTRGAMMA model for bootstrapping and 1000 bootstrap iterations. Parsimony analyses were performed with PAUPRat: Parsimony ratchet searches using PAUP*^53–55^ with 1000 ratchet repetitions, seed value = 0, 20% percent of characters to perturb (pct = 20), original weights 1 for all characters (wtmode = uniform) and a tree bisection-reconnection branch swapping algorithm (swap = TBR). The 50% majority rule consensus trees for ML and MP were obtained with PAUP v4.0a152. and observed in FigTree v.1.3.1. The statistical support of the clades was evaluated with the values of posterior probability (PP) for BI reconstruction, bootstrap for ML (MLB) and parsimony bootstrap for MP (PBP). The PPs were added to the branches on the Bayesian 50% majority-rule consensus tree with internal support values shown for ML and MP when the same topology was retrieved. We considered clades with PBP ≥ 70%, LPB ≥85% and PP ≥ 0.95% as well supported. To investigate phylogenetic relationships among genera, we also conducted a network analysis with 3000 tree replicates of the BI inference of the combined dataset in Splits Tree4 v.4.11.3^56^ with a 0.20 cutoff value. Resulting trees were manipulated with R programming language^57^ under R Studio^58^ using the packages APE, ggtree, and phytools^59–61^. Final trees were edited in Adobe® Illustrator CC (Adobe Systems Inc., California, U.S.A).

To obtain ultrametric trees for the character evolution assessments we estimated the divergence times in BEAST v.1.8.2 using the CIPRES Science Gateway^49^. The clock-likeness of the data was tested by observing the coefficient of variation (CV) of relaxed clock models. Speciation tree model selection was achieved by executing the Bayes factor test on Yule process (Y), birth death-process (BD) and birth-death-incomplete sampling (BDIS) models under strict and uncorrelated lognormal molecular clock models. For each model, we assigned a normal prior distribution of 16.45 (±2.5 standard deviations) Mya to the root node of the *Lepanthes* clade and 12.93 (±2.5 standard deviations) Mya to the node of *Zootrophion* with the remainder of the members of the *Lepanthes* clade using the values calculated from the fossil-calibrated chronogram of the Pleurothallidinae^12^. We performed two MCMC with 50 × 10^6^ generations and sampling every 1,000 generations with a Marginal likelihood estimation (MLE) of 50 path steps, 10 × 10^5^ length of chains and log likelihood for every 1,000 generations. We inspected the convergence of independent runs size in Tracer v.1.6 as explained above. To compare the divergence time estimates among the speciation models (Y, BD, and BDIS) we used Bayes factors calculated with marginal likelihood using stepping stone sampling derived from the MLE path sampling.

### Ancestral state reconstruction (ASRs)

Ancestral state reconstructions were assessed with ML, stochastic character mapping (SCM), and BI using phylograms and ultrametric trees. For the ML approach, we explored the following models: equal rates (ER), symmetrical (SYM) and all rates different (ARD). We relied on the re-rooting method of Yang^62^ and the function ACE implemented in the R-package phytools. The best-fitting model was selected by comparing the log-likelihoods among these models using likelihood ratio tests. Scaled likelihoods at the root and nodes were plotted in the time-calibrated consensus phylogenetic tree. For the stochastic mapping analyses based on joint sampling, we performed 100 replicates on 100 randomly selected trees (10,000 mapped trees) from the best fitting time-calibrated BEAST analysis. The trees were randomly selected using the R function *samples*.*trees* (http://coleoguy.blogspot.de/2012/09/randomly-sampling-trees.html). Results of transitions and the proportion of time spent in each state were calculated and summarized in phytools with the functions *make*.*simmap* and *describe*.*simmap*^61,63^. These analyses were performed following the scripts by Portik and Blackburn (2016)^64^. ML and BI analyses were executed in the program BayesTraits V3^65–67^. To account for phylogenetic uncertainty, ancestral character estimates were calculated using a randomly sampled set of 1,000 trees from the post burnin sample of the ﻿50,000 ultrametric trees obtained from the best fitting time-calibrated BEAST analysis as described above. We used the option *AddNode* for reconstruction of internal nodes of interest comprising every generic group of the *Lepanthes* clade and the root node. For the ML approach, we used the method *Multistate* with 10 ML attempts per tree and 20,000 evaluations in order to preliminary assess prior distributions. For the BI, we chose the method *Multistate* and MCMC parameters of 30,010,000 iterations, sample period of 1,000, burnin of 10,000, auto tune rate deviation and stepping stones 100 10,000. We used the method Reversible-Jump MCMC with hyper-prior exponential to assess the best fitting models in proportion to their posterior probabilities according to the MCMC approach. We chose the hyper-prior approach as recommended by Meade and Pagel^67^ in order to reduce the arbitrariness when choosing priors. Therefore, we selected the option reversible jump hyper-prior exponential with prior distribution set according to the transition ranges obtained from a preliminary ML analysis^68^. The input files for BayesTraits V3 were partially constructed with Wrappers to Automate the Reconstruction of Ancestral Character States ﻿(WARACS)^69^. The BayesTraits outputs files were analyzed in R with the BayesTraits wrapper (btw) by Randi H Griffin (http://rgriff23.github.io/projects/btw.html) and other functions from btrtools and BTprocessR (https://github.com/hferg). The MCMC stationarity of parameters (ESS values > 200) and convergence of chains were checked in Tracer v1.6.0 and plotted in R with the packages coda^70^ and the function *mcmcPlots* of BTprocessR. We reconstructed the ancestral states for all nodes of the tree and plotted the mean probabilities retrieved at each node with phytools.

## Supplementary information

Supporting Information
