# Supplementary material for: Phylogenetic comparative methods improve the selection of characters for generic delimitations in a hyperdiverse Neotropical orchid clade
Source: Sci Rep. 2019 Oct 22;9:15098. doi: 10.1038/s41598-019-51360-0 (PMC6805863; doi:10.1038/s41598-019-51360-0)

SUPPORTING INFORMATION

Manuscript title:

Phylogenetic comparative methods improve the selection of characters for generic delimitations in a hyperdiverse Neotropical orchid lineage

Authors:

Diego Bogarín[1,2,3,*], Oscar A. Pérez-Escobar[4,5], Adam P. Karremans[1,3], Melania Fernandez[1,6], Jaco Kruizinga[7], Franco Pupulin[1,8], Erik Smets[3,10,11], and Barbara Gravendeel[3,9,10]

[1]Jardín Botánico Lankester, Universidad de Costa Rica, Cartago, Costa Rica
[2]Herbarium UCH, Universidad Autónoma de Chiriquí, Chiriquí, Panama
[3]Naturalis Biodiversity Center, Endless Forms group, Leiden, The Netherlands
[4]Comparative Plant and Fungal Biology, Royal Botanic Gardens, Kew, Richmond, Surrey TW9 3AB, UK
[5]Gothenburg Global Biodiversity Centre, Gothenburg, Sweden
[6] Department of Plant and Soil Science, Texas Tech University, Lubbock, TX 79409, U.S.A.
[7]Hortus botanicus, Leiden University, Leiden, The Netherlands
[8] Harvard University Herbaria, 22 Divinity Avenue, Cambridge, Massachusetts, U.S.A.; Marie Selby Botanical Gardens, 811 South Palm Avenue, Sarasota, Florida 34236, U.S.A.
[9]University of Applied Sciences Leiden, Faculty of Science and Technology, Leiden, The Netherlands
[10]Institute of Biology Leiden, Leiden University, Leiden, The Netherlands
[11]KU Leuven, Ecology, Evolution and Biodiversity Conservation, Leuven, Belgium
[*]Corresponding author. E-mail: diego.bogarin@ucr.ac.cr

Appendix S1-S15:

Appendix S1. Species, length of sequences in matrices, voucher information, Genbank accession numbers, references and incongruent terminals of the individuals analyzed.

Taxon	Terminal name	Total length (bp)	ITS length (bp)	MATK length (bp)	Voucher	Genbank (ITS)	Genbank (matK)	Incongruent terminal (ML)	Incongruent terminal (BI)
Acianthera cogniauxiana (Schltr.) Pridgeon & M.W.Chase	Acianthera_cogniauxiana_AK5879	1433	756	677	AK5879	KR816545	KR816554		
Acianthera fenestrata (Barb.Rodr.) Pridgeon & M.W.Chase	Acianthera_fenestrata_JLOrchids_sn	1564	798	766	JLOrchids sn	AF262857	AF265468		
Anathallis burzlaffiana (Luer & Sijm) Luer	Anathallis_burzlaffiana_AK4857	1564	798	766	AK4857	KC425727	KC425857		

Anathallis lewisiae (Ames) Solano & Soto Arenas	Anathallis_lewisiae_DB1056	1564	798	766	DB1056	KC425733	KC425858		
Anathallis linearifolia (Cogn.) Pridgeon & M.W.Chase	Anathallis_linearifolia_JH2336	1564	798	766	JH2336	AF262869	AF265473		
Anathallis pabstii (Garay) Pridgeon & M.W.Chase	Anathallis_pabstii_AK4821	1564	798	766	AK4821	KC425737	KC425859		
Anathallis peroupavae (Hoehne & Brade) F.Barros	Anathallis_peroupavae_AK5759	1492	797	695	AK5759	KF747837	MK306408		
Anathallis rabei (Foldats) Luer (1)	Anathallis_rabei_AK4794	1564	798	766	AK4794	KC425738	KC425860		
Anathallis rabei (Foldats) Luer (2)	Anathallis_rabei_DB12050	1558	797	761	DB12050	MK306359	MK306409		
Draconanthes aberrans (Schltr.) Luer	Draconanthes_aberrans_AK5978	1487	797	690	AK5978	KY988810	KY988630	X	X
Frondaria caulescens (Lindl.) Luer	Frondaria_caulescens_CL18778	1562	796	766	CL18778	AF262896	AF265471	X	
Gravendeelia chamaelepanthes (Rchb.f.) Bogarín & Karremans (1)	Gravendeelia_chamaelepanthes_AK4815	797	797	-	AK5786	MK306362	-		
Gravendeelia chamaelepanthes (Rchb.f.) Bogarín & Karremans (2)	Gravendeelia_chamaelepanthes_AP127	1564	798	766	AP127	MK306363	MK306410	X	X
Gravendeelia chamaelepanthes (Rchb.f.) Bogarín & Karremans (3)	Gravendeelia_chamaelepanthes_DB11881	797	797	-	DB11881	MK306364	-		
Gravendeelia chamaelepanthes (Rchb.f.) Bogarín & Karremans (4)	Gravendeelia_chamaelepanthes_PL459a	797	797	-	PL459a	MK306365	-		
Lankesteriana barbulata (Lindl.) Karremans (1)	Lankesteriana_barbulata_AK5187	797	797	-	AK5187	MK306366	-		
Lankesteriana barbulata (Lindl.) Karremans (2)	Lankesteriana_barbulata_AK5447	797	797	-	AK5447	MK306367	-		
Lankesteriana barbulata (Lindl.) Karremans (3)	Lankesteriana_barbulata_AK5750	797	797	-	AK5750	KF747834	-		
Lankesteriana barbulata (Lindl.) Karremans (4)	Lankesteriana_barbulata_DB8606	797	797	-	DB8606	KC425726	-		
Lankesteriana casualis (Ames) Karremans	Lankesteriana_casualis_AK6190	1515	797	718	AK6190	KY988821	KY988638		
Lankesteriana cuspidata (Luer) Karremans	Lankesteriana_cuspidata_DB9619	797	797	-	DB9619	KF747835	-		
Lankesteriana duplooyi Luer & Sayers) Karremans	Lankesteriana_duplooyi_AK4888	797	797	-	AK4888	KF747836	-		

Lankesteriana fractiflexa (Ames & C.Schweinf.) Karremans	Lankesteriana_fractiflexa_DB8988	797	797	-	DB8988	KC425729	-		
Lepanthes ankistra Luer & Dressler	Lepanthes_ankistra_AK6147	1564	798	766	AK6147	KY988822	KY988639		
Lepanthes atrata Endres ex Luer	Lepanthes_atrata_DB11053	1564	798	766	DB11053	KY988823	KY988640	X	X
Lepanthes blephariglossa Schltr.	Lepanthes_blephariglossa_DB9604	1564	798	766	DB9604	KY988824	KY988641	X	X
Lepanthes blepharistes Rchb.f.	Lepanthes_blepharistes_FP8720	1549	783	766	FP8720	KY988829	KY988646		
Lepanthes bradei Schltr.	Lepanthes_bradei_AK5267	1564	798	766	AK5267	KY988830	KY988647		
Lepanthes brunnescens Luer	Lepanthes_brunnescens_DB2994	1564	798	766	DB2994	KY988831	KY988648		
Lepanthes calliope Luer & Hirtz	Lepanthes_calliope_DB11873	1564	798	766	DB11873	KY988832	KY988649		
Lepanthes calodyction Hook.	Lepanthes_calodyction_DB11872	1564	798	766	DB11872	KY988833	KY988650		
Lepanthes candida Endres ex Luer	Lepanthes_candida_DB11656	1564	798	766	DB11656	KY988834	KY988651		
Lepanthes caprimulgus Luer	Lepanthes_caprimulgus_DB11874	1564	798	766	DB11874	KY988835	KY988652		
Lepanthes cascajalensis Ames	Lepanthes_cascajalensis_DB4836	1564	798	766	DB4836	KY988836	KY988653	X	X
Lepanthes cloesii Luer	Lepanthes_cloesii_DB11877	1564	798	766	DB11877	KY988837	KY988654	X	X
Lepanthes confusa Ames & C.Schweinf.	Lepanthes_confusa_DB3087	1550	784	766	DB3087	KY988838	KY988655	X	X
Lepanthes cribii Pupulin	Lepanthes_cribii_FP8711	1564	798	766	FP8711	KY988839	KY988656		
Lepanthes cuspidata Luer	Lepanthes_cuspidata_AK6239	1564	798	766	AK6239	KY988840	KY988657		
Lepanthes demissa Luer	Lepanthes_demissa_DB2981	1545	779	766	DB2981	KY988842	KY988659		
Lepanthes dikoensis Bogarín & C.M.Sm.	Lepanthes_dikoensis_DB1625	1564	798	766	DB1625	KY988843	KY988660	X	X
Lepanthes disticha (A.Rich.& Galeotti) Garay & R.E.Schult	Lepanthes_disticha_AK4589	1564	798	766	AK4589	KY988845	KY988661		X
Lepanthes dolabriformis Luer	Lepanthes_dolabriformis_DB10375	1564	798	766	DB10375	KY988846	KY988662		
Lepanthes droseroides Luer	Lepanthes_droseroides_MF945	1564	798	766	MF945	KY988848	KY988664		
Lepanthes elata Rchb.f.	Lepanthes_elata_DB10554	1564	798	766	DB10554	KY988850	KY988666		
Lepanthes elegans Luer	Lepanthes_elegans_DB7606	1564	798	766	DB7606	KY988853	KY988669	X	X
Lepanthes eximia Ames	Lepanthes_eximia_DB9600	1564	798	766	DB9600	KY988854	KY988670		
Lepanthes ferrelliae Luer	Lepanthes_ferrelliae_FP8806	1564	798	766	FP8806	KY988855	KY988671		X
Lepanthes gargantua Rchb.f.	Lepanthes_gargantua_DB11868	1564	798	766	DB11868	KY988856	KY988672	X	X
Lepanthes glicensteinii Luer	Lepanthes_glicensteinii_AK5818	1512	761	751	AK5818	KY988857	KY988673		

Lepanthes gustavo-romeroi Archila	Lepanthes_gustavoromeroi_DB11293	1564	798	766	DB11293	KY988859	KY988675		X
Lepanthes hermansii Luer	Lepanthes_hermansii_FP8611	1564	798	766	FP8611	KY988860	KY988676	X	
Lepanthes horichii Luer	Lepanthes_horichii_AK5507	1564	798	766	AK5507	KY988861	KY988677	X	X
Lepanthes kleinii Bogarín & Pupulin	Lepanthes_kleinii_FP7999	1564	798	766	FP7999	KY988862	KY988678	X	X
Lepanthes latisepala Ames & C.Schweinf.	Lepanthes_latisepala_DB11102	1564	798	766	DB11102	KY988863	KY988679		
Lepanthes lindleyana Oerst.& Rchb.f.	Lepanthes_lindleyana_DB8392	1564	798	766	DB8392	KY988865	KY988681		X
Lepanthes machogaffensis Pupulin & D.Jiménez	Lepanthes_machogaffensis_DB10522	1564	798	766	DB10522	KY988867	KY988683		
Lepanthes maduroi Luer	Lepanthes_maduroi_DB2974	1564	798	766	DB2974	KY988868	KY988684		
Lepanthes martineae Luer & Cloes	Lepanthes_martinae_DB11878	1564	798	766	DB11878	KY988869	KY988685		
Lepanthes matamorosii Pupulin & Bogarín	Lepanthes_matamorosii_AK5661	1564	798	766	AK5661	KY988870	KY988686		
Lepanthes mentosa Luer	Lepanthes_mentosa_DB11533	1556	790	766	DB11533	KY988871	KY988687		X
Lepanthes monteverdensis Luer & R.Escobar	Lepanthes_monteverdensis_DB11482	1564	798	766	DB11482	KY988872	KY988688		
Lepanthes montis-narae Pupulin, Bogarín & C.M.Sm.	Lepanthes_montisnarae_AK6536	1564	798	766	AK6536	KY988873	KY988689	X	X
Lepanthes myiophora Luer	Lepanthes_myiophora_FP7971	1564	798	766	FP7971	KY988874	KY988690		X
Lepanthes mystax Luer & R.Escobar	Lepanthes_mystax_DB11446	1564	798	766	DB11446	KY988875	KY988691		X
Lepanthes nycteris Luer & R.Vásquez	Lepanthes_nycteris_DB11875	1564	798	766	DB11875	KY988876	KY988692		
Lepanthes olmosii Bogarín	Lepanthes_olmosii_DB3005	1564	798	766	DB3005	KY988877	KY988693	X	X
Lepanthes queveriensis Bogarín & Pupulin	Lepanthes_queveriensis_DB10854	1564	798	766	DB10854	KY988879	KY988695		
Lepanthes rafaeliana Pupulin	Lepanthes_rafaeliana_DB11658	1564	798	766	DB11658	KY988880	KY988696		X
Lepanthes regularis Luer	Lepanthes_regularis_DB7756	1564	798	766	DB7756	KY988881	KY988697		
Lepanthes ribes Luer	Lepanthes_ribes_AP153	1526	769	757	AP153	KY988882	KY988698	X	X
Lepanthes saltatrix Luer & Hirtz	Lepanthes_saltatrix_HBL26	1564	798	766	HBL_26	KY988883	KY988699		
Lepanthes sandiorum Bogarín & Karremans	Lepanthes_sandiorum_DB8171	1564	798	766	DB8171	KY988884	KY988700		
Lepanthes siboei Bogarín & D.Jiménez	Lepanthes_siboei_DB9927	1564	798	766	DB9927	KY988885	KY988701		X

Lepanthes sijmii Luer & Sijm	Lepanthes_sijmii_DB11879	1564	798	766	DB11879	KY988886	KY988702		X
Lepanthes spadariae Pupulin	Lepanthes_spadariae_DB11676	1564	798	766	DB11676	KY988887	KY988703		
Lepanthes stenorhyncha Luer	Lepanthes_stenorrhyncha_DB11517	1564	798	766	DB11517	KY988888	KY988704		
Lepanthes terborchii Luer & Sijim	Lepanthes_terborchii_DB11876	1564	798	766	DB11876	KY988889	KY988705		
Lepanthes tristis Bogarín & Pupulin	Lepanthes_tristis_DB11294	1564	798	766	DB11294	KY988890	KY988706	X	X
Lepanthes turialvae Rchb.f.	Lepanthes_turialvae_DB2394	1564	798	766	DB2394	KY988891	KY988707	X	X
Lepanthes variabilis C.M.Sm., Pupulin & D.Jiménez	Lepanthes_variabilis_AK6380	1564	798	766	AK6380	KY988892	KY988708		
Lepanthes velosa Luer & Hirtz	Lepanthes_velosa_FP6504	1564	798	766	FP6504	KY988893	KY988709		
Lepanthes whittenii Pupulin & Bogarín	Lepanthes_whittenii_MF909	1564	798	766	MF909	KY988895	KY988711		
Lepanthes williamsii Salazar & Soto Arenas	Lepanthes_williamsii_DB11292	1564	798	766	DB11292	KY988896	KY988712	X	X
Lepanthes woodburyana Stimson	Lepanthes_woodburyana_JH2931	1564	798	766	JH2931	AF262890	AF265472	X	X
Lepanthopsis apoda (Garay & Dunst.) Luer	Lepanthopsis_apoda_AP126	1564	798	766	AP126	KF747841	MK306411	X	X
Lepanthopsis astrophora Garay (1)	Lepanthopsis_astrophora_AK5766	1505	797	708	AK5766	MK306368	MK306412		
Lepanthopsis floripecten (Rchb.f.) Ames (1)	Lepanthopsis_floripecten_AK3006	1564	798	766	DB7795	KY988898	MK306413		
Lepanthopsis floripecten (Rchb.f.) Ames (2)	Lepanthopsis_floripecten_CvdB2063	1548	797	751	AK3006	KY988897	KY988713		
Lepanthopsis floripecten (Rchb.f.) Ames (3)	Lepanthopsis_floripecten_DB7795	773	773	-	CvdB2063	JQ306336	-		
Lepanthopsis obliquipetala (Ames & C.Schweinf.) Luer	Lepanthopsis_obliquipetala_AK5626	1564	798	766	AK5626	MK306369	MK306414		
Lepanthopsis prolifera Garay	Lepanthopsis_prolifera_AK5722	797	797	-	AK5722	MK306370	-		
Lepanthopsis ubanguii Luer	Lepanthopsis_ubangui_HBL	797	797	-	HBL_sn	MK306371	-		
Opilionanthe manningii (Luer) Karremans & Bogarín	Opilionanthe_manningii_DB11883	1564	798	766	DB11883	MK306372	MK306415	X	X
Pendusalpinx berlineri (Luer) Karremans & Mel.Fernández (1)	Pendusalpinx_berlineri_AK5770	1518	797	721	AK5770	KY988984	KY988792		
Pendusalpinx berlineri (Luer) Karremans & Mel.Fernández (1)	Pendusalpinx_berlineri_JH1605	1561	795	766	JH1605	AF262900	AF265475		

Pendusalpinx dependens (Luer) Karremans & Mel.Fernández (1)	Pendusalpinx_dependens_CvdB2011	797	797	-	CvdB2011	JQ306456	-		
Pendusalpinx dependens (Luer) Karremans & Mel.Fernández (2)	Pendusalpinx_dependens_AK4866	797	797	-	AK4866	MK306373	-		
Pendusalpinx patula (Luer) Karremans & Mel.Fernández	Pendusalpinx_patula_HBL41	797	797	-	HBL_41	MK306374	-		
Pendusalpinx sijmii (Luer) Karremans & Mel.Fernández	Pendusalpinx_sijimii_AK5994	1540	797	743	AK5994	KY988993	KY988801		
Pendusalpinx sp.	Pendusalpinx_sp_HBL39	797	797	-	HBL_39	MK306375	-		
Pendusalpinx sp.	Pendusalpinx_sp_HBL40	797	797	-	HBL_40	MK306376	-		
Pendusalpinx vasquezii (Luer) Karremans & Mel.Fernández	Pendusalpinx_vasquezii_AK6496	797	797	-	AK6496	MK306377	-		
Pseudolepanthes colombiae Archila (1)	Pseudolepanthes_colombiae_AK6455	1552	797	755	AK6455	MK306378	MK306416		
Pseudolepanthes colombiae Archila (2)	Pseudolepanthes_colombiae_AK6456	1564	798	766	AK6456	MK306379	MK306417		
Pseudolepanthes colombiae Archila (3)	Pseudolepanthes_colombiae_AK6458	1564	798	766	AK6458	MK306380	MK306418		
Stellmaris pergrata (Ames) Mel.Fernández & Bogarín (1)	Stellamaris_pergrata_DB12038	1551	797	754	DB12038	MK306381	MK306419		
Stellmaris pergrata (Ames) Mel.Fernández & Bogarín (2)	Stellamaris_pergrata_DB5635	1564	798	766	DB5635	MK306382	MK306420		
Stellmaris pergrata (Ames) Mel.Fernández & Bogarín (3)	Stellamaris_pergrata_DB6502	1564	798	766	DB6502	MK306383	MK306421		
Trichosalpinx blaisdellii (S.Watson) Luer (1)	Trichosalpinx_blaisdellii_AK5308	1547	781	766	AK5308	MK306384	MK306422		
Trichosalpinx blaisdellii (S.Watson) Luer (2)	Trichosalpinx_blaisdellii_DB292	1564	798	766	DB292	MK306385	MK306423		
Trichosalpinx blaisdellii (S.Watson) Luer (3)	Trichosalpinx_blaisdellii_K19977412	1564	798	766	K19977412	MK306386	MK306424		
Trichosalpinx blaisdellii (S.Watson) Luer (4)	Trichosalpinx_blaisdellii_MF2	1564	798	766	MF2	MK306387	MK306425		
Trichosalpinx memor (Rchb.f.) Luer (1)	Trichosalpinx_memor_DB6462	1564	798	766	DB6462	MK306388	MK306426		
Trichosalpinx memor (Rchb.f.) Luer (2)	Trichosalpinx_memor_DB8696	1564	798	766	DB8696	KY988987	KY988795		

Trichosalpinx minutipetala (Ames & C.Schweinf.) Luer (1)	Trichosalpinx_minutipetala_FP7581	1564	798	766	FP7581	MK306389	MK306427		
Trichosalpinx minutipetala (Ames & C.Schweinf.) Luer (2)	Trichosalpinx_minutipetala_MF446	1522	797	725	MF446	MK306390	MK306428		
Trichosalpinx orbicularis (Lindl.) Luer (1)	Trichosalpinx_orbicularis_AR6474	1564	798	766	AR6474	KY988989	KY988797		
Trichosalpinx orbicularis (Lindl.) Luer (2)	Trichosalpinx_orbicularis_JH1349	1564	798	766	JH1349	AF262886	AF265476		
Trichosalpinx orbicularis (Lindl.) Luer (3)	Trichosalpinx_orbicularis_MF65b	1564	798	766	MF65b	MK306391	MK306429		
Trichosalpinx pringlei (Schltr.) Luer (1)	Trichosalpinx_pringlei_AK6463	1564	798	766	AK6463	MK306392	MK306430		
Trichosalpinx pringlei (Schltr.) Luer (2)	Trichosalpinx_pringlei_AK6706	1564	798	766	AK6706	MK306393	MK306431		
Trichosalpinx reflexa Mel.Fernández & Bogarín (1)	Trichosalpinx_reflexa_DB4075	1564	798	766	DB4075	KY988991	KY988799		
Trichosalpinx reflexa Mel.Fernández & Bogarín (2)	Trichosalpinx_reflexa_MF195	1564	798	766	MF195	KY988992	KY988800		
Trichosalpinx ringens Luer	Trichosalpinx_ringens_MF577	1564	798	766	MF577	MK306394	MK306432	X	X
Trichosalpinx rotundata (C.Schweinf.) Dressler	Trichosalpinx_rotundata_AK4386a	1564	798	766	AK4386a	MK306395	MK306433		
Tubella alabastra (Luer & R.Escobar) Archila	Tubella_alabastra_AK5540	1564	798	766	AK5540	MK306396	MK306434		
Tubella arbuscula (Lindl.) Archila	Tubella_arbuscula_DB8881	1508	797	711	DB8881	MK306397	MK306435		
Tubella cedralensis (Ames) Archila (1)	Tubella_cedralensis_AK6010	1564	798	766	AK6010	KY988985	KY988793		
Tubella cedralensis (Ames) Archila (2)	Tubella_cedralensis_FP7049	1564	798	766	FP7049	MK306398	MK306436		
Tubella dirhamphis (Luer) Archila	Tubella_dirhamphis_DB11882	1564	798	766	DB11882	MK306399	MK306437		
Tubella dura (Lindl.) Archila	Tubella_dura_HBL42	1549	797	752	HBL_42	MK306400	MK306438		
Tubella fruticosa (Luer) Archila	Tubella_fruticosa_JBL11580	1564	798	766	JBL11580	KY988986	KY988794		
Tubella notosibirica (T.Hashim.) Archila	Tubella_notosibirica_AP225	1530	797	733	AP225	MK306401	MK306439		
Tubella nymphalis (Luer) Archila	Tubella_nymphalis_AK5950	1557	791	766	AK5950	KY988988	KY988796		
Tubella parsonsii (Luer & Dod) Archila (1)	Tubella_parsonsii_AK3302	1551	797	754	AK3302	MK306402	MK306440		

Tubella parsonsii (Luer & Dod) Archila (2)	Tubella_parsonsii_AK3305	1564	798	766	AK3305	MK306403	MK306441		
Tubella pusilla (Kunth) Archila	Tubella_pusilla_DB11841	1564	798	766	DB11841	KY988990	KY988798		
Tubella robledorum (Luer) Archila	Tubella_robledorum_AK5491	1564	798	766	AK5491	MK306404	MK306442		
Tubella sp.	Tubella_sp_DB9739	1564	798	766	DB9739	MK306405	MK306443		
Tubella todziae (Luer) Archila (1)	Tubella_todziae_AK3983	1532	790	742	AK3983	MK306406	MK306444		
Tubella todziae (Luer) Archila (2)	Tubella_todziae_MF540	1564	798	766	MF540	MK306407	MK306445		
Zootrophion gracilentum (Rchb.f.) Luer	Zootrophion_gracillenthum_AK5282	1559	797	762	AK5282	KY988995	MK306446		
Zootrophion hirtzii Luer (1)	*Zootrophion*_hirtzii_AK4848	797	797	-	AK4848	MK306360	-		
Zootrophion hirtzii Luer (2)	*Zootrophion*_hirtzii_AK6503	797	797	-	AK6503	MK306361	-		
Zootrophion hypodiscus (Rchb.f.) Luer	Zootrophion_hypodiscus_JBL01480	1564	798	766	JBL01480	KY988997	MK306447		
Zootrophion machaqway A.Doucette & J.Portilla	Zootrophion_machaqway_AK6505	1524	797	727	AK6505	KY988998	KY988803		
Zootrophion vulturiceps (Luer) Luer	Zootrophion_vulturiceps_FP3960	1549	783	766	FP3960	KY989000	KY988804		
Zootrophion ximenae (Luer & Hirtz) Pfahl	*Zootrophion*_ximeniae_AK6502	1521	797	724	AK6502	KY989001	KY988805		

Appendix S2. Summary of matrix statistics and parsimony information, for the nrITS, *mat*K and combined datasets. MT=missing taxa compared to concatenated matrix, %IS = identical sites (percentage) in matrix, %PI = percentage of pairwise identity in matrix, % MC = percentage of missing characters in matrix, % gaps = percentage gap characters of the sequences in matrix, %GC = percentage of GC content in matrix, Model = models selected with AIC according to jModelTest2 v2.1.7, PIC=Parsimony informative characters, CI=consistency index, RI=retention index.

**Matrix**	**Species**	**Terminals**	**Length (bp)**	**MT**	**IS (%)**	**PI %**	**% MC**	**% gaps**	**%GC**	**Model**	**PIC**	**CI**	**RI**
matK	111	127	766	13	65.8	97.4	0	0.2	31.5	TVM+I+G	158	0.632	0.848
ITS	122	148	798	1	43.4	90.6	0.3	7.9	57.6	GTR+I+G	311	0.422	0.85
ITS+matK	122	148	1564	-	54.6	93.5	7.7	4.1	45.3	-	469	0.468	0.846

Appendix S3. Consensus trees inferred from concatenated datasets with BI, ML and MP.

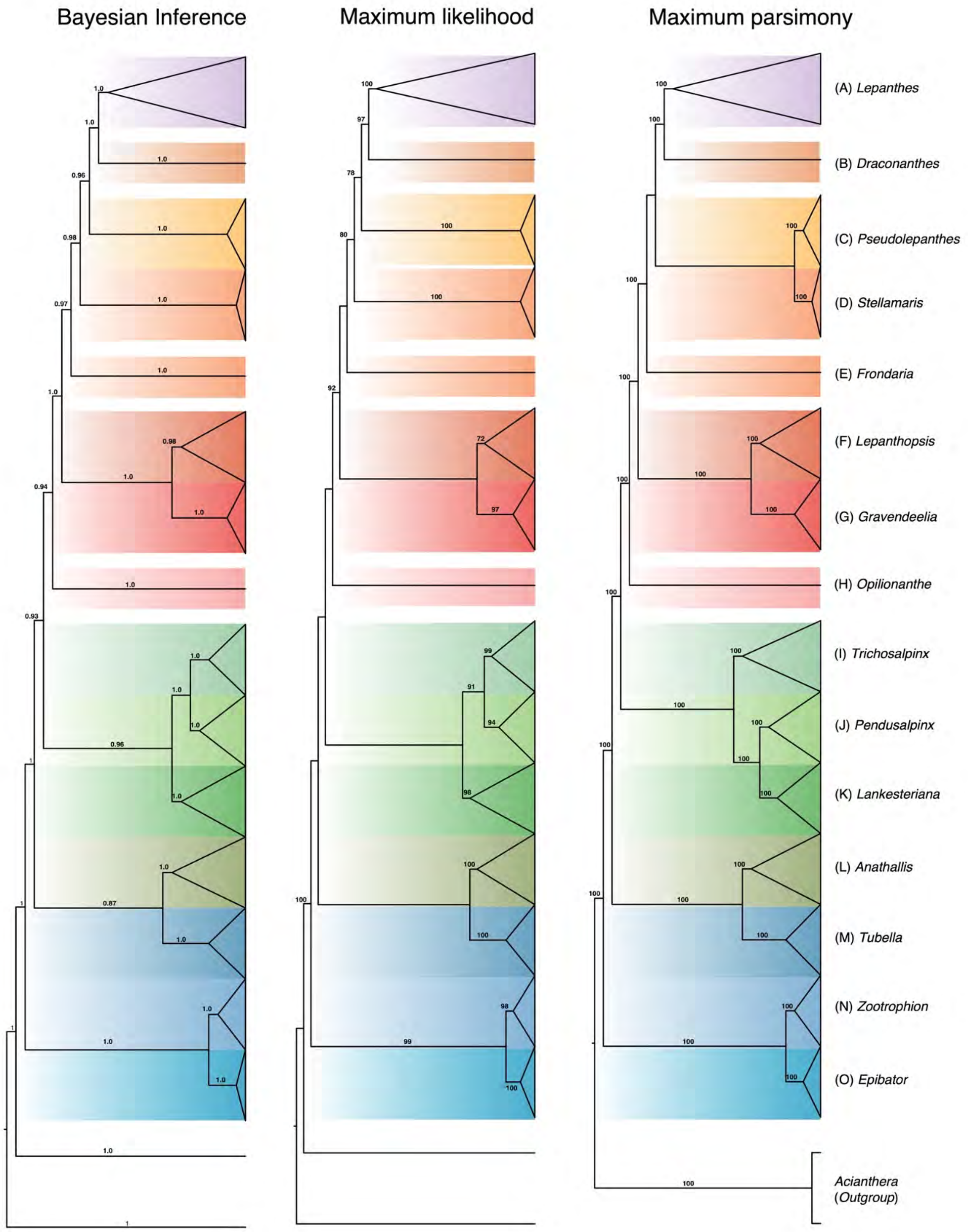

Appendix S4. Consensus ML tree inferred from A. ITS dataset B. *mat*K dataset.

A. ITS-ML concensus tree

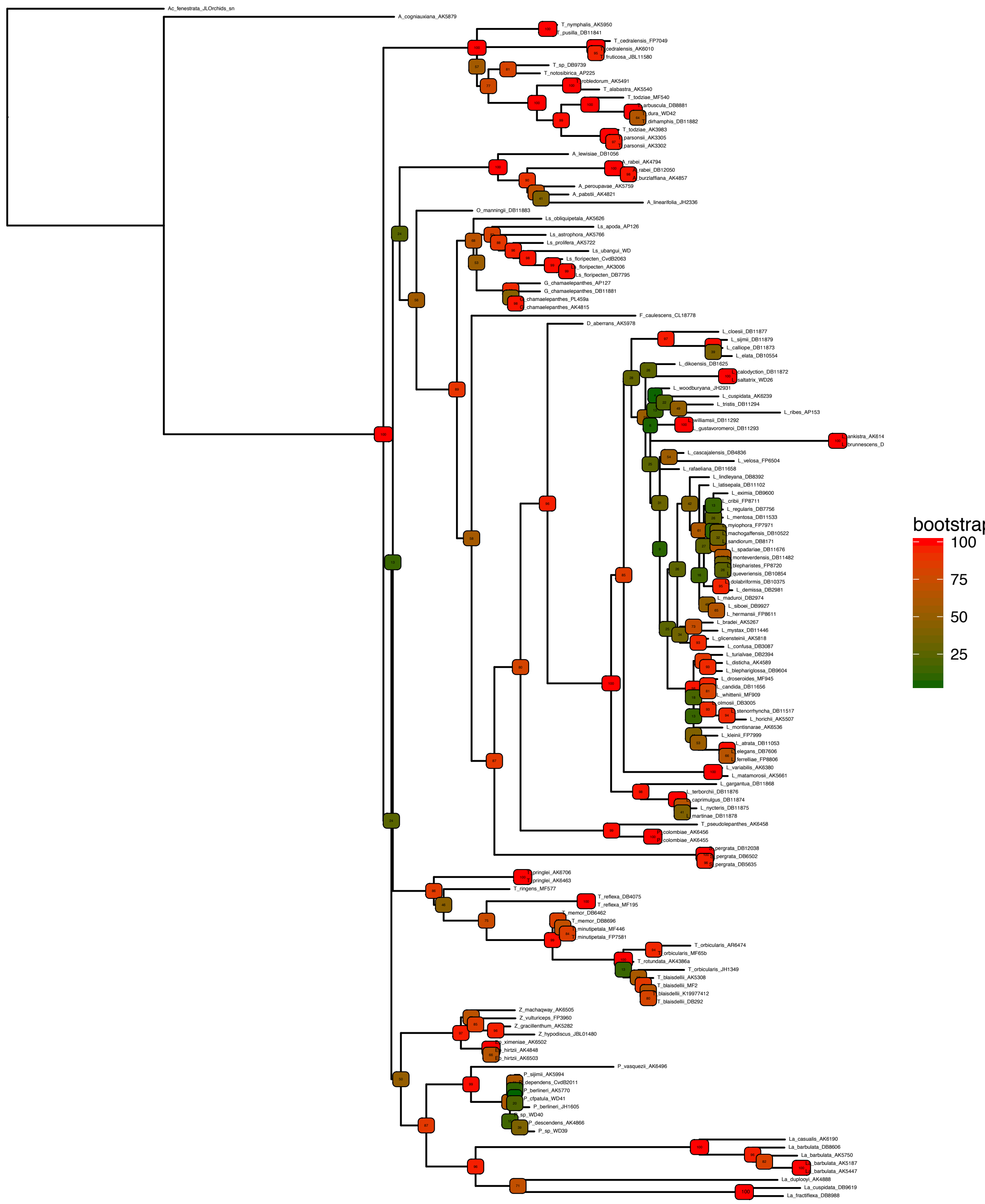

Appendix S5. Co-phylogenetic plot of the topologies of the ML consensus trees inferred from A. ITS dataset B. *mat*K dataset with function *cophylo* of phytools.

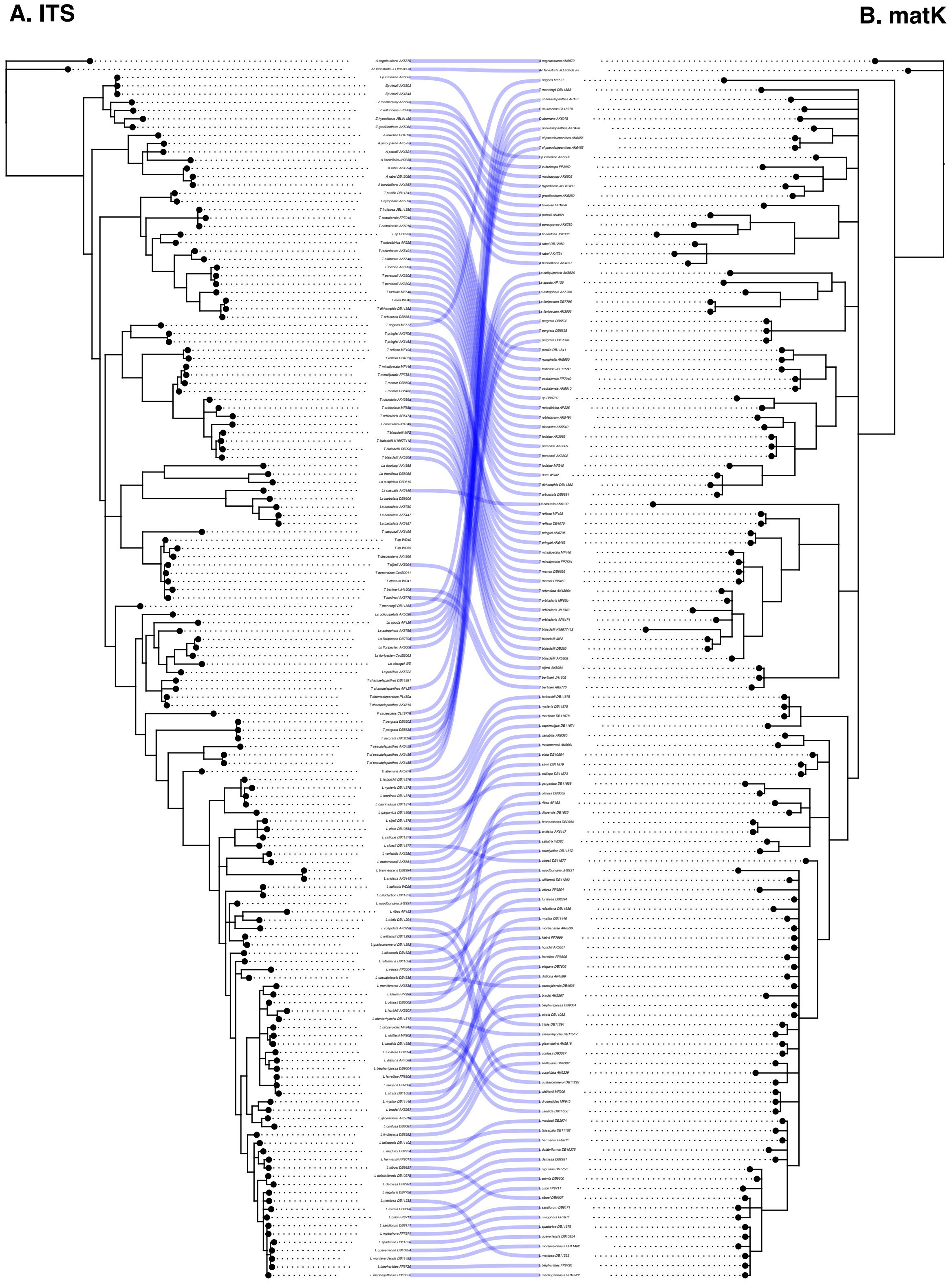
A. ITS
B. matK

Appendix S6. Support values of consensus trees retrieved from the 15 clades recognized as genera belonging to the *Lepanthes* clade and the relationships among them. Values are shown for analyses removing potential outliers identified with PACo and original matrices. Not applicable (N/A) indicates that a clade is represented by only one accession. Not retrieved (NR), indicates clades not obtained in consensus trees.

**Genera**	**Clades**	**Bayesian Inference (PP)**		**Maximum Likelihood (MLB)**		**Maximum Parsimony (MPB)**	
		PACo	No PACo	PACo	No PACo	PACo	No PACo
Lepanthes	A	1.00	1.00	100	100	100	101
Draconanthes	B	N/A	N/A	N/A	N/A	N/A	N/A
Pseudolepanthes	C	1.00	1.00	100	100	100	100
Stellamaris	D	1.00	1.00	100	100	100	100
Frondaria	E	N/A	N/A	N/A	N/A	N/A	N/A
Lepanthopsis	F	0.98	1.00	72	75	100	100
Gravendeelia	G	1.00	1.00	97	98	100	100
Opilionanthe	H	N/A	N/A	N/A	N/A	N/A	N/A
Pendusalpinx	I	1.00	1.00	99	99	100	100
Lankesteriana	J	1.00	1.00	94	94	100	100
Trichosalpinx	K	1.00	1.00	98	61	100	100
Tubella	L	1.00	1.00	100	100	100	100
Anathallis	M	1.00	1.00	100	100	100	100
Zootrophion	N	1.00	1.00	100	100	100	100
**Relashionships among genera**							
Lepanthes + *Draconanthes*	1	1.00	1.00	100	88	100	100
Pseudolepanthes + Clade 1	2	0.96	0.96	78	75	NR	NR
Stellamaris + Clade 2	3	0.98	0.99	80	77	NR	NR
Frondaria + Clade 3	4	0.97	0.96	56	59	100	100
Clade 4 + Clade 6	5	1.00	1.00	92	89	100	100
Lepanthopsis + *Gravendeelia*	6	1.00	1.00	64	75	100	100
Opilionanthe + Clade 5	7	0.94	0.94	58	47	100	NR
Clade 9 + Clade 7	8	0.93	NR	32	NR	100	NR
Trichosalpinx + *Lankesteriana* + *Pendusalpinx*	9	0.96	NR	54	NR	100	NR
Lankesteriana + *Pendusalpinx*	10	1.00	1.00	91	91	100	100
Clade 8 + Clade 12	11	1.00	NR	54	NR	100	NR
Anathallis + *Tubella*	12	0.87	0.68	30	29	100	NR
Clade 11 + Clade 14	13	1.00	1.00	100	NR	100	NR
Zootrophion	14	1.00	1.00	99	99	100	100

Appendix S7. Potential conflicting terminals between nuclear and plastid derived phylogenies from BI and ML respectively as inferred by PACo.

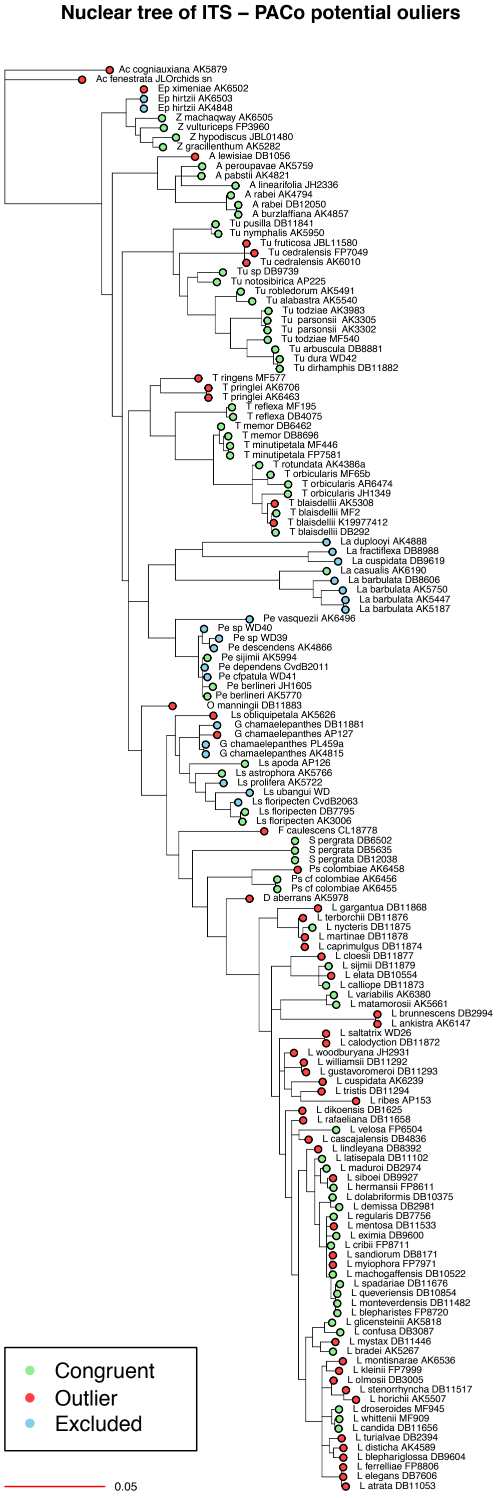

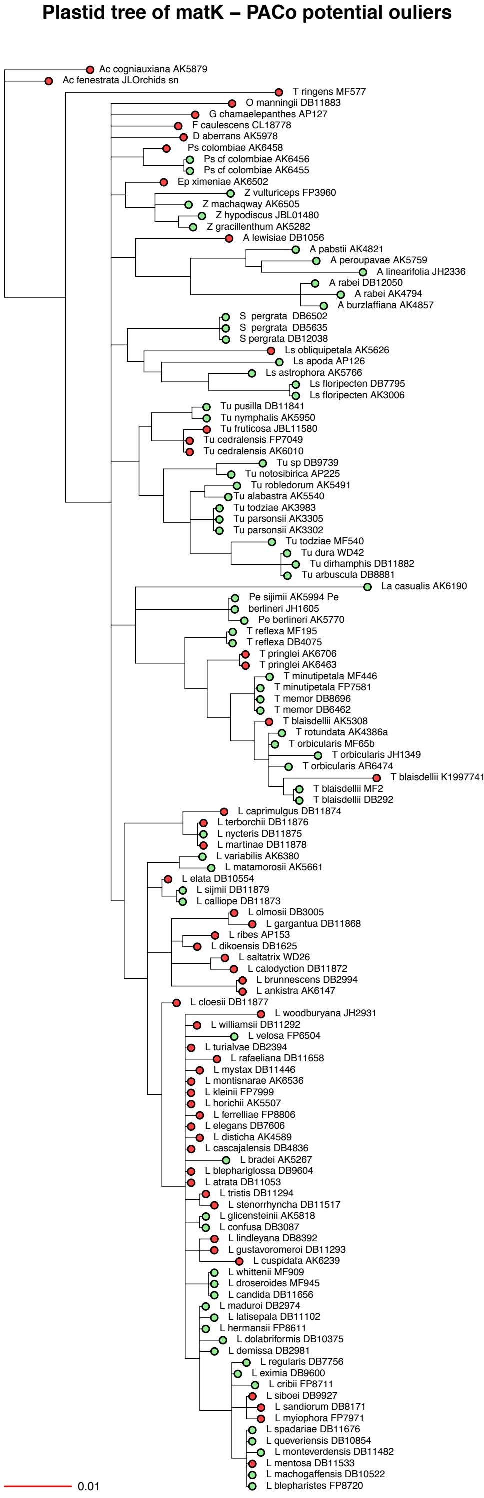

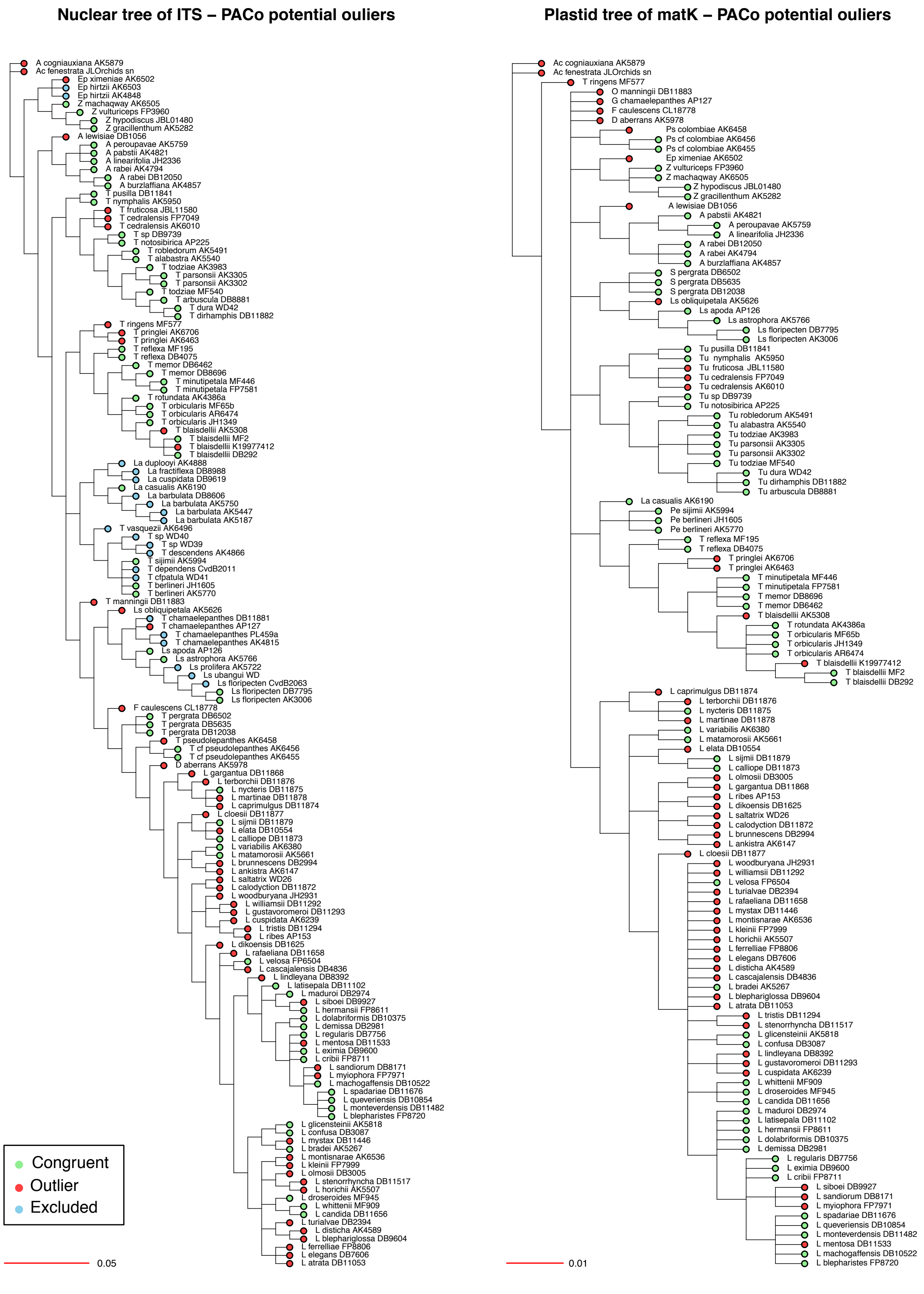
Nuclear tree of ITS – PACo potential ouliers
A cogniauxiana AK5879
Ac fenestrata JLOrchids sn
Ep ximeniae AK6502
Ep hirtzii AK6503
Ep hirtzii AK4848
Z machaqway AK6505
Z vulturiceps FP3960
Z hypodiscus JBL01480
Z gracillenthum AK5282
A lewisiae DB1056
A peroupavae AK5759
A pabstii AK4821
A linearifolia JH2336
A rabei AK4794
A rabei DB12050
A burzlaffiana AK4857
T pusilla DB11841
T nymphalis AK5950
T fruticosa JBL11580
T cedralensis FP7049
T cedralensis AK6010
T sp DB9739
T notosibirica AP225
T robledorum AK5491
T alabastra AK5540
T todziae AK3983
T parsonsii AK3305
T parsonsii AK3302
T todziae MF540
T arbuscula DB8881
T dura WD42
T dirhamphis DB11882
T ringens MF577
T pringlei AK6706
T pringlei AK6463
T reflexa MF195
T reflexa DB4075
T memor DB6462
T memor DB8696
T minutipetala MF446
T minutipetala FP7581
T rotundata AK4386a
T orbicularis MF65b
T orbicularis AR6474
T orbicularis JH1349
T blaisdellii AK5308
T blaisdellii MF2
T blaisdellii K19977412
T blaisdellii DB292
La duplooyi AK4888
La fractiflexa DB8988
La cuspidata DB9619
La casualis AK6190
La barbulata DB8606
La barbulata AK5750
La barbulata AK5447
La barbulata AK5187
T vasquezii AK6496
T sp WD40
T sp WD39
T descendens AK4866
T sijimii AK5994
T dependens CvdB2011
T cfpatula WD41
T berlineri JH1605
T berlineri AK5770
T manningii DB11883
Ls obliquipetala AK5626
T chamaelepanthes DB11881
T chamaelepanthes AP127
T chamaelepanthes PL459a
T chamaelepanthes AK4815
Ls apoda AP126
Ls astrophora AK5766
Ls prolifera AK5722
Ls ubangui WD
Ls floripecten CvdB2063
Ls floripecten DB7795
Ls floripecten AK3006
F caulescens CL18778
T pergrata DB6502
T pergrata DB5635
T pergrata DB12038
T pseudolepanthes AK6458
T cf pseudolepanthes AK6456
T cf pseudolepanthes AK6455
D aberrans AK5978
L gargantua DB11868
L terborchii DB11876
L nycteris DB11875
L martinae DB11878
L caprimulgus DB11874
L cloesii DB11877
L sijmii DB11879
L elata DB10554
L calliope DB11873
L variabilis AK6380
L matamorosii AK5661
L brunnescens DB2994
L ankistra AK6147
L saltatrix WD26
L calodyction DB11872
L woodburyana JH2931
L williamsii DB11292
L gustavoromeroi DB11293
L cuspidata AK6239
L tristis DB11294
L ribes AP153
L dikoensis DB1625
L rafaeliana DB11658
L velosa FP6504
L cascajalensis DB4836
L lindleyana DB8392
L latisepala DB11102
L maduroi DB2974
L siboei DB9927
L hermansii FP8611
L dolabriformis DB10375
L demissa DB2981
L regularis DB7756
L mentosa DB11533
L eximia DB9600
L cribii FP8711
L sandiorum DB8171
L myiophora FP7971
L machogaffensis DB10522
L spadariae DB11676
L queveriensis DB10854
L monteverdensis DB11482
L blepharistes FP8720
L glicensteinii AK5818
L confusa DB3087
L mystax DB11446
L bradei AK5267
L montisnarae AK6536
L kleinii FP7999
L olmosii DB3005
L stenorrhyncha DB11517
L horichii AK5507
L droseroides MF945
L whittenii MF909
L candida DB11656
L turialvae DB2394
L disticha AK4589
L blephariglossa DB9604
L ferrelliae FP8806
L elegans DB7606
L atrata DB11053
Congruent
Outlier
Excluded
0.05
Plastid tree of matK – PACo potential ouliers
Ac cogniauxiana AK5879
Ac fenestrata JLOrchids sn
T ringens MF577
O manningii DB11883
G chamaelepanthes AP127
F caulescens CL18778
D aberrans AK5978
Ps colombiae AK6458
Ps cf colombiae AK6456
Ps cf colombiae AK6455
Ep ximeniae AK6502
Z vulturiceps FP3960
Z machaqway AK6505
Z hypodiscus JBL01480
Z gracillenthum AK5282
A lewisiae DB1056
A pabstii AK4821
A peroupavae AK5759
A linearifolia JH2336
A rabei DB12050
A rabei AK4794
A burzlaffiana AK4857
S pergrata DB6502
S pergrata DB5635
S pergrata DB12038
Ls obliquipetala AK5626
Ls apoda AP126
Ls astrophora AK5766
Ls floripecten DB7795
Ls floripecten AK3006
Tu pusilla DB11841
Tu nymphalis AK5950
Tu fruticosa JBL11580
Tu cedralensis FP7049
Tu cedralensis AK6010
Tu sp DB9739
Tu notosibirica AP225
Tu robledorum AK5491
Tu alabastra AK5540
Tu todziae AK3983
Tu parsonsii AK3305
Tu parsonsii AK3302
Tu todziae MF540
Tu dura WD42
Tu dirhamphis DB11882
Tu arbuscula DB8881
La casualis AK6190
Pe sijimii AK5994
Pe berlineri JH1605
Pe berlineri AK5770
T reflexa MF195
T reflexa DB4075
T pringlei AK6706
T pringlei AK6463
T minutipetala MF446
T minutipetala FP7581
T memor DB8696
T memor DB6462
T blaisdellii AK5308
T rotundata AK4386a
T orbicularis MF65b
T orbicularis JH1349
T orbicularis AR6474
T blaisdellii K19977412
T blaisdellii MF2
T blaisdellii DB292
L caprimulgus DB11874
L terborchii DB11876
L nycteris DB11875
L martinae DB11878
L variabilis AK6380
L matamorosii AK5661
L elata DB10554
L sijmii DB11879
L calliope DB11873
L olmosii DB3005
L gargantua DB11868
L ribes AP153
L dikoensis DB1625
L saltatrix WD26
L calodyction DB11872
L brunnescens DB2994
L ankistra AK6147
L cloesii DB11877
L woodburyana JH2931
L williamsii DB11292
L velosa FP6504
L turialvae DB2394
L rafaeliana DB11658
L mystax DB11446
L montisnarae AK6536
L kleinii FP7999
L horichii AK5507
L ferrelliae FP8806
L elegans DB7606
L disticha AK4589
L cascajalensis DB4836
L bradei AK5267
L blephariglossa DB9604
L atrata DB11053
L tristis DB11294
L stenorrhyncha DB11517
L glicensteinii AK5818
L confusa DB3087
L lindleyana DB8392
L gustavoromeroi DB11293
L cuspidata AK6239
L whittenii MF909
L droseroides MF945
L candida DB11656
L maduroi DB2974
L latisepala DB11102
L hermansii FP8611
L dolabriformis DB10375
L demissa DB2981
L regularis DB7756
L eximia DB9600
L cribii FP8711
L siboei DB9927
L sandiorum DB8171
L myiophora FP7971
L spadariae DB11676
L queveriensis DB10854
L monteverdensis DB11482
L mentosa DB11533
L machogaffensis DB10522
L blepharistes FP8720
0.01

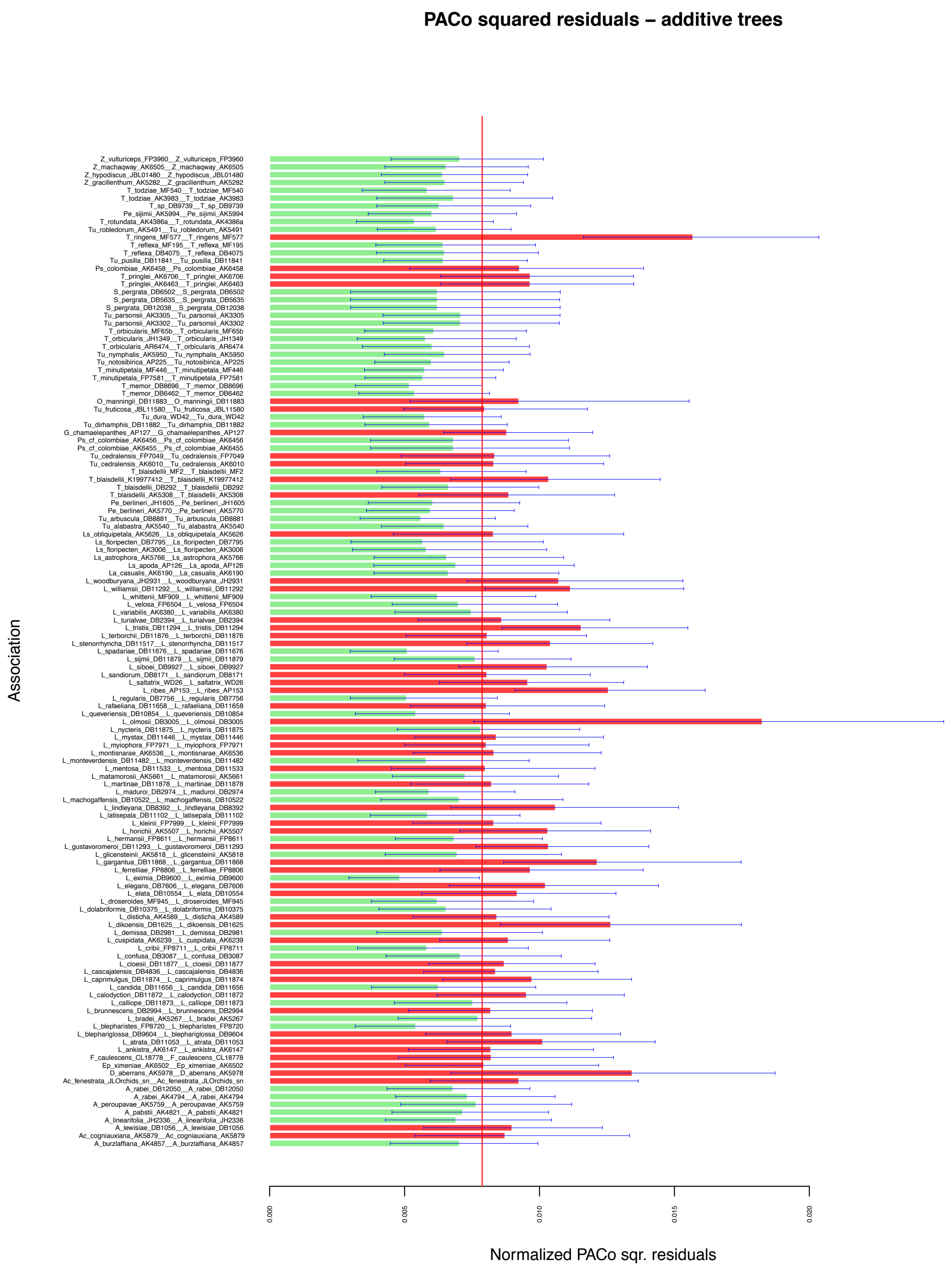

PACo squared residuals – additive trees
Association
Normalized PACo sqr. residuals
0.000
0.005
0.010
0.015
0.020

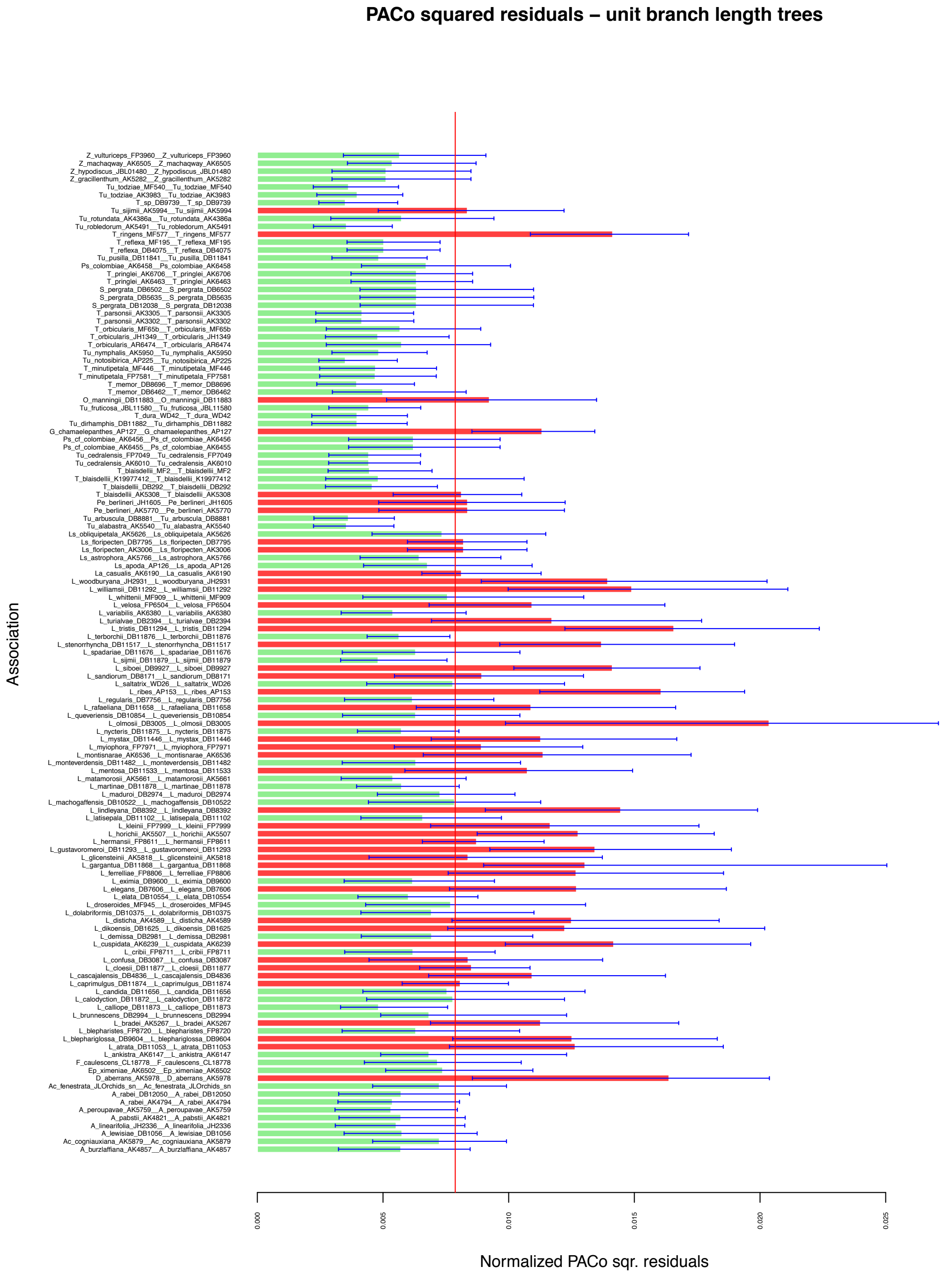
PACo squared residuals – unit branch length trees
Association
Normalized PACo sqr. residuals
0.000
0.005
0.010
0.015
0.020
0.025

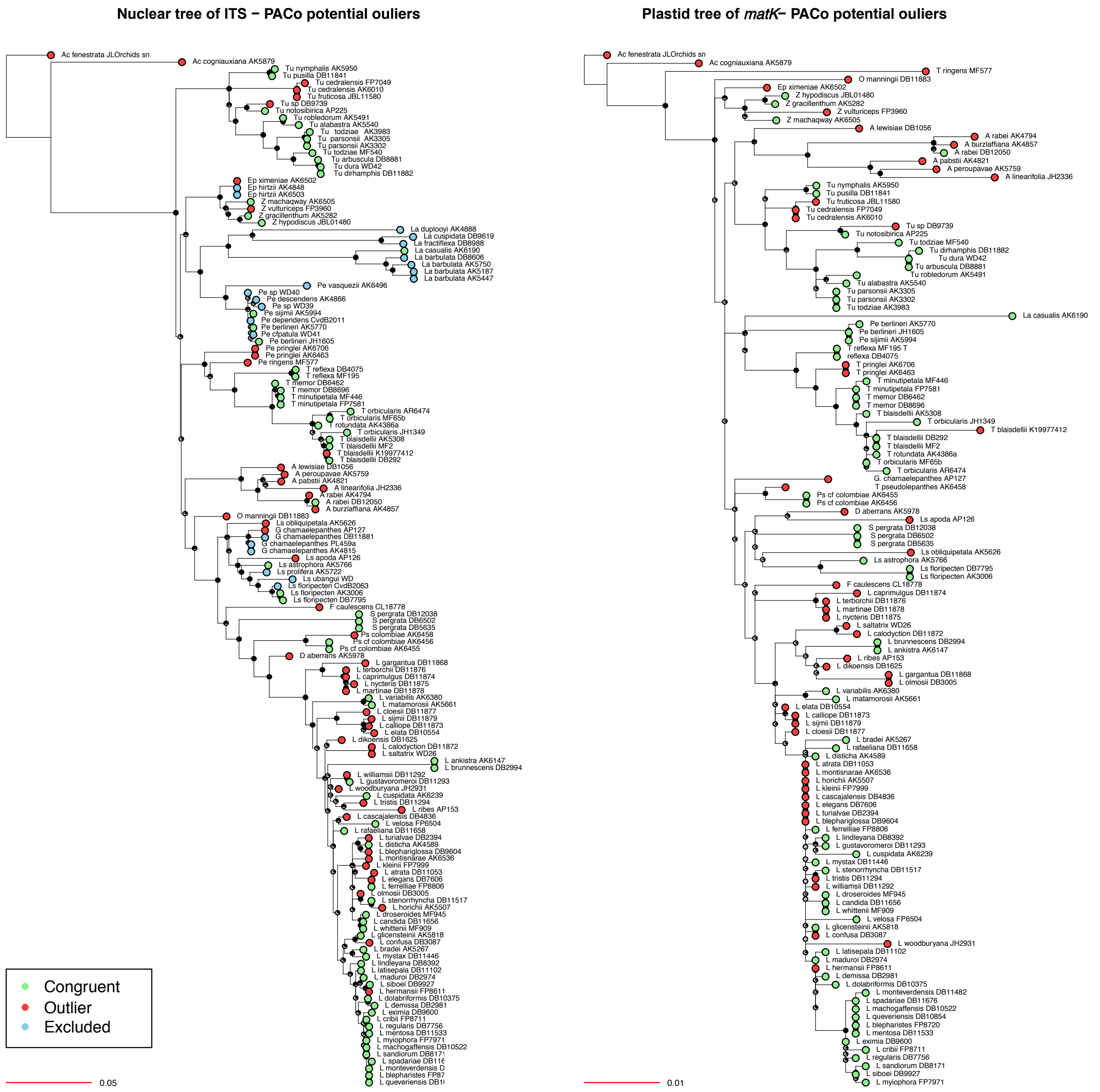
Nuclear tree of ITS − PACo potential ouliers
Plastid tree of matK− PACo potential ouliers
Ac fenestrata JLOrchids sn
Ac cogniauxiana AK5879
Tu nymphalis AK5950
Tu pusilla DB11841
Tu cedralensis FP7049
Tu cedralensis AK6010
Tu fruticosa JBL11580
Tu sp DB9739
Tu notosibirica AP225
Tu robledorum AK5491
Tu alabastra AK5540
Tu todziae AK3983
Tu parsonsii AK3305
Tu parsonsii AK3302
Tu todziae MF540
Tu arbuscula DB8881
Tu dura WD42
Tu dirhamphis DB11882
Ep ximeniae AK6502
Ep hirtzii AK4848
Ep hirtzii AK6503
Z machaqway AK6505
Z vulturiceps FP3960
Z gracillenthum AK5282
Z hypodiscus JBL01480
La duplooyi AK4888
La cuspidata DB9619
La fractiflexa DB8988
La casualis AK6190
La barbulata DB8606
La barbulata AK5750
La barbulata AK5187
La barbulata AK5447
Pe vasquezii AK6496
Pe sp WD40
Pe descendens AK4866
Pe sp WD39
Pe sijimii AK5994
Pe dependens CvdB2011
Pe berlineri AK5770
Pe cfpatula WD41
Pe berlineri JH1605
Pe pringlei AK6706
Pe pringlei AK6463
Pe ringens MF577
T reflexa DB4075
T reflexa MF195
T memor DB6462
T memor DB8696
T minutipetala MF446
T minutipetala FP7581
T orbicularis AR6474
T orbicularis MF65b
T rotundata AK4386a
T orbicularis JH1349
T blaisdellii AK5308
T blaisdellii MF2
T blaisdellii K19977412
T blaisdellii DB292
A lewisiae DB1056
A peroupavae AK5759
A pabstii AK4821
A linearifolia JH2336
A rabei AK4794
A rabei DB12050
A burzlaffiana AK4857
O manningii DB11883
Ls obliquipetala AK5626
G chamaelepanthes AP127
G chamaelepanthes DB11881
G chamaelepanthes PL459a
G chamaelepanthes AK4815
Ls apoda AP126
Ls astrophora AK5766
Ls prolifera AK5722
Ls ubangui WD
Ls floripecten CvdB2063
Ls floripecten AK3006
Ls floripecten DB7795
F caulescens CL18778
S pergrata DB12038
S pergrata DB6502
S pergrata DB5635
Ps colombiae AK6458
Ps cf colombiae AK6456
Ps cf colombiae AK6455
D aberrans AK5978
L gargantua DB11868
L terborchii DB11876
L caprimulgus DB11874
L nycteris DB11875
L martinae DB11878
L variabilis AK6380
L matamorosii AK5661
L cloesii DB11877
L sijmii DB11879
L calliope DB11873
L elata DB10554
L dikoensis DB1625
L calodyction DB11872
L saltatrix WD26
L ankistra AK6147
L brunnescens DB2994
L williamsii DB11292
L gustavoromeroi DB11293
L woodburyana JH2931
L cuspidata AK6239
L tristis DB11294
L ribes AP153
L cascajalensis DB4836
L velosa FP6504
L rafaeliana DB11658
L turialvae DB2394
L disticha AK4589
L blephariglossa DB9604
L montisnarae AK6536
L kleinii FP7999
L atrata DB11053
L elegans DB7606
L ferrelliae FP8806
L olmosii DB3005
L stenorrhyncha DB11517
L horichii AK5507
L droseroides MF945
L candida DB11656
L whittenii MF909
L glicensteinii AK5818
L confusa DB3087
L bradei AK5267
L mystax DB11446
L lindleyana DB8392
L latisepala DB11102
L maduroi DB2974
L siboei DB9927
L hermansii FP8611
L dolabriformis DB10375
L demissa DB2981
L eximia DB9600
L cribii FP8711
L regularis DB7756
L mentosa DB11533
L myiophora FP7971
L machogaffensis DB10522
L sandiorum DB8171
L spadariae DB116
L monteverdensis D
L blepharistes FP87
L queveriensis DB1
Congruent
Outlier
Excluded
0.05
Ac fenestrata JLOrchids sn
Ac cogniauxiana AK5879
T ringens MF577
O manningii DB11883
Ep ximeniae AK6502
Z hypodiscus JBL01480
Z gracillenthum AK5282
Z vulturiceps FP3960
Z machaqway AK6505
A lewisiae DB1056
A rabei AK4794
A burzlaffiana AK4857
A rabei DB12050
A pabstii AK4821
A peroupavae AK5759
A linearifolia JH2336
Tu nymphalis AK5950
Tu pusilla DB11841
Tu fruticosa JBL11580
Tu cedralensis FP7049
Tu cedralensis AK6010
Tu sp DB9739
Tu notosibirica AP225
Tu todziae MF540
Tu dirhamphis DB11882
Tu dura WD42
Tu arbuscula DB8881
Tu robledorum AK5491
Tu alabastra AK5540
Tu parsonsii AK3305
Tu parsonsii AK3302
Tu todziae AK3983
La casualis AK6190
Pe berlineri AK5770
Pe berlineri JH1605
Pe sijimii AK5994
T reflexa MF195 T
reflexa DB4075
T pringlei AK6706
T pringlei AK6463
T minutipetala MF446
T minutipetala FP7581
T memor DB6462
T memor DB8696
T blaisdellii AK5308
T orbicularis JH1349
T blaisdellii K19977412
T blaisdellii DB292
T blaisdellii MF2
T rotundata AK4386a
T orbicularis MF65b
T orbicularis AR6474
G. chamaelepanthes AP127
T pseudolepanthes AK6458
Ps cf colombiae AK6455
Ps cf colombiae AK6456
D aberrans AK5978
Ls apoda AP126
S pergrata DB12038
S pergrata DB6502
S pergrata DB5635
Ls obliquipetala AK5626
Ls astrophora AK5766
Ls floripecten DB7795
Ls floripecten AK3006
F caulescens CL18778
L caprimulgus DB11874
L terborchii DB11876
L martinae DB11878
L nycteris DB11875
L saltatrix WD26
L calodyction DB11872
L brunnescens DB2994
L ankistra AK6147
L ribes AP153
L dikoensis DB1625
L gargantua DB11868
L olmosii DB3005
L variabilis AK6380
L matamorosii AK5661
L elata DB10554
L calliope DB11873
L sijmii DB11879
L cloesii DB11877
L bradei AK5267
L rafaeliana DB11658
L disticha AK4589
L atrata DB11053
L montisnarae AK6536
L horichii AK5507
L kleinii FP7999
L cascajalensis DB4836
L elegans DB7606
L turialvae DB2394
L blephariglossa DB9604
L ferrelliae FP8806
L lindleyana DB8392
L gustavoromeroi DB11293
L cuspidata AK6239
L mystax DB11446
L stenorrhyncha DB11517
L tristis DB11294
L williamsii DB11292
L droseroides MF945
L candida DB11656
L whittenii MF909
L velosa FP6504
L glicensteinii AK5818
L confusa DB3087
L woodburyana JH2931
L latisepala DB11102
L maduroi DB2974
L hermansii FP8611
L demissa DB2981
L dolabriformis DB10375
L monteverdensis DB11482
L spadariae DB11676
L machogaffensis DB10522
L queveriensis DB10854
L blepharistes FP8720
L mentosa DB11533
L eximia DB9600
L cribii FP8711
L regularis DB7756
L sandiorum DB8171
L siboei DB9927
L myiophora FP7971
0.01

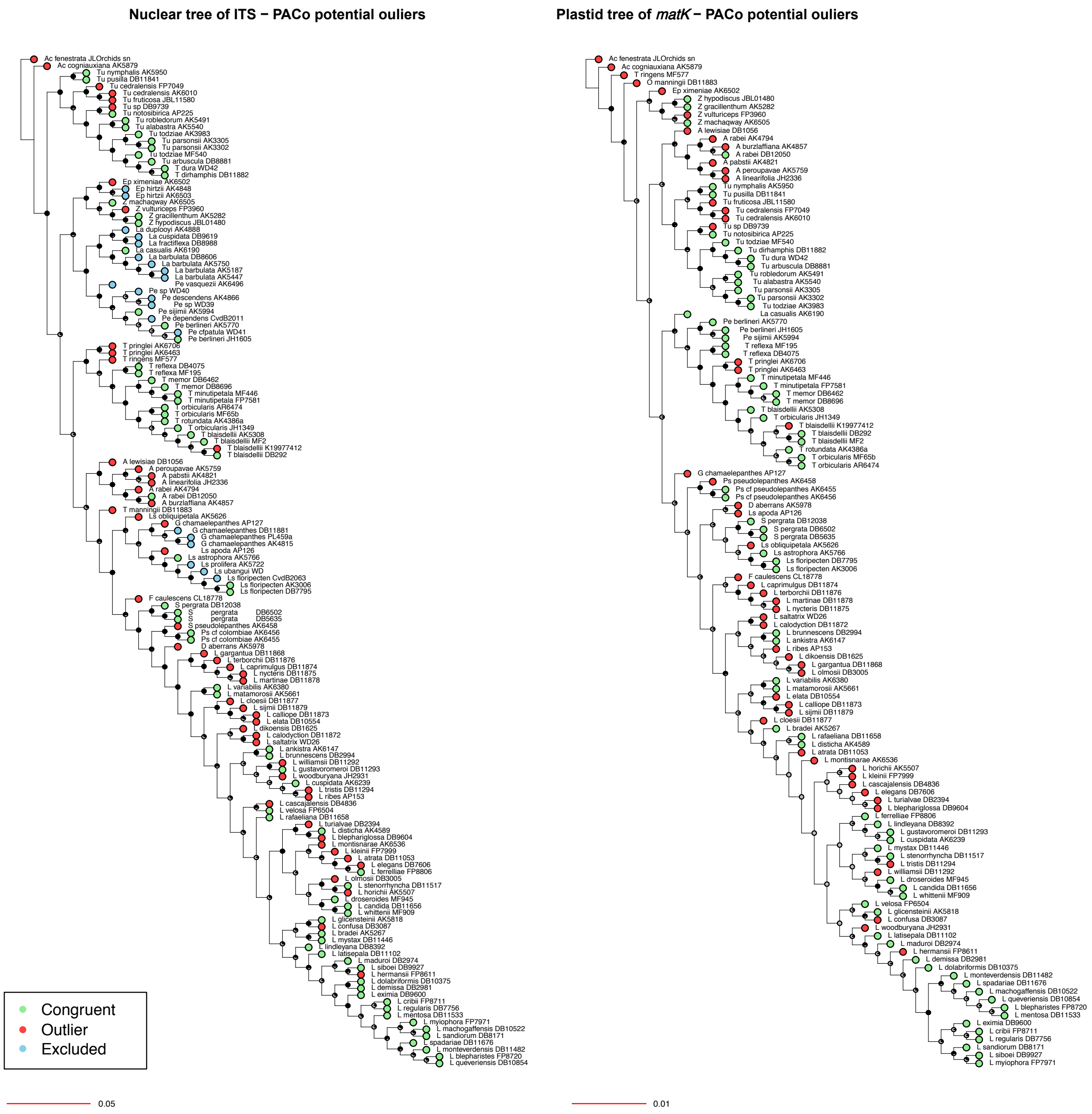
Nuclear tree of ITS − PACo potential ouliers
Plastid tree of matK − PACo potential ouliers
Congruent
Outlier
Excluded
0.05
0.01

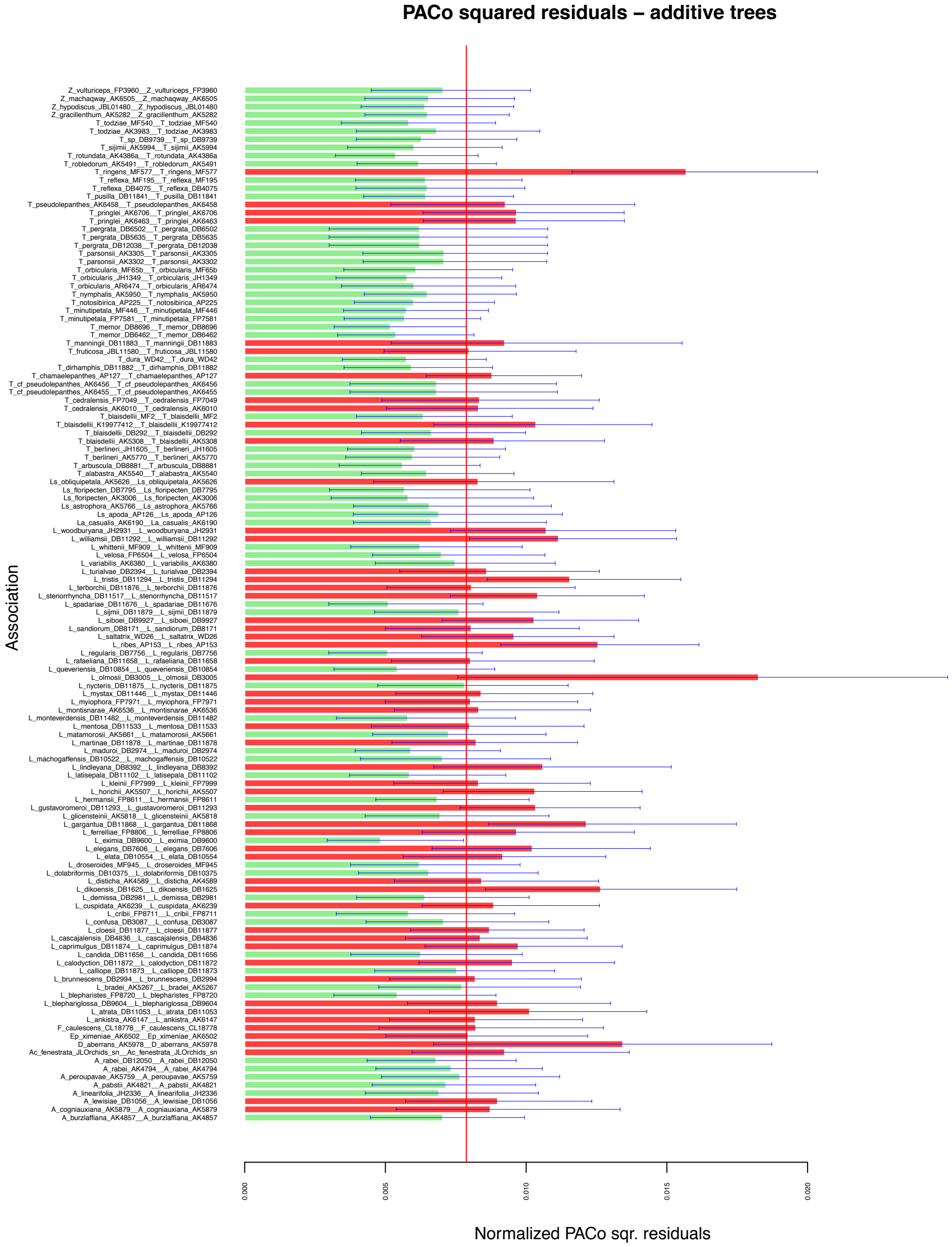
PACo squared residuals – additive trees
Z_vulturiceps_FP3960__Z_vulturiceps_FP3960
Z_machaqway_AK6505__Z_machaqway_AK6505
Z_hypodiscus_JBL01480__Z_hypodiscus_JBL01480
Z_gracillenthum_AK5282__Z_gracillenthum_AK5282
T_todziae_MF540__T_todziae_MF540
T_todziae_AK3983__T_todziae_AK3983
T_sp_DB9739__T_sp_DB9739
T_sijimii_AK5994__T_sijimii_AK5994
T_rotundata_AK4386a__T_rotundata_AK4386a
T_robledorum_AK5491__T_robledorum_AK5491
T_ringens_MF577__T_ringens_MF577
T_reflexa_MF195__T_reflexa_MF195
T_reflexa_DB4075__T_reflexa_DB4075
T_pusilla_DB11841__T_pusilla_DB11841
T_pseudolepanthes_AK6458__T_pseudolepanthes_AK6458
T_pringlei_AK6706__T_pringlei_AK6706
T_pringlei_AK6463__T_pringlei_AK6463
T_pergrata_DB6502__T_pergrata_DB6502
T_pergrata_DB5635__T_pergrata_DB5635
T_pergrata_DB12038__T_pergrata_DB12038
T_parsonsii_AK3305__T_parsonsii_AK3305
T_parsonsii_AK3302__T_parsonsii_AK3302
T_orbicularis_MF65b__T_orbicularis_MF65b
T_orbicularis_JH1349__T_orbicularis_JH1349
T_orbicularis_AR6474__T_orbicularis_AR6474
T_nymphalis_AK5950__T_nymphalis_AK5950
T_notosibirica_AP225__T_notosibirica_AP225
T_minutipetala_MF446__T_minutipetala_MF446
T_minutipetala_FP7581__T_minutipetala_FP7581
T_memor_DB8696__T_memor_DB8696
T_memor_DB6462__T_memor_DB6462
T_manningii_DB11883__T_manningii_DB11883
T_fruticosa_JBL11580__T_fruticosa_JBL11580
T_dura_WD42__T_dura_WD42
T_dirhamphis_DB11882__T_dirhamphis_DB11882
T_chamaelepanthes_AP127__T_chamaelepanthes_AP127
T_cf_pseudolepanthes_AK6456__T_cf_pseudolepanthes_AK6456
T_cf_pseudolepanthes_AK6455__T_cf_pseudolepanthes_AK6455
T_cedralensis_FP7049__T_cedralensis_FP7049
T_cedralensis_AK6010__T_cedralensis_AK6010
T_blaisdellii_MF2__T_blaisdellii_MF2
T_blaisdellii_K19977412__T_blaisdellii_K19977412
T_blaisdellii_DB292__T_blaisdellii_DB292
T_blaisdellii_AK5308__T_blaisdellii_AK5308
T_berlineri_JH1605__T_berlineri_JH1605
T_berlineri_AK5770__T_berlineri_AK5770
T_arbuscula_DB8881__T_arbuscula_DB8881
T_alabastra_AK5540__T_alabastra_AK5540
Ls_obliquipetala_AK5626__Ls_obliquipetala_AK5626
Ls_floripecten_DB7795__Ls_floripecten_DB7795
Ls_floripecten_AK3006__Ls_floripecten_AK3006
Ls_astrophora_AK5766__Ls_astrophora_AK5766
Ls_apoda_AP126__Ls_apoda_AP126
La_casualis_AK6190__La_casualis_AK6190
L_woodburyana_JH2931__L_woodburyana_JH2931
L_williamsii_DB11292__L_williamsii_DB11292
L_whittenii_MF909__L_whittenii_MF909
L_velosa_FP6504__L_velosa_FP6504
L_variabilis_AK6380__L_variabilis_AK6380
L_turialvae_DB2394__L_turialvae_DB2394
L_tristis_DB11294__L_tristis_DB11294
L_terborchii_DB11876__L_terborchii_DB11876
L_stenorrhyncha_DB11517__L_stenorrhyncha_DB11517
L_spadariae_DB11676__L_spadariae_DB11676
L_sijmii_DB11879__L_sijmii_DB11879
L_siboei_DB9927__L_siboei_DB9927
L_sandiorum_DB8171__L_sandiorum_DB8171
L_saltatrix_WD26__L_saltatrix_WD26
L_ribes_AP153__L_ribes_AP153
L_regularis_DB7756__L_regularis_DB7756
L_rafaeliana_DB11658__L_rafaeliana_DB11658
L_queveriensis_DB10854__L_queveriensis_DB10854
L_olmosii_DB3005__L_olmosii_DB3005
L_nycteris_DB11875__L_nycteris_DB11875
L_mystax_DB11446__L_mystax_DB11446
L_myiophora_FP7971__L_myiophora_FP7971
L_montisnarae_AK6536__L_montisnarae_AK6536
L_monteverdensis_DB11482__L_monteverdensis_DB11482
L_mentosa_DB11533__L_mentosa_DB11533
L_matamorosii_AK5661__L_matamorosii_AK5661
L_martinae_DB11878__L_martinae_DB11878
L_maduroi_DB2974__L_maduroi_DB2974
L_machogaffensis_DB10522__L_machogaffensis_DB10522
L_lindleyana_DB8392__L_lindleyana_DB8392
L_latisepala_DB11102__L_latisepala_DB11102
L_kleinii_FP7999__L_kleinii_FP7999
L_horichii_AK5507__L_horichii_AK5507
L_hermansii_FP8611__L_hermansii_FP8611
L_gustavoromeroi_DB11293__L_gustavoromeroi_DB11293
L_glicensteinii_AK5818__L_glicensteinii_AK5818
L_gargantua_DB11868__L_gargantua_DB11868
L_ferrelliae_FP8806__L_ferrelliae_FP8806
L_eximia_DB9600__L_eximia_DB9600
L_elegans_DB7606__L_elegans_DB7606
L_elata_DB10554__L_elata_DB10554
L_droseroides_MF945__L_droseroides_MF945
L_dolabriformis_DB10375__L_dolabriformis_DB10375
L_disticha_AK4589__L_disticha_AK4589
L_dikoensis_DB1625__L_dikoensis_DB1625
L_demissa_DB2981__L_demissa_DB2981
L_cuspidata_AK6239__L_cuspidata_AK6239
L_cribii_FP8711__L_cribii_FP8711
L_confusa_DB3087__L_confusa_DB3087
L_cloesii_DB11877__L_cloesii_DB11877
L_cascajalensis_DB4836__L_cascajalensis_DB4836
L_caprimulgus_DB11874__L_caprimulgus_DB11874
L_candida_DB11656__L_candida_DB11656
L_calodyction_DB11872__L_calodyction_DB11872
L_calliope_DB11873__L_calliope_DB11873
L_brunnescens_DB2994__L_brunnescens_DB2994
L_bradei_AK5267__L_bradei_AK5267
L_blepharistes_FP8720__L_blepharistes_FP8720
L_blephariglossa_DB9604__L_blephariglossa_DB9604
L_atrata_DB11053__L_atrata_DB11053
L_ankistra_AK6147__L_ankistra_AK6147
F_caulescens_CL18778__F_caulescens_CL18778
Ep_ximeniae_AK6502__Ep_ximeniae_AK6502
D_aberrans_AK5978__D_aberrans_AK5978
Ac_fenestrata_JLOrchids_sn__Ac_fenestrata_JLOrchids_sn
A_rabei_DB12050__A_rabei_DB12050
A_rabei_AK4794__A_rabei_AK4794
A_peroupavae_AK5759__A_peroupavae_AK5759
A_pabstii_AK4821__A_pabstii_AK4821
A_linearifolia_JH2336__A_linearifolia_JH2336
A_lewisiae_DB1056__A_lewisiae_DB1056
A_cogniauxiana_AK5879__A_cogniauxiana_AK5879
A_burzlaffiana_AK4857__A_burzlaffiana_AK4857
Association
0.000
0.005
0.010
0.015
0.020
Normalized PACo sqr. residuals

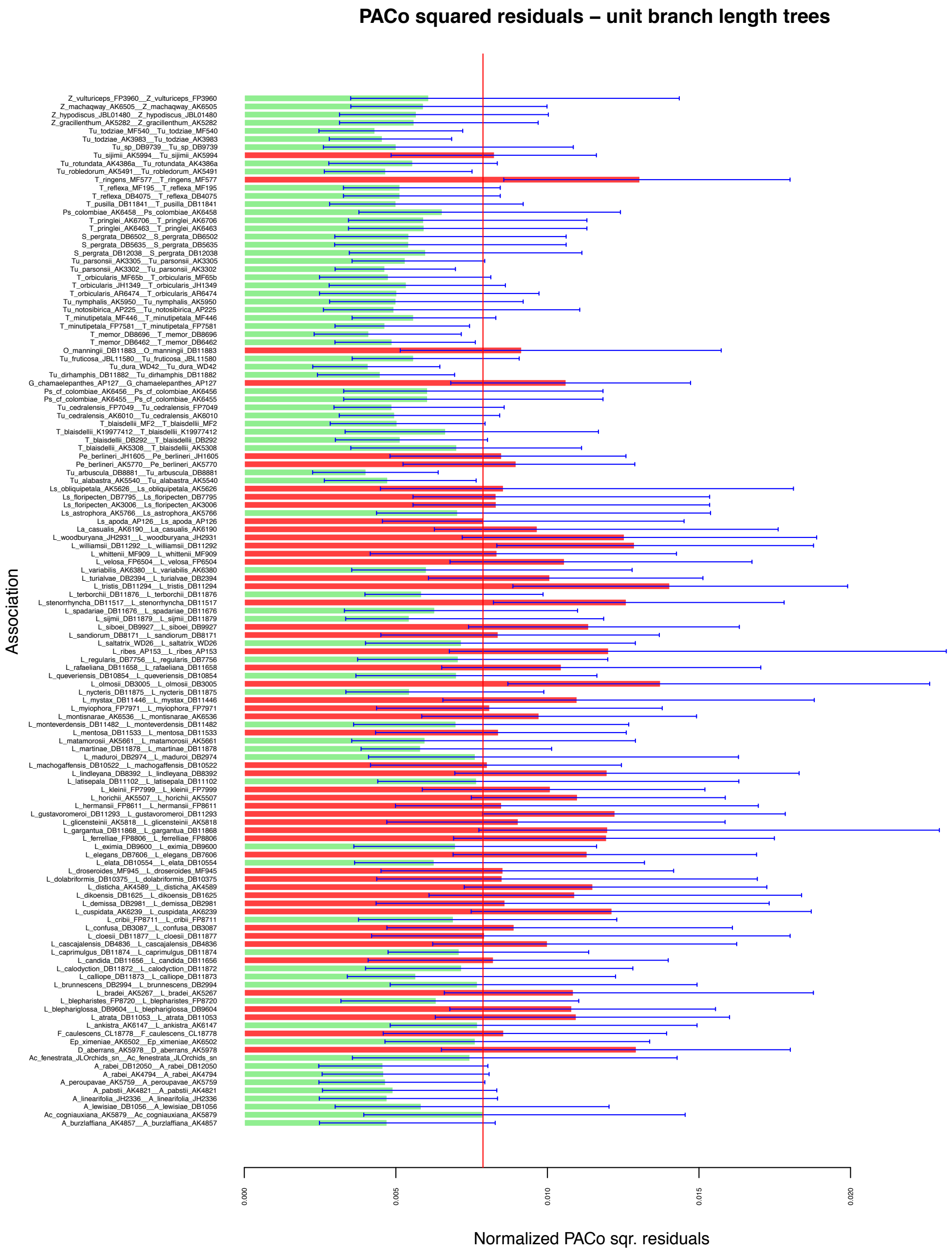
PACo squared residuals – unit branch length trees
Association
Z_vulturiceps_FP3960__Z_vulturiceps_FP3960
Z_machaqway_AK6505__Z_machaqway_AK6505
Z_hypodiscus_JBL01480__Z_hypodiscus_JBL01480
Z_gracillenthum_AK5282__Z_gracillenthum_AK5282
Tu_todziae_MF540__Tu_todziae_MF540
Tu_todziae_AK3983__Tu_todziae_AK3983
Tu_sp_DB9739__Tu_sp_DB9739
Tu_sijimii_AK5994__Tu_sijimii_AK5994
Tu_rotundata_AK4386a__Tu_rotundata_AK4386a
Tu_robledorum_AK5491__Tu_robledorum_AK5491
T_ringens_MF577__T_ringens_MF577
T_reflexa_MF195__T_reflexa_MF195
T_reflexa_DB4075__T_reflexa_DB4075
T_pusilla_DB11841__T_pusilla_DB11841
Ps_colombiae_AK6458__Ps_colombiae_AK6458
T_pringlei_AK6706__T_pringlei_AK6706
T_pringlei_AK6463__T_pringlei_AK6463
S_pergrata_DB6502__S_pergrata_DB6502
S_pergrata_DB5635__S_pergrata_DB5635
S_pergrata_DB12038__S_pergrata_DB12038
Tu_parsonsii_AK3305__Tu_parsonsii_AK3305
Tu_parsonsii_AK3302__Tu_parsonsii_AK3302
T_orbicularis_MF65b__T_orbicularis_MF65b
T_orbicularis_JH1349__T_orbicularis_JH1349
T_orbicularis_AR6474__T_orbicularis_AR6474
Tu_nymphalis_AK5950__Tu_nymphalis_AK5950
Tu_notosibirica_AP225__Tu_notosibirica_AP225
T_minutipetala_MF446__T_minutipetala_MF446
T_minutipetala_FP7581__T_minutipetala_FP7581
T_memor_DB8696__T_memor_DB8696
T_memor_DB6462__T_memor_DB6462
O_manningii_DB11883__O_manningii_DB11883
Tu_fruticosa_JBL11580__Tu_fruticosa_JBL11580
Tu_dura_WD42__Tu_dura_WD42
Tu_dirhamphis_DB11882__Tu_dirhamphis_DB11882
G_chamaelepanthes_AP127__G_chamaelepanthes_AP127
Ps_cf_colombiae_AK6456__Ps_cf_colombiae_AK6456
Ps_cf_colombiae_AK6455__Ps_cf_colombiae_AK6455
Tu_cedralensis_FP7049__Tu_cedralensis_FP7049
Tu_cedralensis_AK6010__Tu_cedralensis_AK6010
T_blaisdellii_MF2__T_blaisdellii_MF2
T_blaisdellii_K19977412__T_blaisdellii_K19977412
T_blaisdellii_DB292__T_blaisdellii_DB292
T_blaisdellii_AK5308__T_blaisdellii_AK5308
Pe_berlineri_JH1605__Pe_berlineri_JH1605
Pe_berlineri_AK5770__Pe_berlineri_AK5770
Tu_arbuscula_DB8881__Tu_arbuscula_DB8881
Tu_alabastra_AK5540__Tu_alabastra_AK5540
Ls_obliquipetala_AK5626__Ls_obliquipetala_AK5626
Ls_floripecten_DB7795__Ls_floripecten_DB7795
Ls_floripecten_AK3006__Ls_floripecten_AK3006
Ls_astrophora_AK5766__Ls_astrophora_AK5766
Ls_apoda_AP126__Ls_apoda_AP126
La_casualis_AK6190__La_casualis_AK6190
L_woodburyana_JH2931__L_woodburyana_JH2931
L_williamsii_DB11292__L_williamsii_DB11292
L_whittenii_MF909__L_whittenii_MF909
L_velosa_FP6504__L_velosa_FP6504
L_variabilis_AK6380__L_variabilis_AK6380
L_turialvae_DB2394__L_turialvae_DB2394
L_tristis_DB11294__L_tristis_DB11294
L_terborchii_DB11876__L_terborchii_DB11876
L_stenorrhyncha_DB11517__L_stenorrhyncha_DB11517
L_spadariae_DB11676__L_spadariae_DB11676
L_sijmii_DB11879__L_sijmii_DB11879
L_siboei_DB9927__L_siboei_DB9927
L_sandiorum_DB8171__L_sandiorum_DB8171
L_saltatrix_WD26__L_saltatrix_WD26
L_ribes_AP153__L_ribes_AP153
L_regularis_DB7756__L_regularis_DB7756
L_rafaeliana_DB11658__L_rafaeliana_DB11658
L_queveriensis_DB10854__L_queveriensis_DB10854
L_olmosii_DB3005__L_olmosii_DB3005
L_nycteris_DB11875__L_nycteris_DB11875
L_mystax_DB11446__L_mystax_DB11446
L_myiophora_FP7971__L_myiophora_FP7971
L_montisnarae_AK6536__L_montisnarae_AK6536
L_monteverdensis_DB11482__L_monteverdensis_DB11482
L_mentosa_DB11533__L_mentosa_DB11533
L_matamorosii_AK5661__L_matamorosii_AK5661
L_martinae_DB11878__L_martinae_DB11878
L_maduroi_DB2974__L_maduroi_DB2974
L_machogaffensis_DB10522__L_machogaffensis_DB10522
L_lindleyana_DB8392__L_lindleyana_DB8392
L_latisepala_DB11102__L_latisepala_DB11102
L_kleinii_FP7999__L_kleinii_FP7999
L_horichii_AK5507__L_horichii_AK5507
L_hermansii_FP8611__L_hermansii_FP8611
L_gustavoromeroi_DB11293__L_gustavoromeroi_DB11293
L_glicensteinii_AK5818__L_glicensteinii_AK5818
L_gargantua_DB11868__L_gargantua_DB11868
L_ferrelliae_FP8806__L_ferrelliae_FP8806
L_eximia_DB9600__L_eximia_DB9600
L_elegans_DB7606__L_elegans_DB7606
L_elata_DB10554__L_elata_DB10554
L_droseroides_MF945__L_droseroides_MF945
L_dolabriformis_DB10375__L_dolabriformis_DB10375
L_disticha_AK4589__L_disticha_AK4589
L_dikoensis_DB1625__L_dikoensis_DB1625
L_demissa_DB2981__L_demissa_DB2981
L_cuspidata_AK6239__L_cuspidata_AK6239
L_cribii_FP8711__L_cribii_FP8711
L_confusa_DB3087__L_confusa_DB3087
L_cloesii_DB11877__L_cloesii_DB11877
L_cascajalensis_DB4836__L_cascajalensis_DB4836
L_caprimulgus_DB11874__L_caprimulgus_DB11874
L_candida_DB11656__L_candida_DB11656
L_calodyction_DB11872__L_calodyction_DB11872
L_calliope_DB11873__L_calliope_DB11873
L_brunnescens_DB2994__L_brunnescens_DB2994
L_bradei_AK5267__L_bradei_AK5267
L_blepharistes_FP8720__L_blepharistes_FP8720
L_blephariglossa_DB9604__L_blephariglossa_DB9604
L_atrata_DB11053__L_atrata_DB11053
L_ankistra_AK6147__L_ankistra_AK6147
F_caulescens_CL18778__F_caulescens_CL18778
Ep_ximeniae_AK6502__Ep_ximeniae_AK6502
D_aberrans_AK5978__D_aberrans_AK5978
Ac_fenestrata_JLOrchids_sn__Ac_fenestrata_JLOrchids_sn
A_rabei_DB12050__A_rabei_DB12050
A_rabei_AK4794__A_rabei_AK4794
A_peroupavae_AK5759__A_peroupavae_AK5759
A_pabstii_AK4821__A_pabstii_AK4821
A_linearifolia_JH2336__A_linearifolia_JH2336
A_lewisiae_DB1056__A_lewisiae_DB1056
Ac_cogniauxiana_AK5879__Ac_cogniauxiana_AK5879
A_burzlaffiana_AK4857__A_burzlaffiana_AK4857
0.000
0.005
0.010
0.015
0.020
Normalized PACo sqr. residuals

Appendix S8. Log-likelihoods from the ER, SYM and ARD models inferred from ultrametric trees using the re-rooting method. * probability was significantly different <0.01 favoring the more complex model.

Trait	ER	SYM	ARD	ER vs. SYM	ER vs. ARD	SYM vs. ARD	p-val ER vs. SYM	p-val ER vs. ARD	p-val SYM vs. ARD
Habit	-25.56	-25.56	-24.31	0.00	2.50	2.50	1.00	0.11	0.11
Ramicauls	-16.47	-16.47	-16.01	0.00	0.93	0.93	1.00	0.33	0.33
Bracts of ramicauls	-21.89	-20.80	-16.41	2.18	10.97	8.78	0.54	0.05	0.01
Inflorescence	-22.09	-22.09	-18.67	0.00	6.84	6.84	1.00	0.01	0.01
Inflorescence length	-27.88	-27.88	-24.47	0.00	6.83	6.83	1.00	0.01	0.01
Flowers	-5.47	-5.47	-5.43	0.00	0.08	0.08	1.00	0.78	0.78
Dorsal sepal concavity	-24.55	-24.55	-24.45	0.00	0.19	0.19	1.00	0.66	0.66
Synsepal	-20.93	-20.93	-19.90	0.00	2.08	2.08	1.00	0.15	0.15
Sepal shape	-37.48	-36.75	-35.60	1.46	3.77	2.31	0.69	0.58	0.31
Petals shape	-5.34	-5.34	-5.22	0.00	0.23	0.23	1.00	0.63	0.63
Lip shape	-16.42	-16.42	-16.11	0.00	0.63	0.63	1.00	0.43	0.43
Lip mobility	-13.41	-13.41	-12.67	0.00	1.47	1.47	1.00	0.23	0.23
Glenion of the lip	-7.73	-7.73	-7.70	0.00	0.07	0.07	1.00	0.79	0.79
Appendix of the lip	-7.57	-7.57	-7.32	0.00	0.49	0.49	1.00	0.48	0.48
Column foot	-13.58	-13.58	-12.90	0.00	1.36	1.36	1.00	0.24	0.24
Sigma shape	-7.73	-7.73	-7.70	0.00	0.07	0.07	1.00	0.79	0.79
Anther position	-13.58	-13.58	-12.90	0.00	1.36	1.36	1.00	0.24	0.24
Pollinarium shape	-13.41	-13.41	-12.67	0.00	1.47	1.47	1.00	0.23	0.23

Appendix S9. Log-likelihoods from the ER, SYM and ARD models inferred from ultrametric trees using the ACE function of APE. * probability was significantly different <0.01 favoring the more complex model.

Trait	ER	SYM	ARD	ER vs. SYM	ER vs. ARD	SYM vs. ARD	p-val ER vs. SYM	p-val ER vs. ARD	p-val SYM vs. ARD
Habit	-24.87	-24.87	-23.62	0.00	2.50	2.50	1.00	0.11	0.11
Ramicauls	-15.78	-15.78	-19.10	0.00	6.63	6.63	1.00	0.01	0.01
Bracts of ramicauls	-20.79	-19.70	-15.31	2.18	10.97	8.78	0.54	0.05	0.01
Inflorescence	-21.40	-21.40	-17.98	0.00	6.84	6.84	1.00	0.01	0.01
Inflorescence length	-27.19	-27.19	-23.77	0.00	6.83	6.83	1.00	0.01	0.01
Flowers	-4.78	-4.78	-4.74	0.00	0.08	0.08	1.00	0.78	0.78
Dorsal sepal concavity	-23.85	-23.85	-27.44	0.00	7.18	7.18	1.00	0.01	0.01
Synsepal	-20.24	-20.24	-17.39	0.00	5.70	5.70	1.00	0.02	0.02
Sepal shape	-36.38	-35.65	-32.47	1.46	7.82	6.36	0.69	0.17	0.04
Petals shape	-4.65	-4.65	-4.53	0.00	0.23	0.23	1.00	0.63	0.63
Lip shape	-15.73	-15.73	-15.41	0.00	0.63	0.63	1.00	0.43	0.43
Lip mobility	-12.71	-12.71	-11.98	0.00	1.47	1.47	1.00	0.23	0.23
Glenion of the lip	-7.04	-7.04	-7.00	0.00	0.07	0.07	1.00	0.79	0.79
Appendix of the lip	-6.88	-6.88	-6.63	0.00	0.49	0.49	1.00	0.48	0.48
Column foot	-12.89	-12.89	-12.20	0.00	1.36	1.36	1.00	0.24	0.24
Sigma shape	-7.04	-7.04	-7.00	0.00	0.07	0.07	1.00	0.79	0.79
Anther position	-12.89	-12.89	-12.20	0.00	1.36	1.36	1.00	0.24	0.24
Pollinarium shape	-12.71	-12.71	-11.98	0.00	1.47	1.47	1.00	0.23	0.23

Appendix S10. Log-likelihood ratio test estimated from the ARD, ER and SYM models inferred from phylograms using re-rooting method. * probability was significantly different <0.01 favoring the more complex model.

**Trait**	**ER**	**SYM**	**ARD**	**ER vs. SYM**	**ERvsARD**	**SYMvsARD**	**p-val SYM vs. ARD**	**p-val ER vs. SYM**	**p-val ER vs. ARD**
Habit	-28.48	-28.48	-23.50	0.00	9.96	9.96	0.02	1.00	0.08
Ramicauls	-18.95	-18.95	-18.60	0.00	0.70	0.70	0.87	1.00	0.98
Bracts of ramicauls	-22.12	-21.11	-19.60	2.01	5.03	3.01	0.39	0.37	0.41
Inflorescence	-23.36	-23.36	-18.82	0.00	9.07	9.07	0.03	1.00	0.11
Inflorescence length	-29.33	-29.33	-27.13	0.00	4.39	4.39	0.22	1.00	0.49
Flowers	-07.22	-07.22	-07.21	0.00	0.03	0.03	1.00	1.00	1.00
Dorsal sepal concavity	-22.99	-22.99	-22.67	0.00	0.64	0.64	0.89	1.00	0.99
Synsepal	-22.78	-22.78	-19.93	0.00	5.70	5.70	0.13	1.00	0.34
Sepal shape	-42.36	-41.62	-40.86	1.48	3.01	1.53	0.68	0.48	0.70
Petals shape	-06.26	-06.26	-05.51	0.00	1.50	1.50	0.68	1.00	0.91
Lip shape	-07.63	-07.63	-07.30	0.00	0.64	0.64	0.89	1.00	0.99
Lip mobility	-13.72	-13.72	-12.83	0.00	1.77	1.77	0.62	1.00	0.88
Glenion of the lip	-08.13	-8.13	-08.07	0.00	0.12	0.12	0.99	1.00	1.00
Appendix of the lip	-07.11	-07.11	-06.80	0.00	0.61	0.61	0.89	1.00	0.99
Column foot	-13.63	-13.63	-12.79	0.00	1.67	1.67	0.64	1.00	0.89
Sigma shape	-08.13	-08.13	-08.07	0.00	0.12	0.12	0.99	1.00	1.00
Anther position	-13.63	-13.63	-12.79	0.00	1.67	1.67	0.64	1.00	0.89
Pollinarium shape	-13.72	-13.72	-12.83	0.00	1.77	1.77	0.62	1.00	0.88

Appendix S11. Bayes factors (BF) of support tree model testing for dating analysis. MLE= Marginal likelihood estimation, SS=stepping stone sampler, CV=coefficient of variation, Y=Yule, BD=Birth Death-Process, BDIS=Birth-Death-Incomplete Sampling.

**Speciation model**	**MLE (SS)**	**CV**	**Y**	**BD**	**BDIS**
Y	-13860.10	0.53	0	-	-
BD	-13850.61	0.52	**18.97**	0	-
BDIS	-13852.43	0.52	15.33	-3.65	0

Appendix S12. Summary results of the top five best fitting models from the Reversible-Jump MCMC approach with BayesTraitsV3 showing rate classes, frequency of model strings, proportion of zero bin (Z%), posterior distributions of rate classes and standard deviations.

Habit: (0) cespitose ; (1) repent

**Model**	q01	q10	Frequency	Proportion
**1**	0	0	24861	0.80
**2**	Z	0	4983	0.00
**3**	0	Z	74	0.20
**4**	0	1	73	0.00
**5**	1	0	9	0.00
**Frequency (Z)**	9	4983		
**Proportion zero bin (Z%)**	0.03	16.61		
**Posterior distribution (mean)**	0.015	0.012		
**Standard deviation**	0.006	0.008		

Ramicauls: (0) non-prolific ; (1) prolific

**Model**	q01	q10	Frequency	Proportion
**1**	0	0	21718	0.72
**2**	0	Z	8201	0.27
**3**	0	1	44	0.00
**4**	1	0	36	0.00
**5**	Z	0	1	0.00
**Frequency (Z)**	1	8201		

**Proportion zero bin (Z%)**	0	27.34
**Posterior distribution (mean)**	0.009	0.007
**Standard deviation**	0.005	0.006

Bracts of ramicauls: (0) unornamented ; (1) ornamented; (2) foliaceous

**Model**	**q01**	**q02**	**q10**	**q12**	**q20**	**q21**	**Frequency**	**Proportion**
**1**	0	Z	Z	0	Z	0	3801	0.13
**2**	0	Z	Z	0	0	0	3613	0.12
**3**	0	Z	0	0	Z	0	1936	0.06
**4**	0	Z	0	0	0	0	1870	0.06
**5**	0	Z	0	0	Z	Z	1870	0.06
**Frequency (Z)**	5898	17122	15117	5034	13805	8161		
**Proportion zero bin (Z%)**	19.66	57.07	50.39	16.78	46.02	27.2		
**Posterior distribution (mean)**	0.011	0.009	0.005	0.010	0.066	0.133		
**Standard deviation**	0.012	0.038	0.006	0.019	0.832	1.313		

Inflorescence: (0) simultaneous ; (1) successive

**Model**	q01	q10	Frequency	Proportion
**1**	0	Z	24225	0.81
**2**	0	0	5270	0.18
**3**	Z	0	365	0.01
**4**	1	0	72	0.00
**5**	0	1	68	0.00
**Frequency (Z)**	365	24225		
**Proportion zero bin (Z%)**	1.22	80.75		
**Posterior distribution (mean)**	0.020	0.003		
**Standard deviation**	0.010	0.007		

Inflorescence length: (0) shorter than leaves ; (1) longer than leaves

**Model**	q01	q10	Frequency	Proportion
**1**	Z	0	25007	0.83
**2**	0	0	4763	0.16
**3**	1	0	116	0.00
**4**	0	1	102	0.00
**5**	0	Z	12	0.00
**Frequency (Z)**	25007	12		
**Proportion zero bin (Z%)**	83.36	0.04		
**Posterior distribution (mean)**	0.004	0.035		
**Standard deviation**	0.010	0.013		

Flowers: (0) fully opening; (1) bud-like

**Model**	q01	q10	Frequency	Proportion
**1**	0	0	23904	0.80
**2**	0	Z	6027	0.20
**3**	Z	0	34	0.00
**4**	0	1	23	0.00
**5**	1	0	12	0.00
**Frequency (Z)**	34	6027		
**Proportion zero bin (Z%)**	0.11	20.09		
**Posterior distribution (mean)**	0.002	0.002		
**Standard deviation**	0.002	0.002		

Dorsal sepal concavity: (0) concave; (1) flattened

Model	q01	q10	Frequency	Proportion
**1**	0	0	23944	0.80
**2**	0	Z	6025	0.20
**3**	1	0	18	0.00
**4**	0	1	13	0.00
**Frequency (Z)**	11	29		
**Proportion zero bin (Z%)**	0.04	0.1		
**Posterior distribution (mean)**	0.013	0.013		
**Standard deviation**	0.006	0.006		

Synsepal: (0) absent ; (1) present

**Model**	q01	q10	Frequency	Proportion
**1**	0	0	13682	0.46
**2**	0	Z	13583	0.45
**3**	Z	0	2558	0.09
**4**	1	0	101	0.00
**5**	0	1	76	0.00
**Frequency (Z)**	2558	13583		
**Proportion zero bin (Z%)**	8.53	45.28		
**Posterior distribution (mean)**	0.018	0.007		
**Standard deviation**	0.014	0.008		

Sepals shape: (0) unornamented ; (1) ornamented; (2) foliaceous

**Model**	**q01**	**q02**	**q10**	**q12**	**q20**	**q21**	**Frequency**	**Proportion**
**1**	0	0	0	Z	Z	Z	4720	0.16
**2**	0	0	Z	0	Z	Z	2917	0.10
**3**	0	Z	Z	Z	0	0	2280	0.08

**4**	Z	0	Z	Z	0	0	2054	0.07
**5**	Z	Z	0	Z	0	0	1711	0.06
**Frequency (Z)**	9442	10113	15152	17531	9929	13132		
**Proportion zero bin (Z%)**	31.47	33.71	50.51	58.44	33.1	43.77		
**Posterior distribution (mean)**	0.027	0.030	0.022	0.014	0.013	0.010		
**Standard deviation**	0.030	0.034	0.034	0.022	0.012	0.010		

Petals shape: (0) subsimilar ; (1) disimilar

Model	q01	q10	Frequency	Proportion
**1**	0	0	23944	0.80
**2**	0	Z	6025	0.20
**3**	1	0	18	0.00
**4**	0	1	13	0.00
**Frequency (Z)**	0	6025		
**Proportion zero bin (Z%)**	0	20.08		
**Posterior distribution (mean)**	0.018	0.007		
**Standard deviation**	0.014	0.008		

Lip shape: (0) laminar ; (1) bilobed

Model	q01	q10	Frequency	Proportion
**1**	0	0	23944	0.80
**2**	0	Z	6025	0.20
**3**	1	0	18	0.00
**4**	0	1	13	0.00
**Frequency (Z)**	0	7033		
**Proportion zero bin (Z%)**	0	23.44		
**Posterior distribution (mean)**	0.002	0.002		
**Standard deviation**	0.002	0.002		

Lip mobility: (0) motile ; (1) sessile

Model	q01	q10	Frequency	Proportion
**1**	0	0	20653	20653.00
**2**	0	Z	9285	9285.00
**3**	0	1	34	34.00
**4**	1	0	28	28.00
**Frequency (Z)**	0	9185		
**Proportion zero bin (Z%)**	0	30.95		
**Posterior distribution (mean)**	0.005	0.003		
**Standard deviation**	0.003	0.003		

Glenion of the lip: (0) abscent ; (1) present

Model	q01	q10	Frequency	Proportion
**1**	0	0	23833	0.79
**2**	0	Z	6141	0.20
**3**	1	0	15	0.00
**4**	0	1	11	0.00
**Frequency (Z)**	0	6141		
**Proportion zero bin (Z%)**	0	20.47		
**Posterior distribution (mean)**	0.002	0.002		
**Standard deviation**	0.002	0.002		

Appendix of the lip: (0) absent ; (1) present

Model	q01	q10	Frequency	Proportion
**1**	0	0	20751	0.69
**2**	0	Z	9185	0.00
**3**	1	0	34	0.00
**4**	0	1	30	0.31
**Frequency (Z)**	0	7820		
**Proportion zero bin (Z%)**	0.11	20.09		
**Posterior distribution (mean)**	0.004	0.003		
**Standard deviation**	0.003	0.003		

Column foot: (0) absent ; (1) present

Model	q01	q10	Frequency	Proportion
**1**	0	0	20688	0.69
**2**	Z	0	9249	0.31
**3**	1	0	36	0.00
**4**	0	1	27	0.00
**Frequency (Z)**	9249	0		
**Proportion zero bin (Z%)**	30.83	0		
**Posterior distribution (mean)**	0.003	0.005		
**Standard deviation**	0.003	0.003		

Sigma shape: (0) entire ; (1) bilobed

Model	q01	q10	Frequency	Proportion
**1**	0	0	23772	0.79
**2**	0	Z	6199	0.21
**3**	0	1	16	0.00
**4**	1	0	13	0.00
**Frequency (Z)**	0	9185		

**Proportion zero bin (Z%)**	0	20.66		
**Posterior distribution (mean)**	0.002	0.002		
**Standard deviation**	0.002	0.002		

Anther position: (0) ventral ; (1) dorsal

**Model**	**q01**	**q10**	**Frequency (model)**	**Proportion**
**1**	0	0	20751	0.69
**2**	0	Z	9185	0.31
**3**	1	0	34	0.00
**4**	0	1	30	0.00
**Frequency (Z)**	0	9185		
**Proportion zero bin (Z%)**	0.000	30.620		
**Posterior distribution (mean)**	0.005	0.003		
**Standard deviation**	0.003	0.003		

Pollinarium: (0) with caudicles; (1) with caudicles+viscidium

**Model**	**q01**	**q10**	**Frequency**	**Proportion**
**1**	0	0	20541	20541.00
**2**	0	Z	9404	9404.00
**3**	1	0	28	28.00
**4**	0	1	27	27.00
**Frequency (Z)**	0	9185		
**Proportion zero bin (Z%)**	0	31.35		
**Posterior distribution (mean)**	0.005	0.003		
**Standard deviation**	0.003	0.003		

Appendix S13. Posterior distributions of the rate coefficients based on Reversible-Jump approach of BayesTraitsV3.

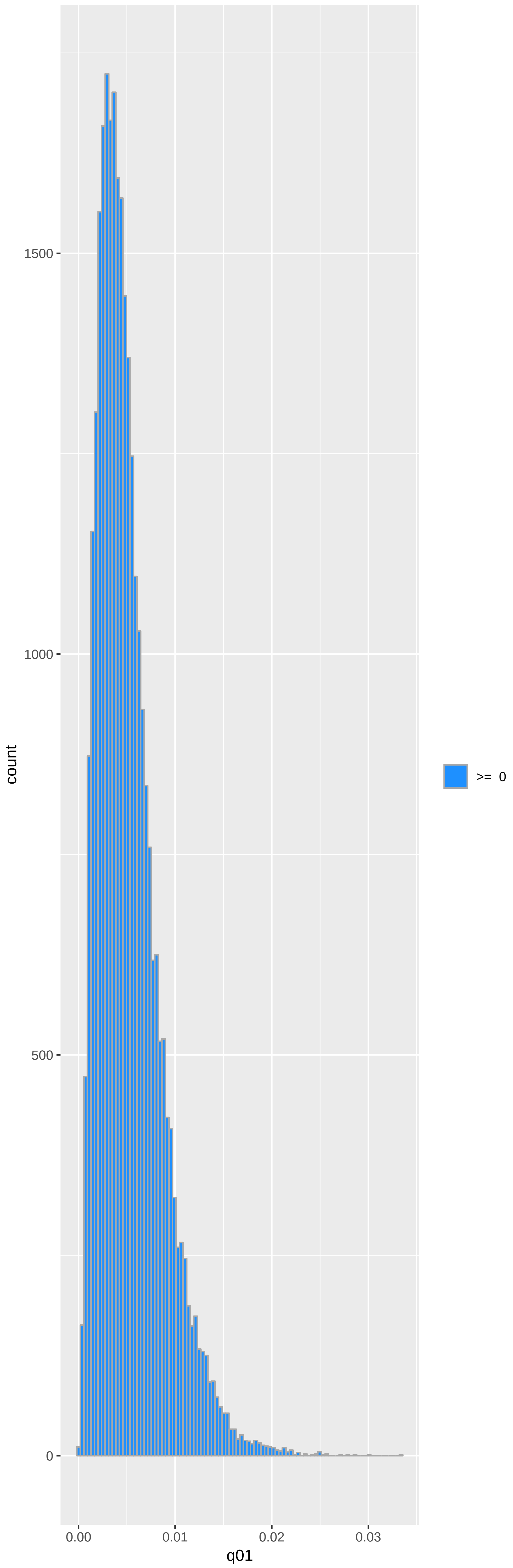

1500
1000
500
0
count
0.00
0.01
0.02
0.03
q01
>= 0

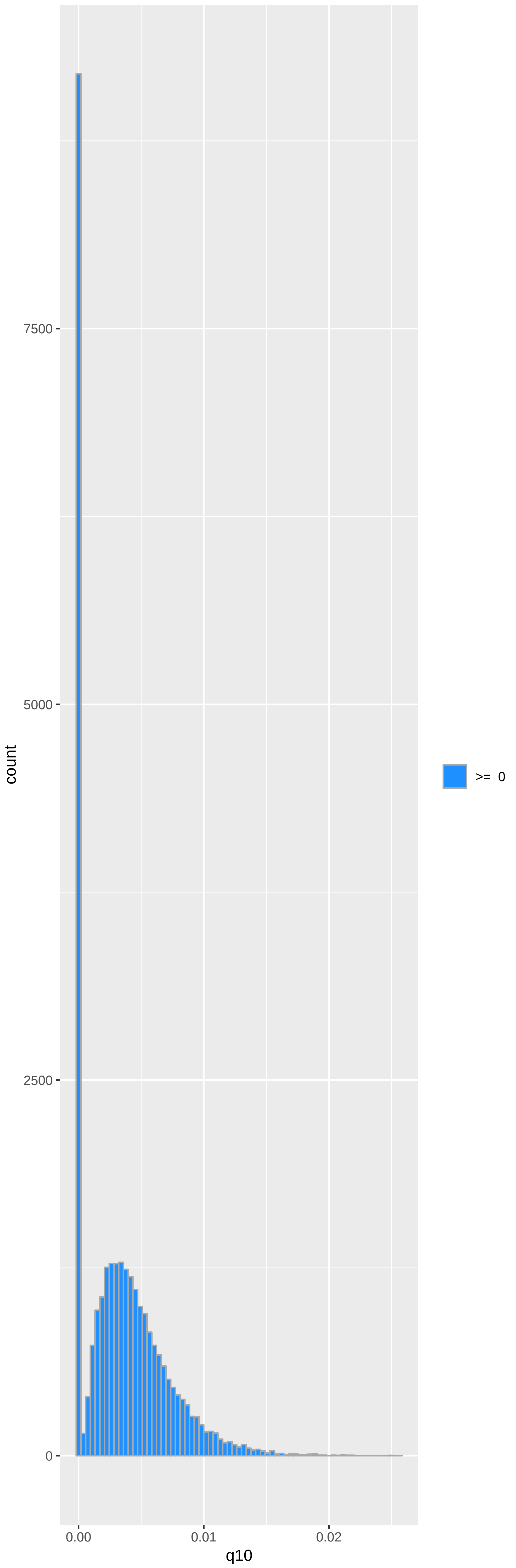

7500
5000
2500
0
count
0.00
0.01
0.02
q10
>= 0

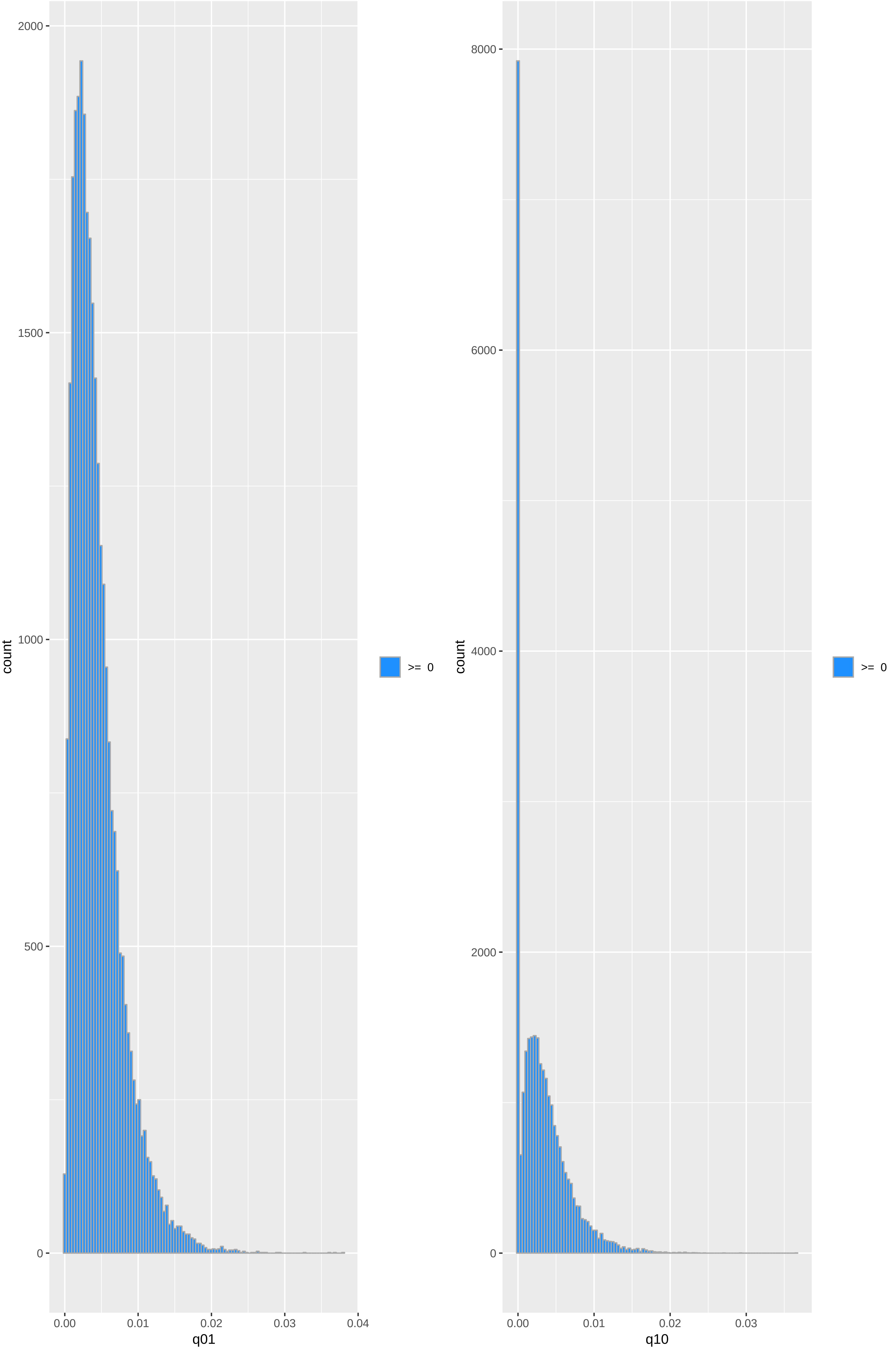
2000
1500
1000
500
0
count
0.00
0.01
0.02
0.03
0.04
q01
>= 0
8000
6000
4000
2000
0
count
0.00
0.01
0.02
0.03
q10
>= 0

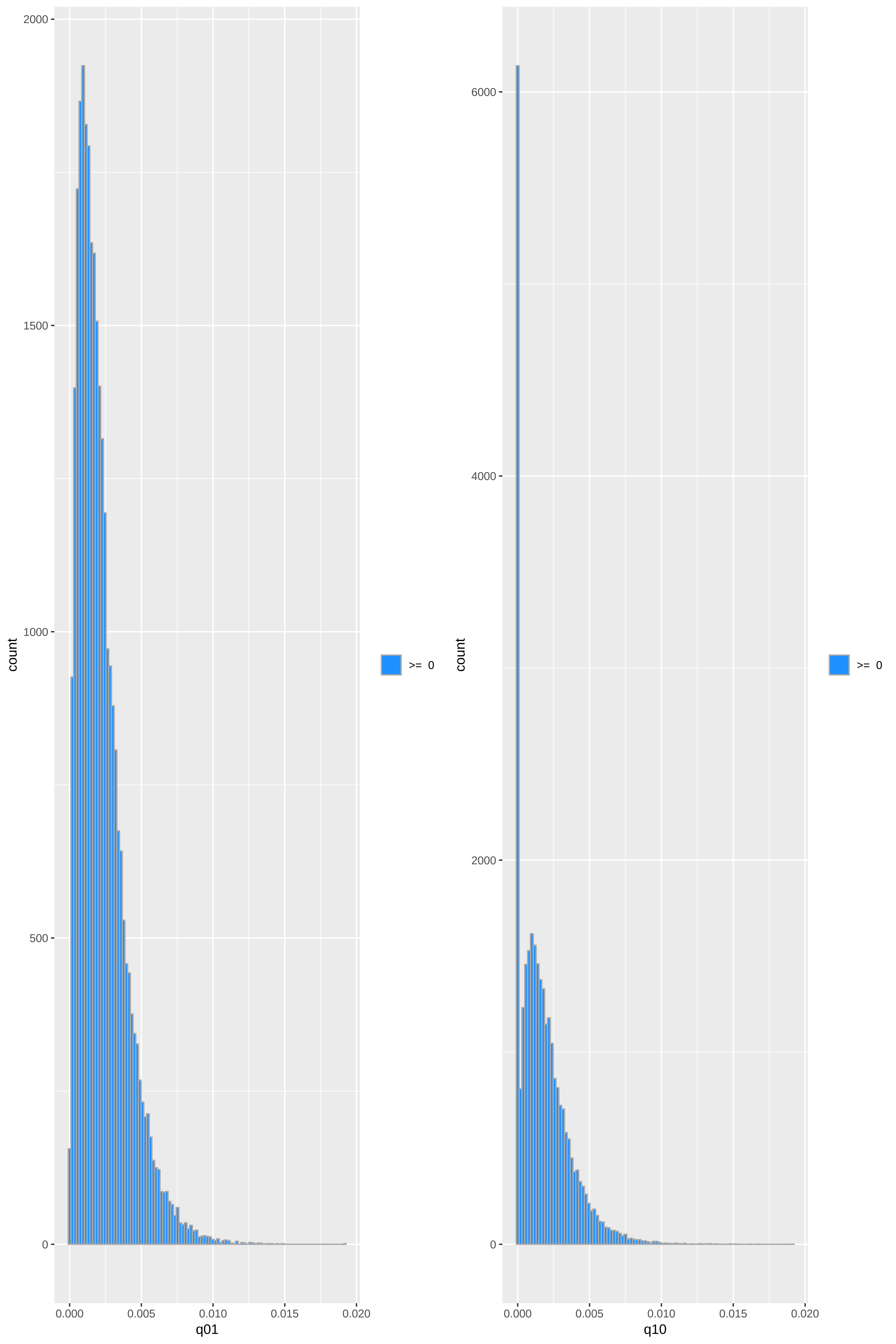
2000
1500
1000
500
0
count
>= 0
0.000
0.005
0.010
0.015
0.020
q01
6000
4000
2000
0
count
>= 0
0.000
0.005
0.010
0.015
0.020
q10

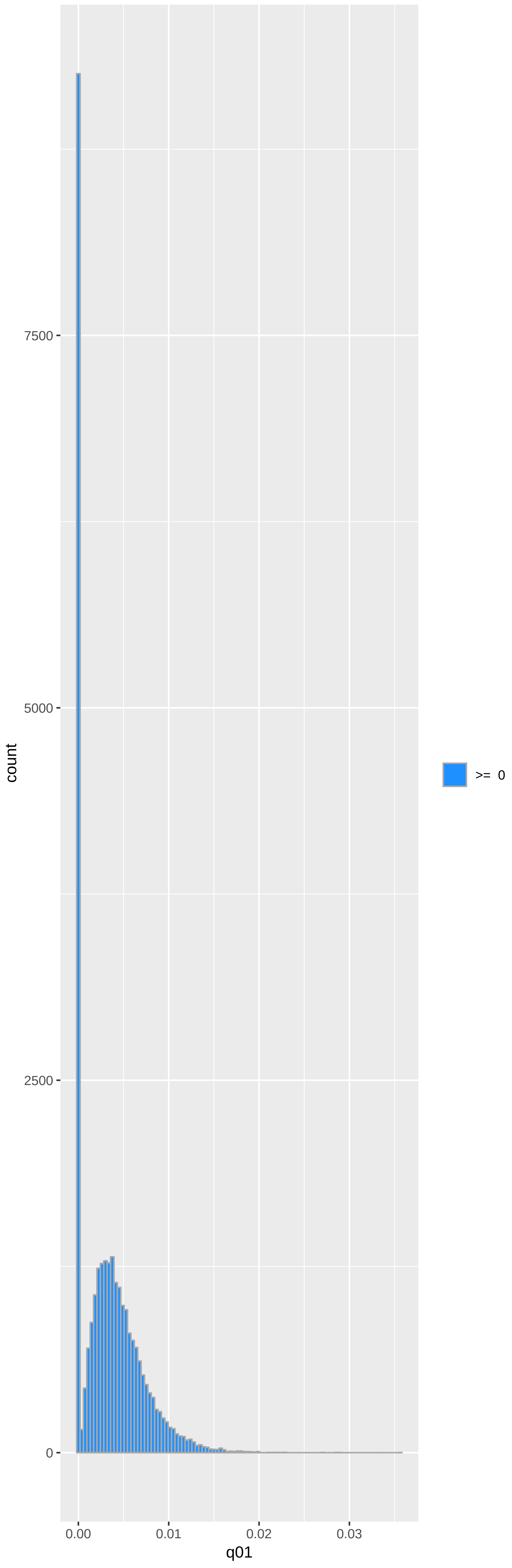

count
7500
5000
2500
0
0.00
0.01
0.02
0.03
q01
>= 0

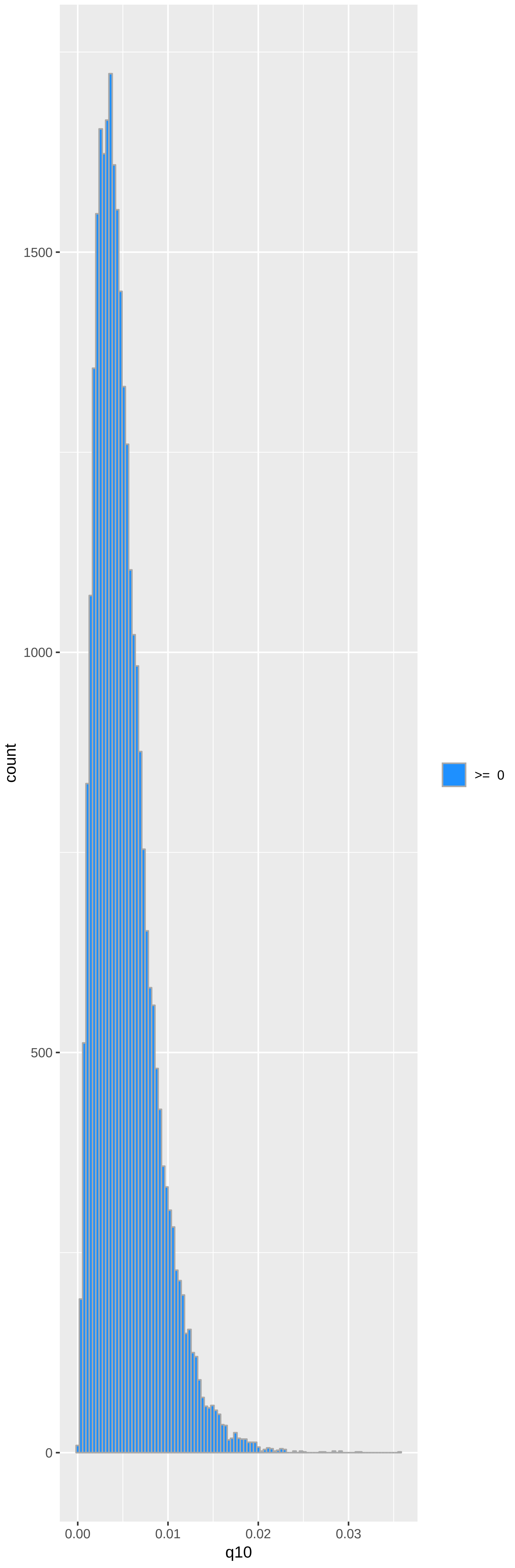

count
1500
1000
500
0
0.00
0.01
0.02
0.03
q10
>= 0

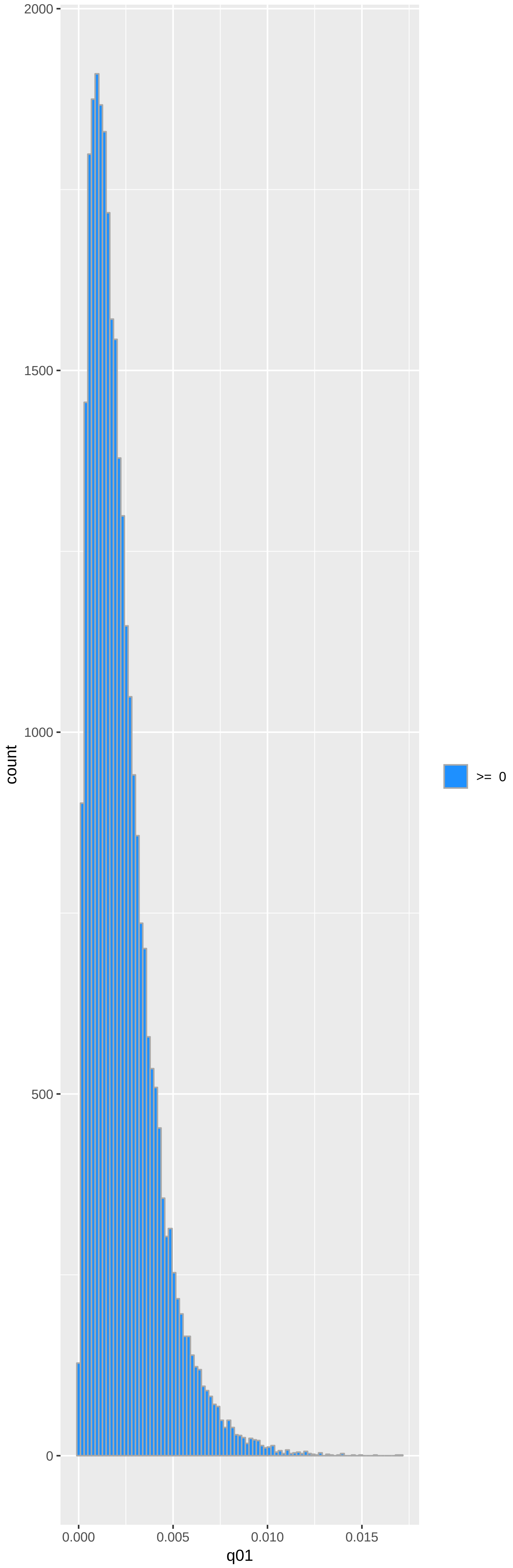

2000
1500
1000
500
0
count
0.000
0.005
0.010
0.015
q01
>= 0

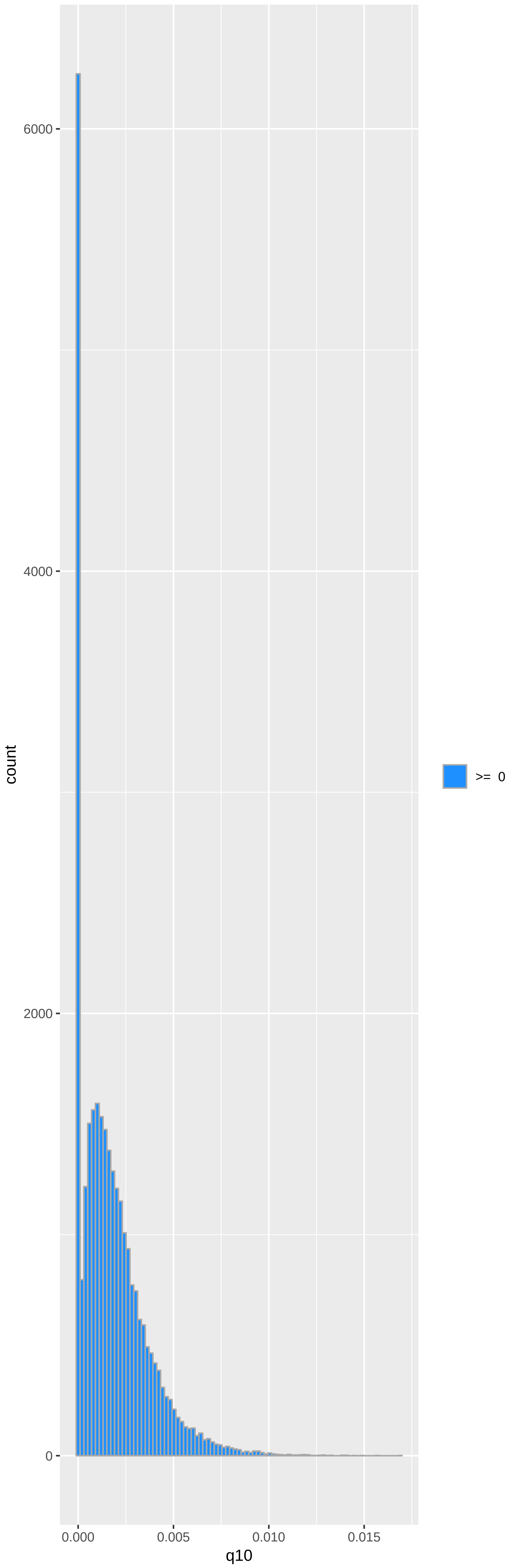

6000
4000
2000
0
count
0.000
0.005
0.010
0.015
q10
>= 0

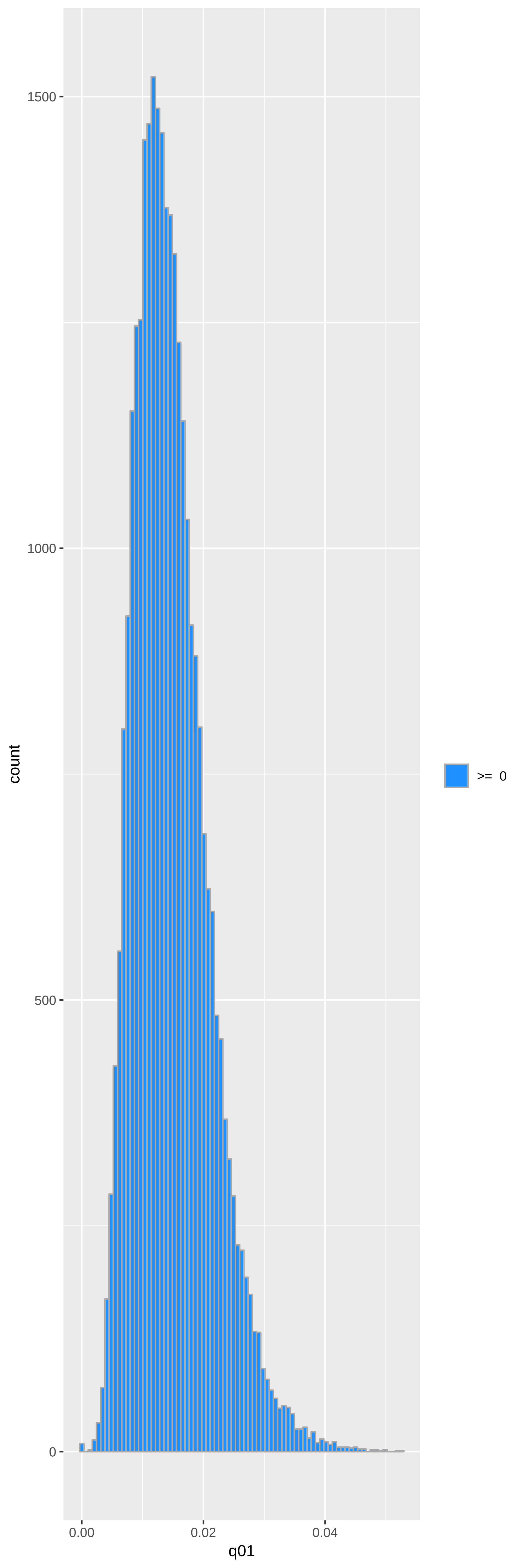

1500
1000
500
0
count
>= 0
0.00
0.02
0.04
q01

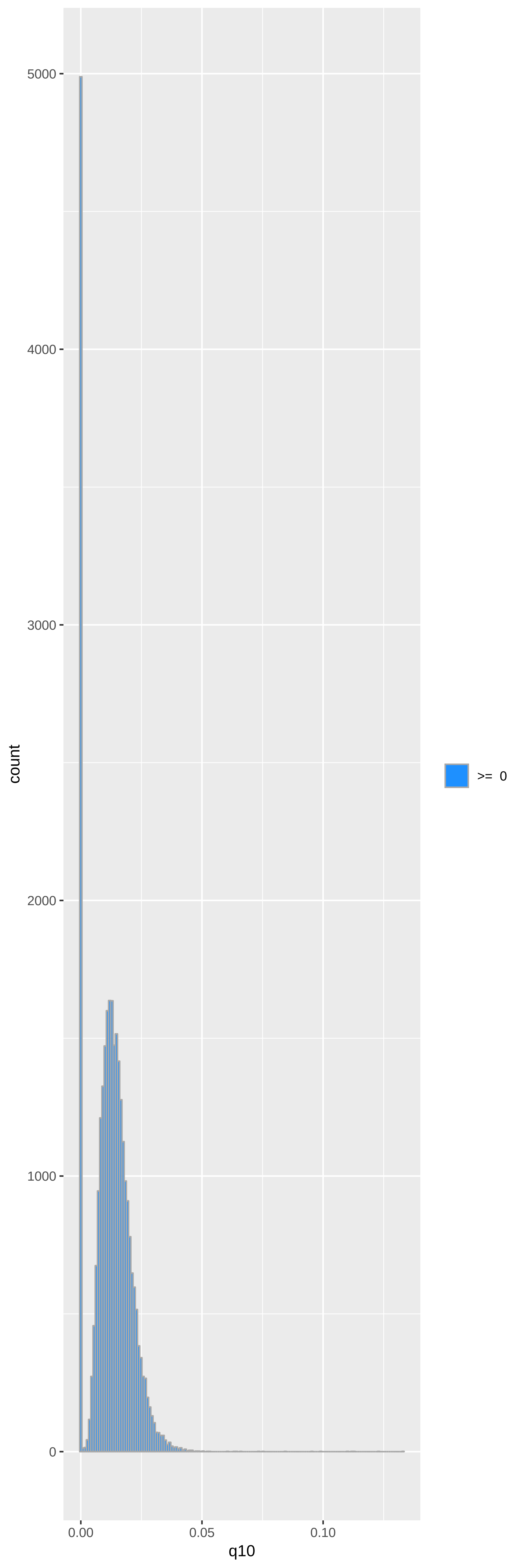

5000
4000
3000
2000
1000
0
count
>= 0
0.00
0.05
0.10
q10

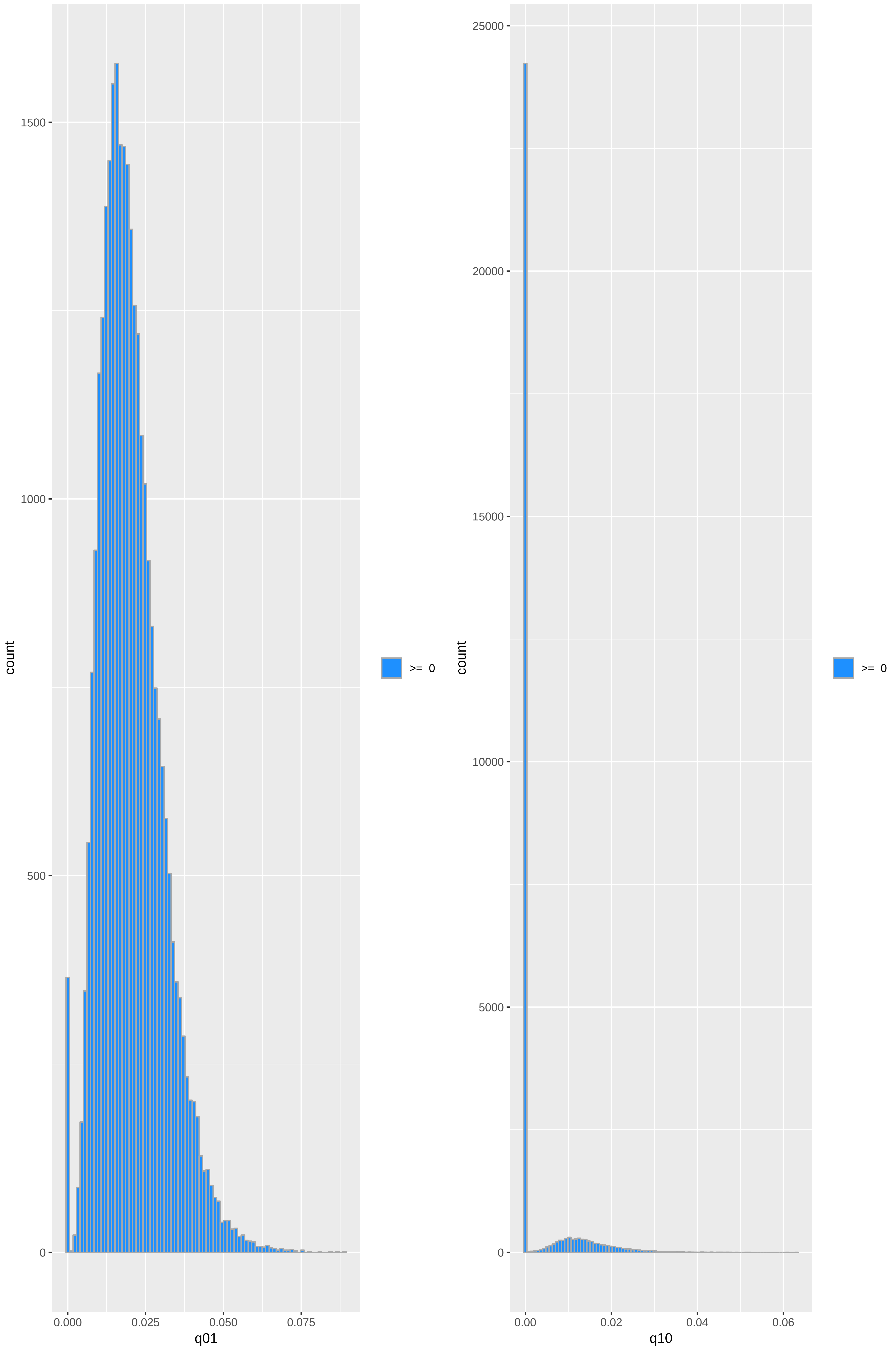
count
1500
1000
500
0
0.000
0.025
0.050
0.075
q01
>= 0
count
25000
20000
15000
10000
5000
0
0.00
0.02
0.04
0.06
q10
>= 0

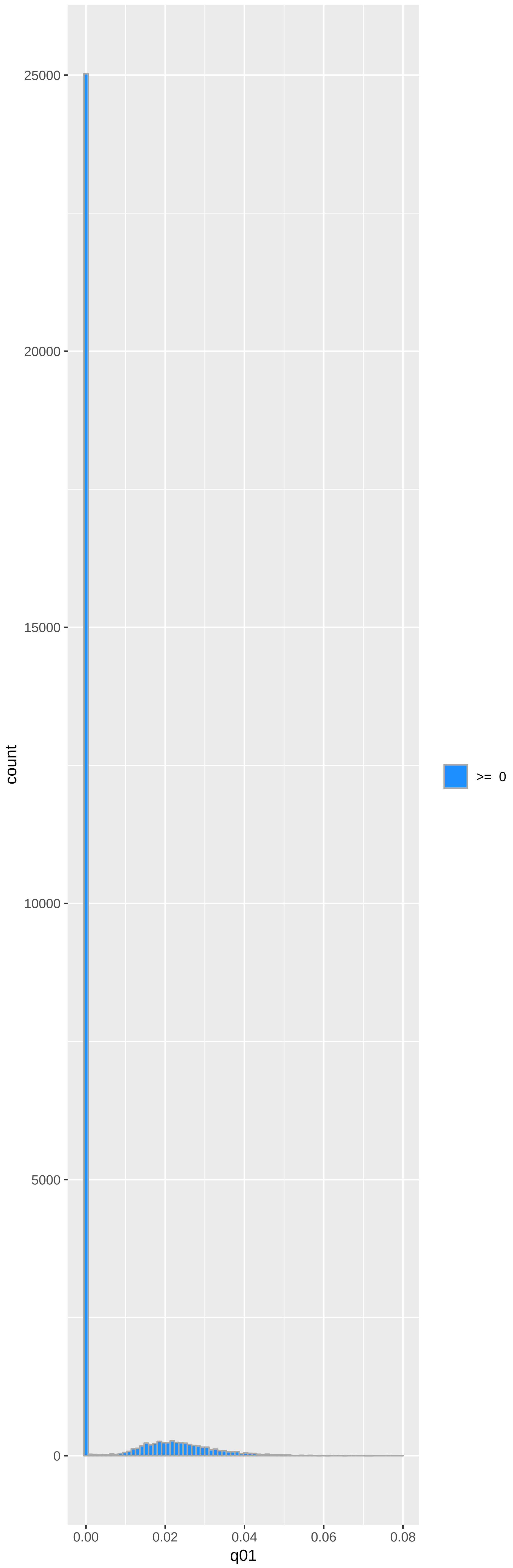
25000
20000
15000
10000
5000
0
count
>= 0
0.00
0.02
0.04
0.06
0.08
q01

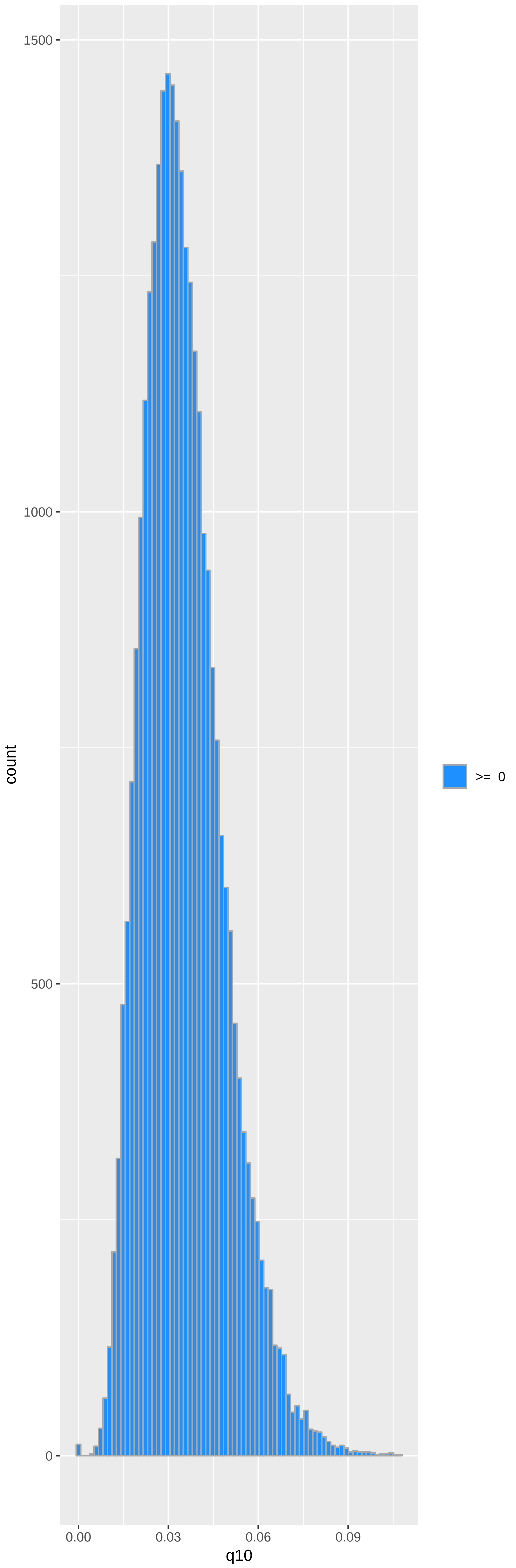
1500
1000
500
0
count
>= 0
0.00
0.03
0.06
0.09
q10

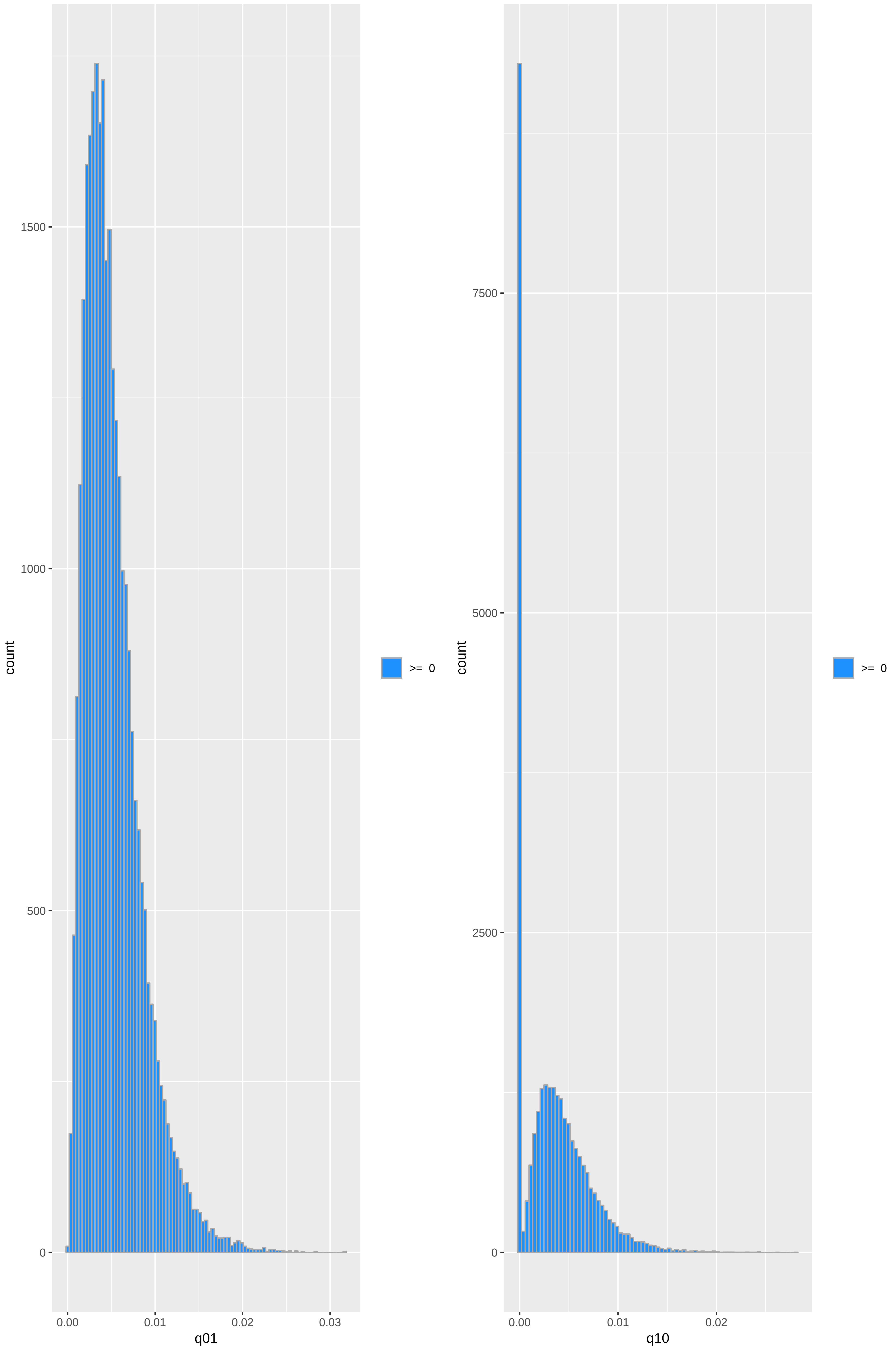
count
1500
1000
500
0
0.00
0.01
0.02
0.03
q01
>= 0
count
7500
5000
2500
0
0.00
0.01
0.02
q10
>= 0

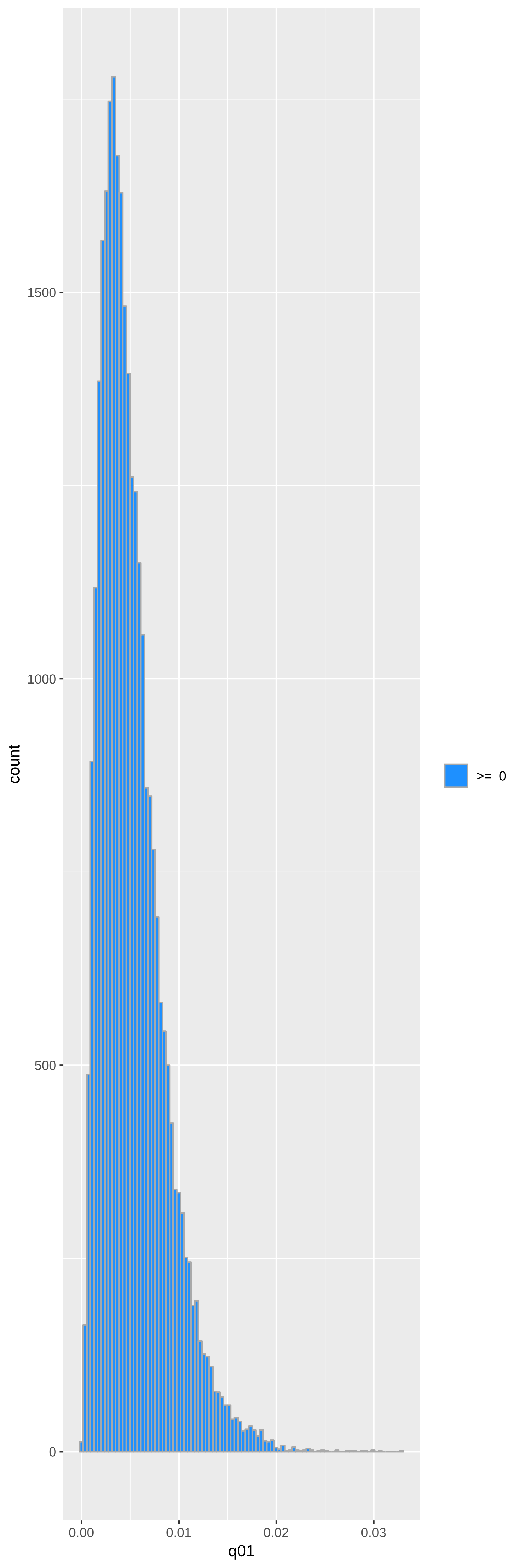

1500
1000
500
0
count
0.00
0.01
0.02
0.03
q01
>= 0

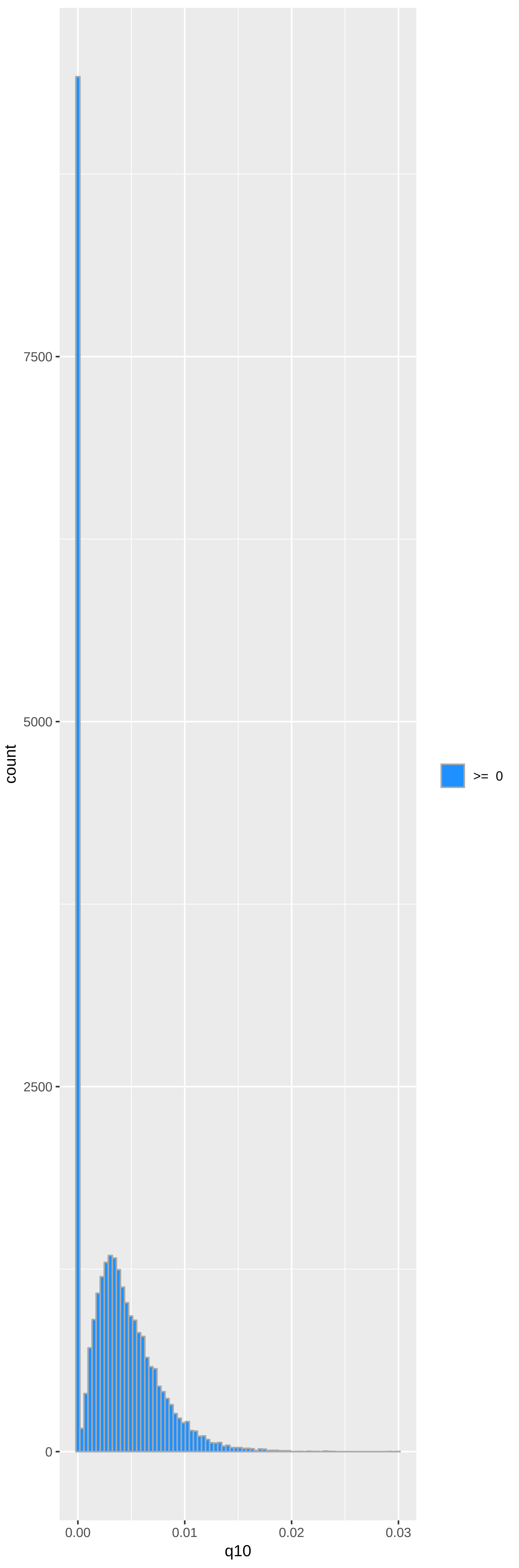

7500
5000
2500
0
count
0.00
0.01
0.02
0.03
q10
>= 0

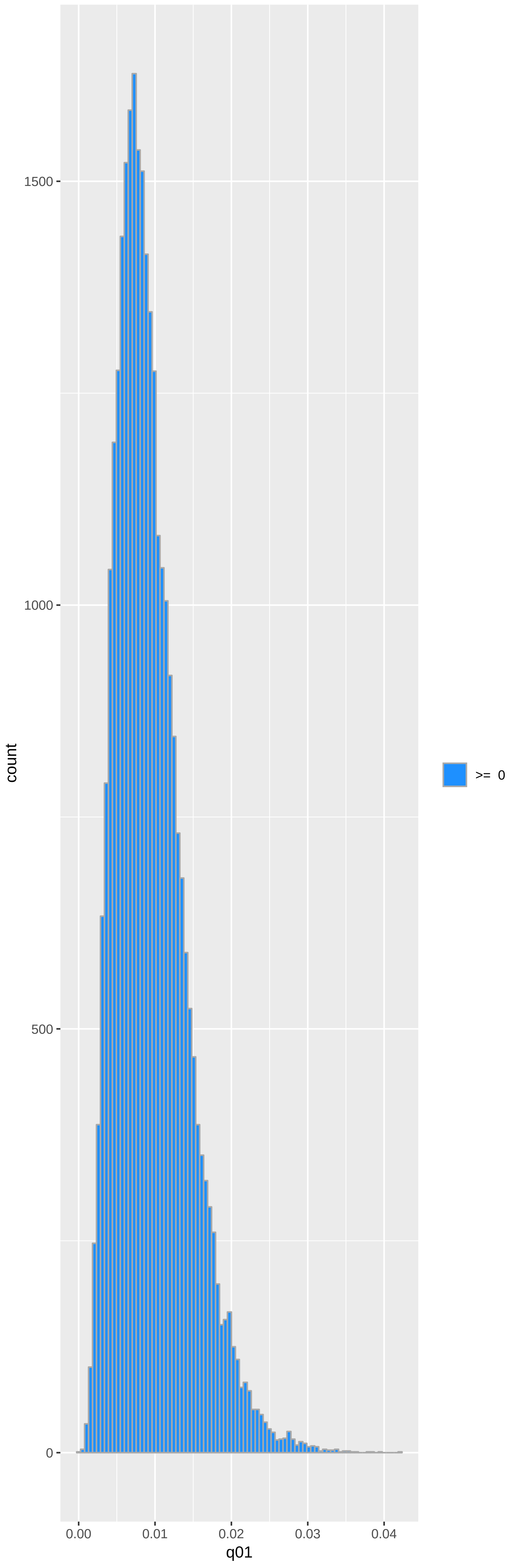

count
1500
1000
500
0
0.00
0.01
0.02
0.03
0.04
q01
>= 0

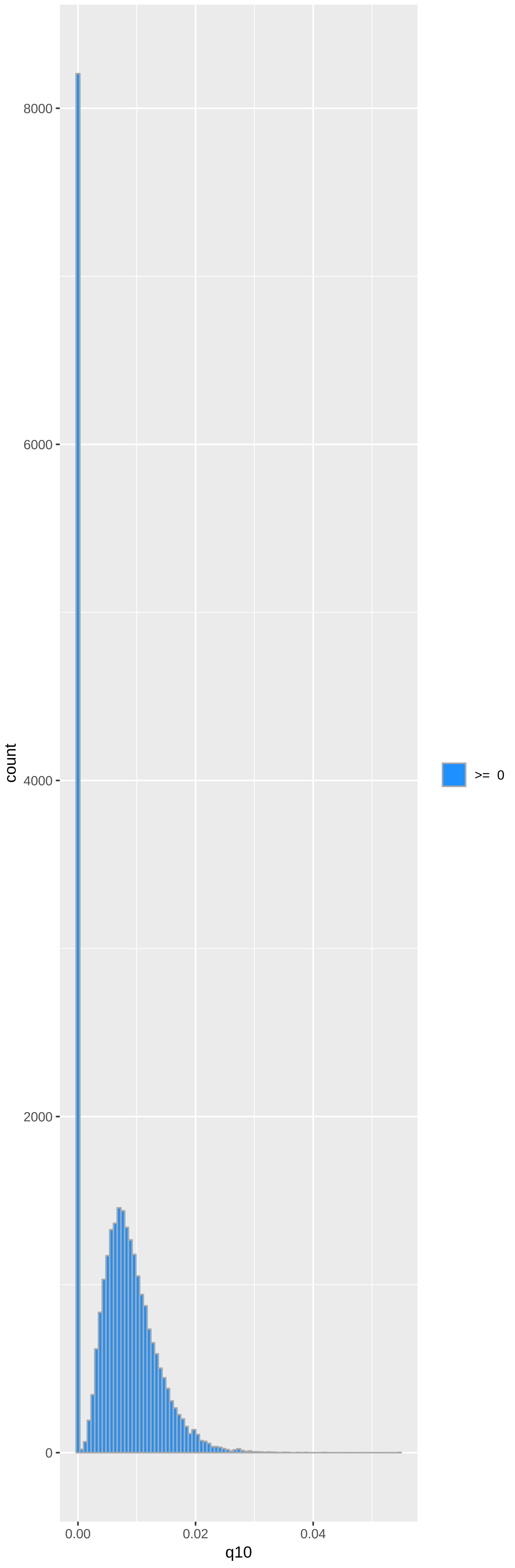

count
8000
6000
4000
2000
0
0.00
0.02
0.04
q10
>= 0

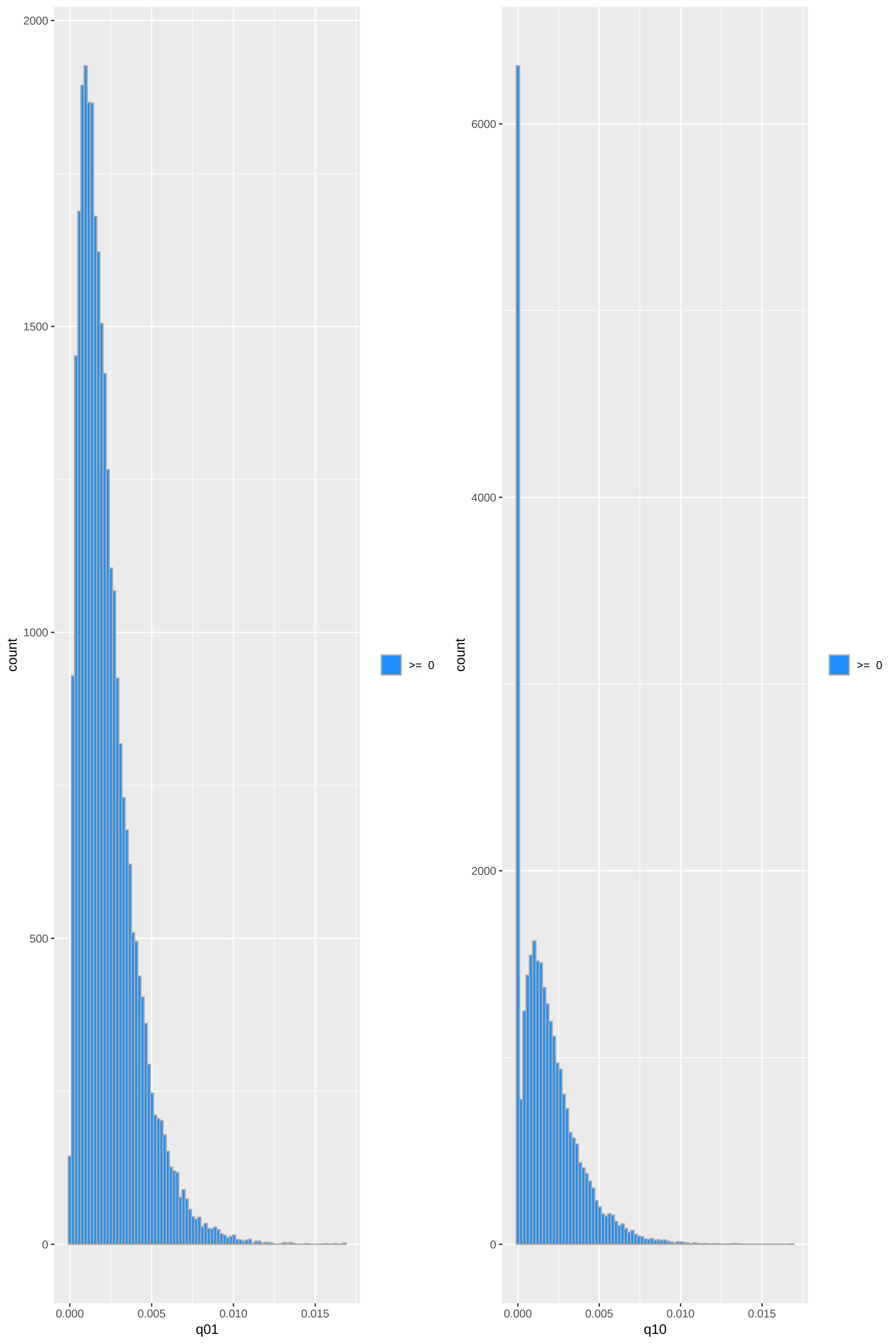

2000
1500
1000
500
0
count
0.000
0.005
0.010
0.015
q01
>= 0
6000
4000
2000
0
count
0.000
0.005
0.010
0.015
q10
>= 0

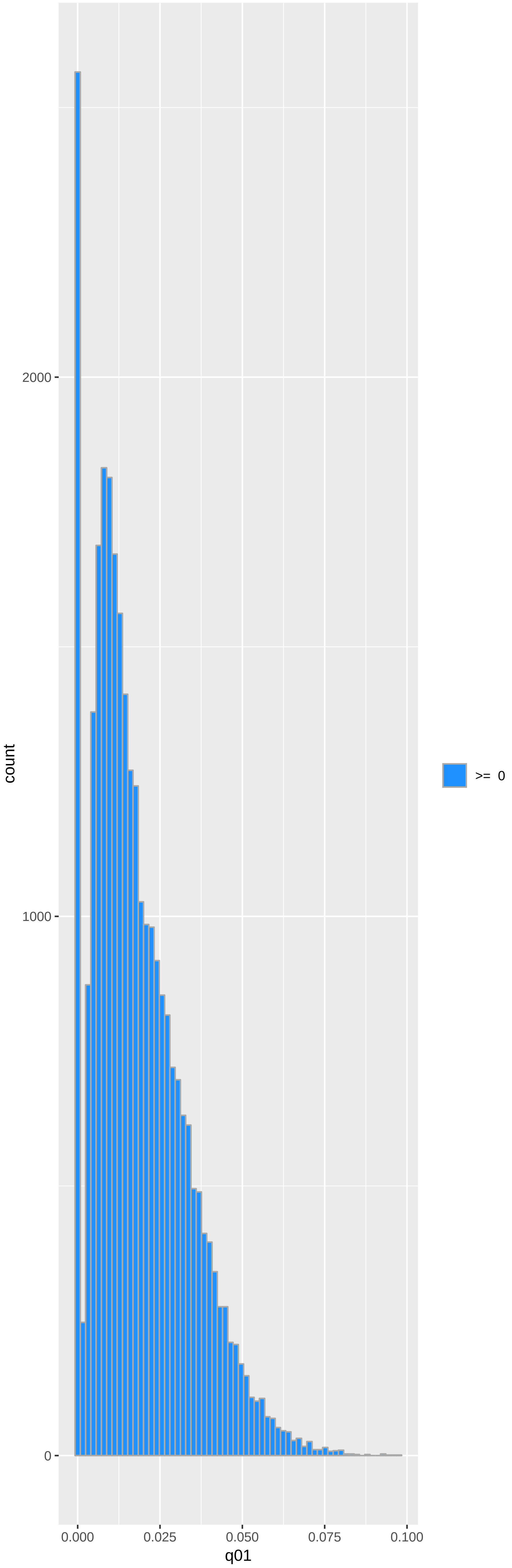

count
2000
1000
0
0.000
0.025
0.050
0.075
0.100
q01
>= 0

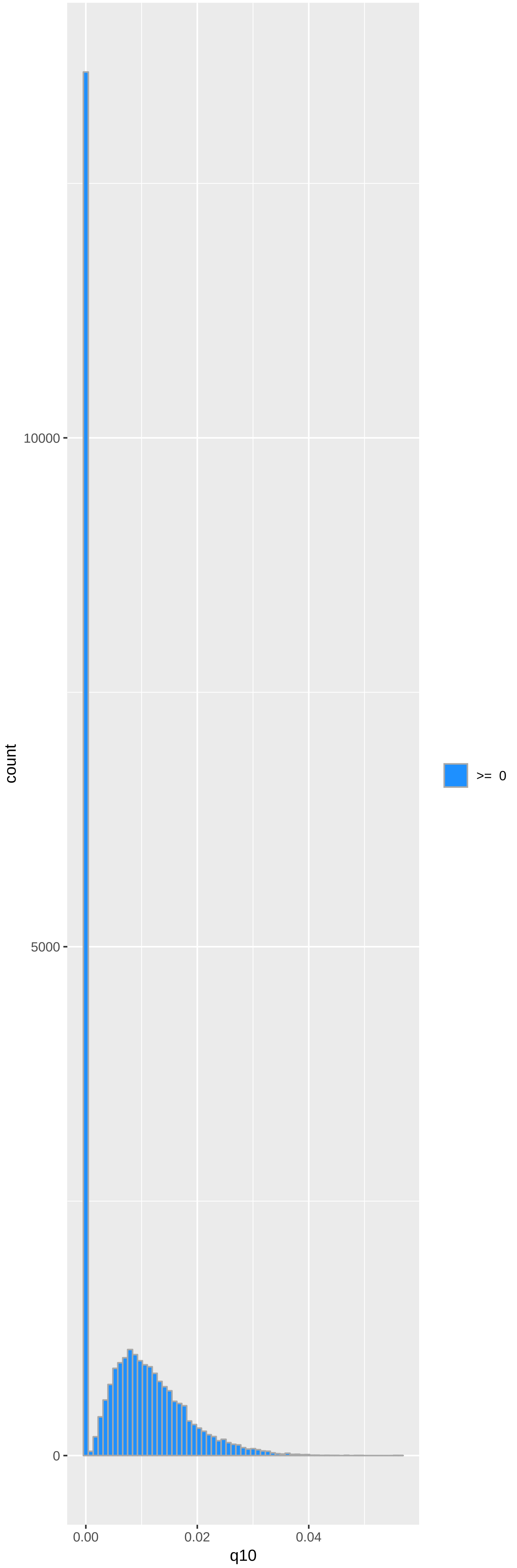

count
10000
5000
0
0.00
0.02
0.04
q10
>= 0

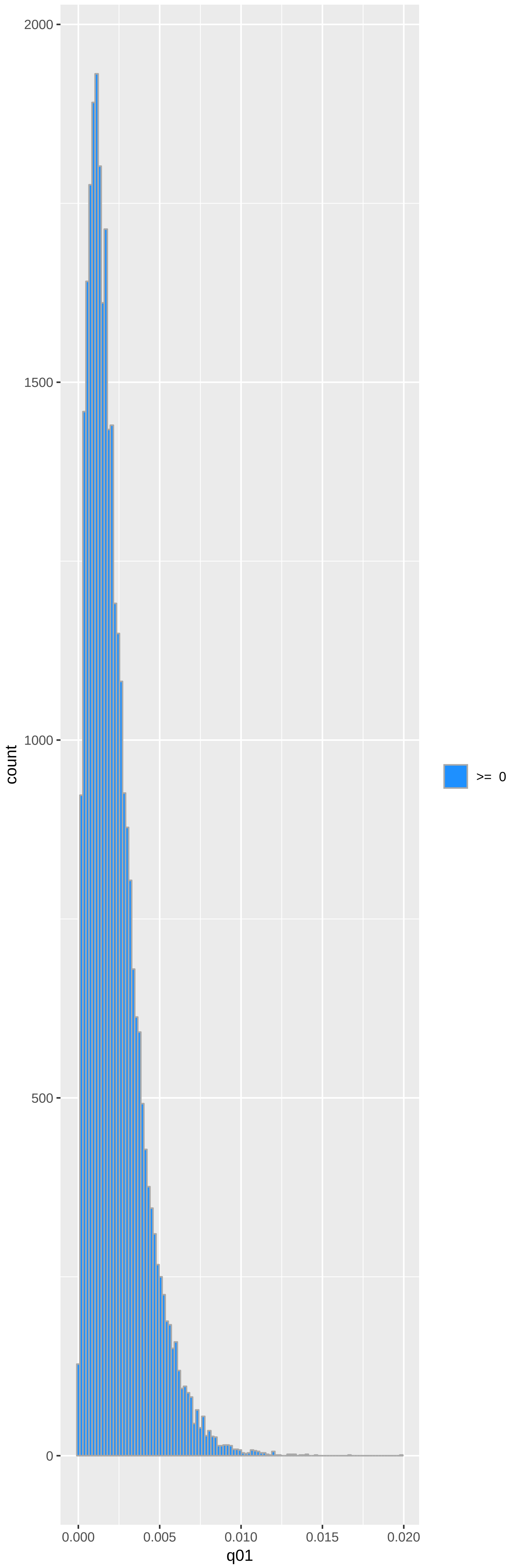
2000
1500
1000
500
0
count
0.000
0.005
0.010
0.015
0.020
q01
>= 0

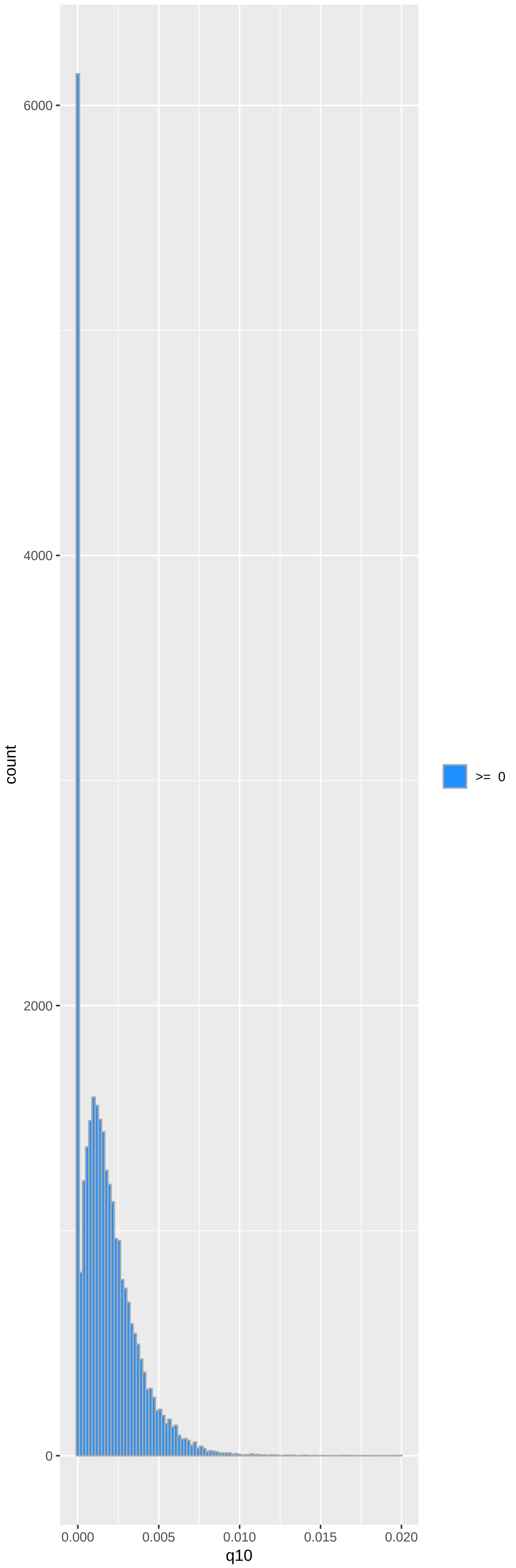
6000
4000
2000
0
count
0.000
0.005
0.010
0.015
0.020
q10
>= 0

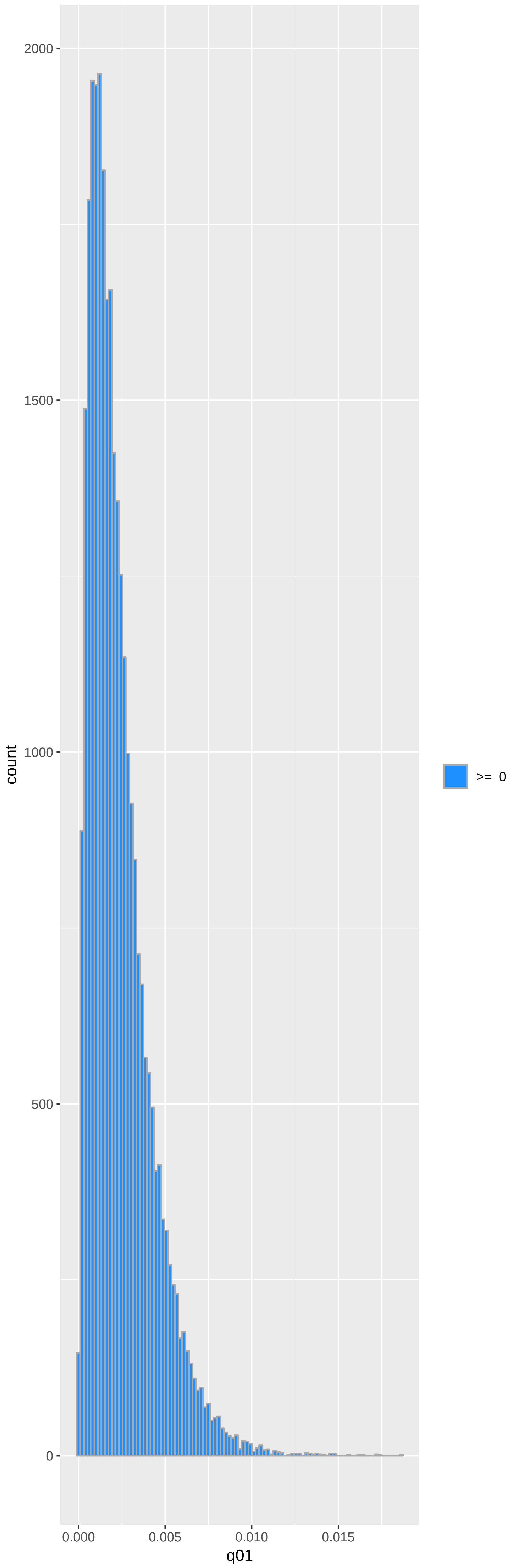
2000
1500
1000
500
0
count
0.000
0.005
0.010
0.015
q01
>= 0

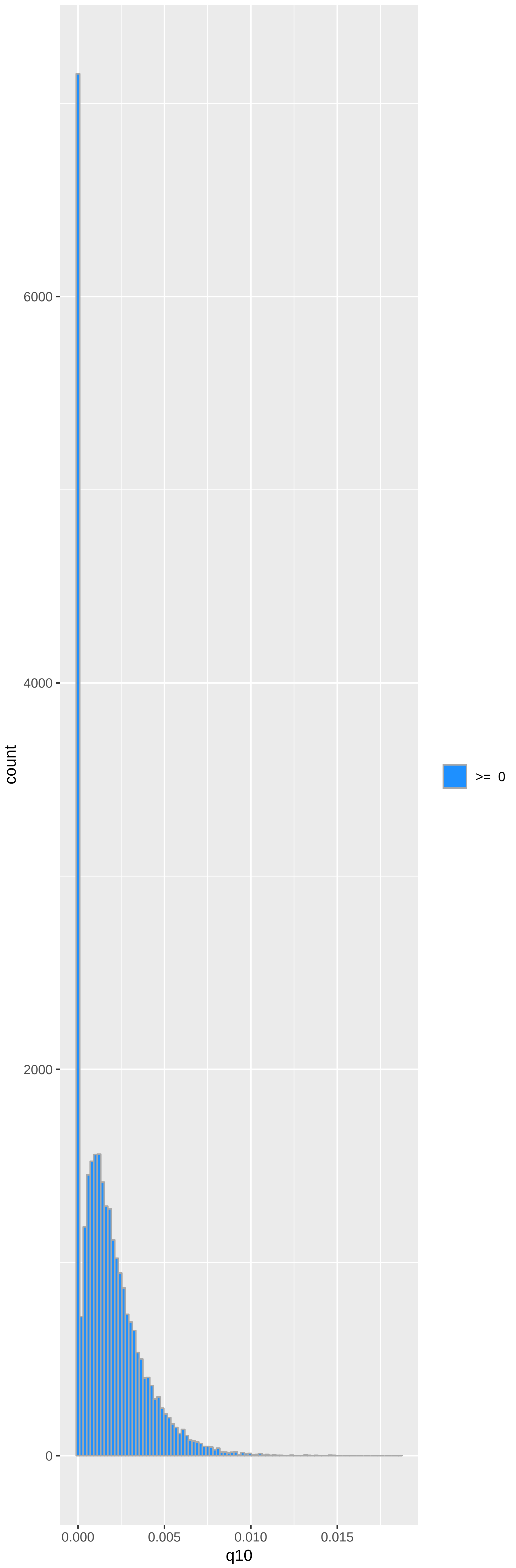
6000
4000
2000
0
count
0.000
0.005
0.010
0.015
q10
>= 0

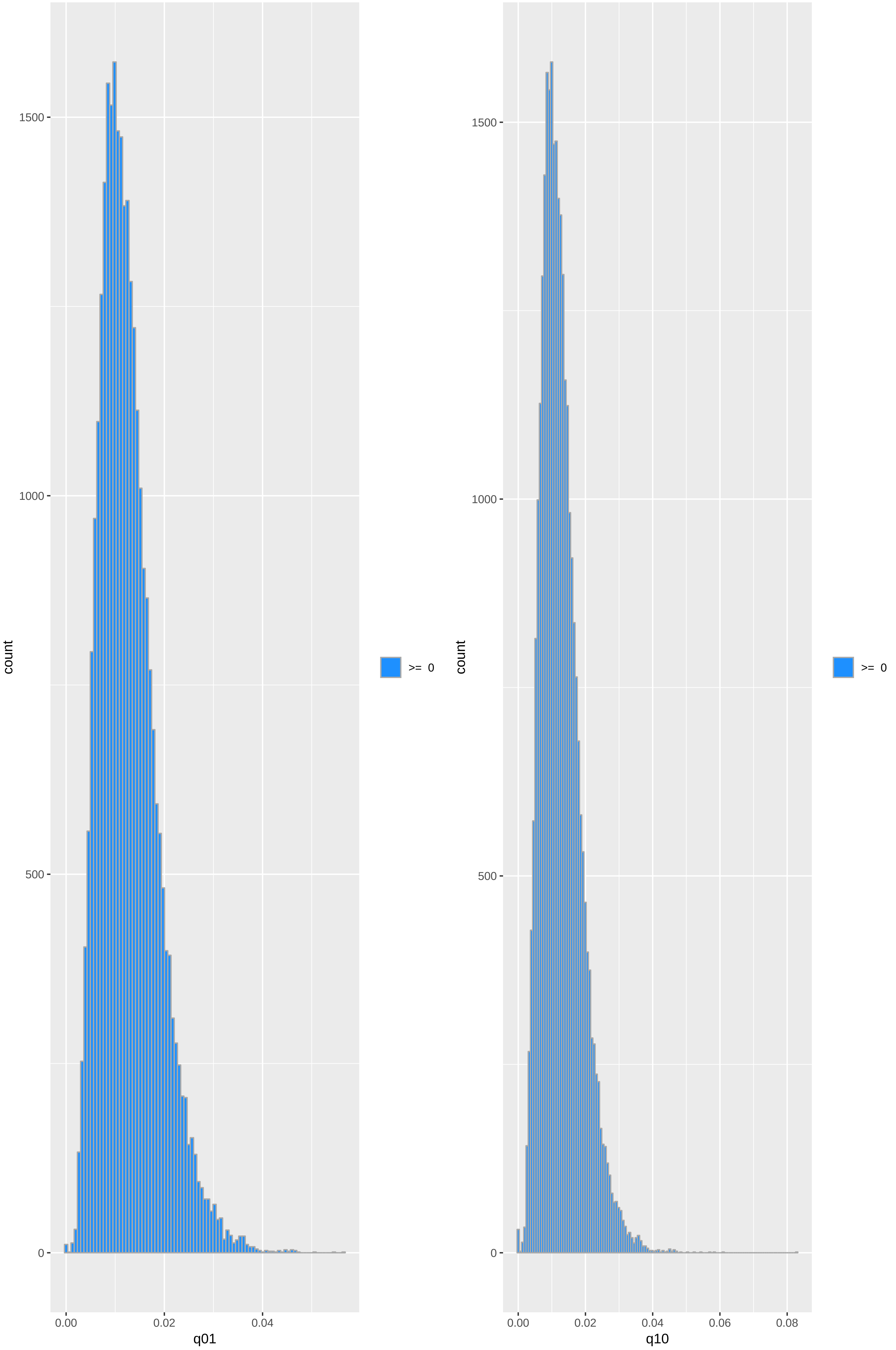

count
1500
1000
500
0
0.00
0.02
0.04
q01
>= 0
count
1500
1000
500
0
0.00
0.02
0.04
0.06
0.08
q10
>= 0

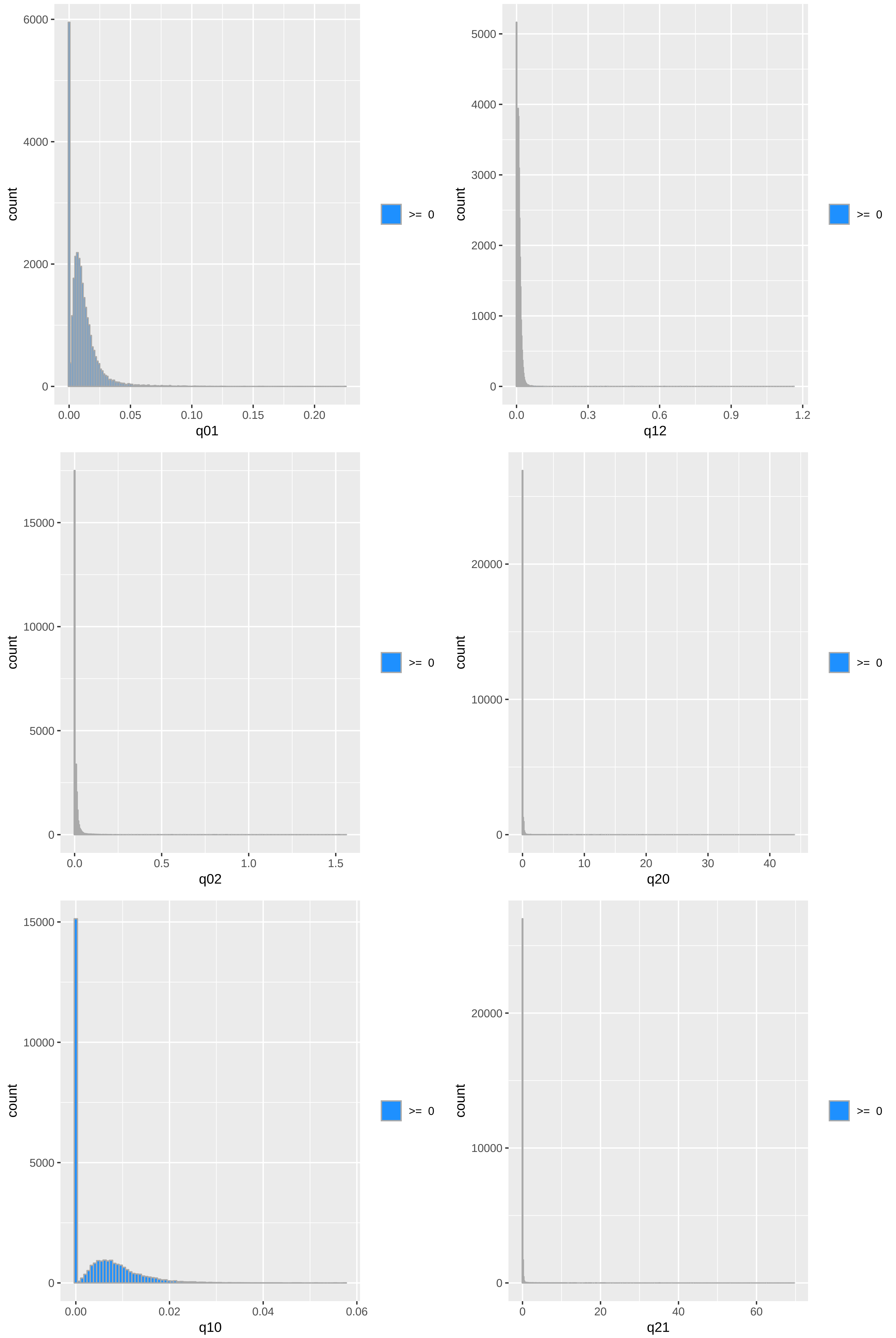
count
q01
q12
q02
q20
q10
q21
>= 0

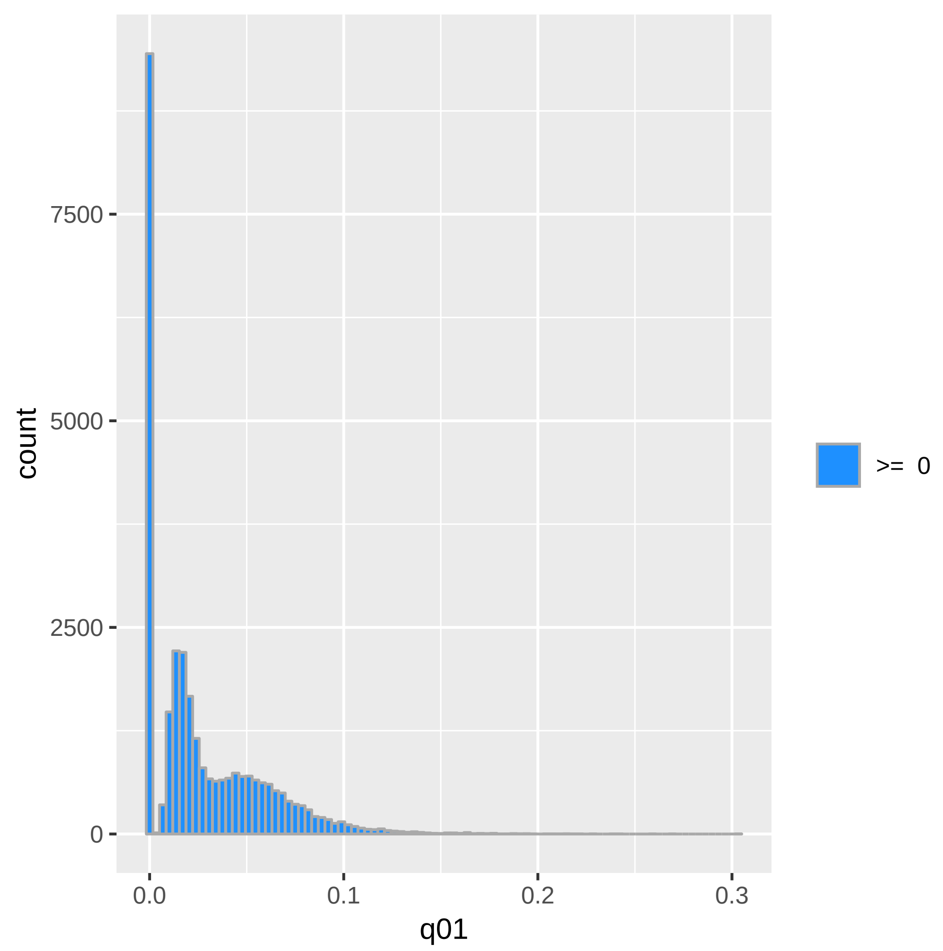
count
7500
5000
2500
0
0.0
0.1
0.2
0.3
q01
>= 0
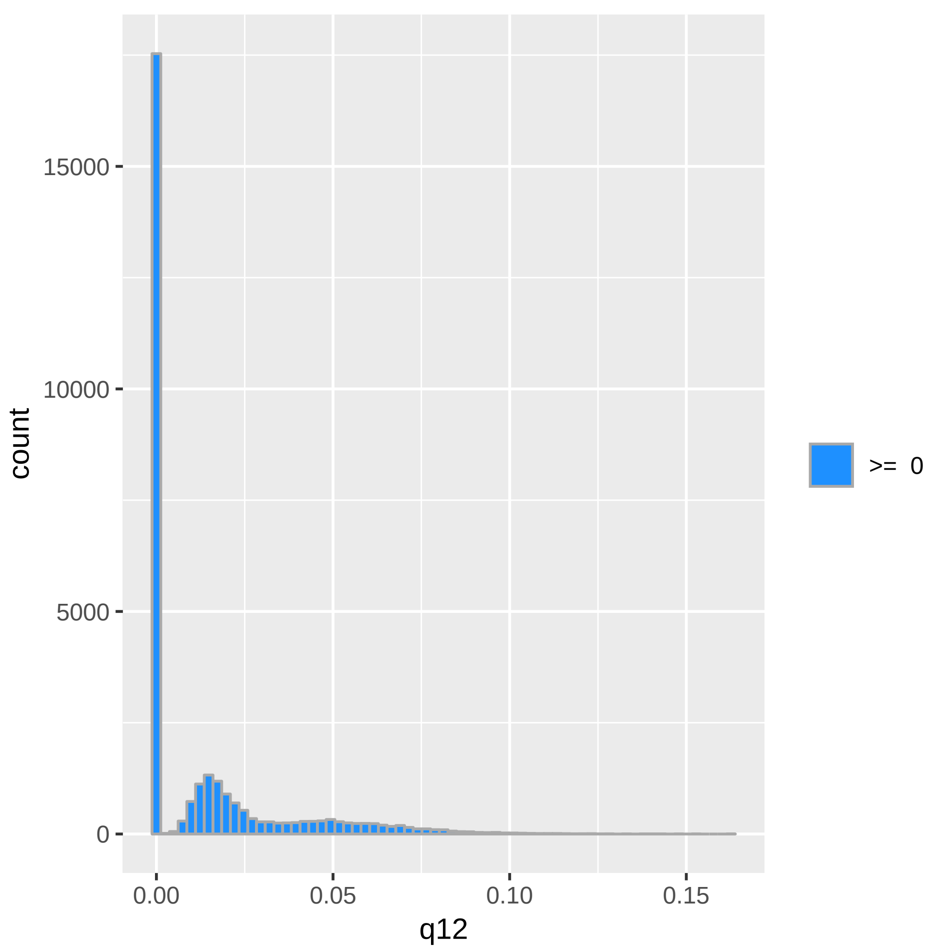
count
15000
10000
5000
0
0.00
0.05
0.10
0.15
q12
>= 0
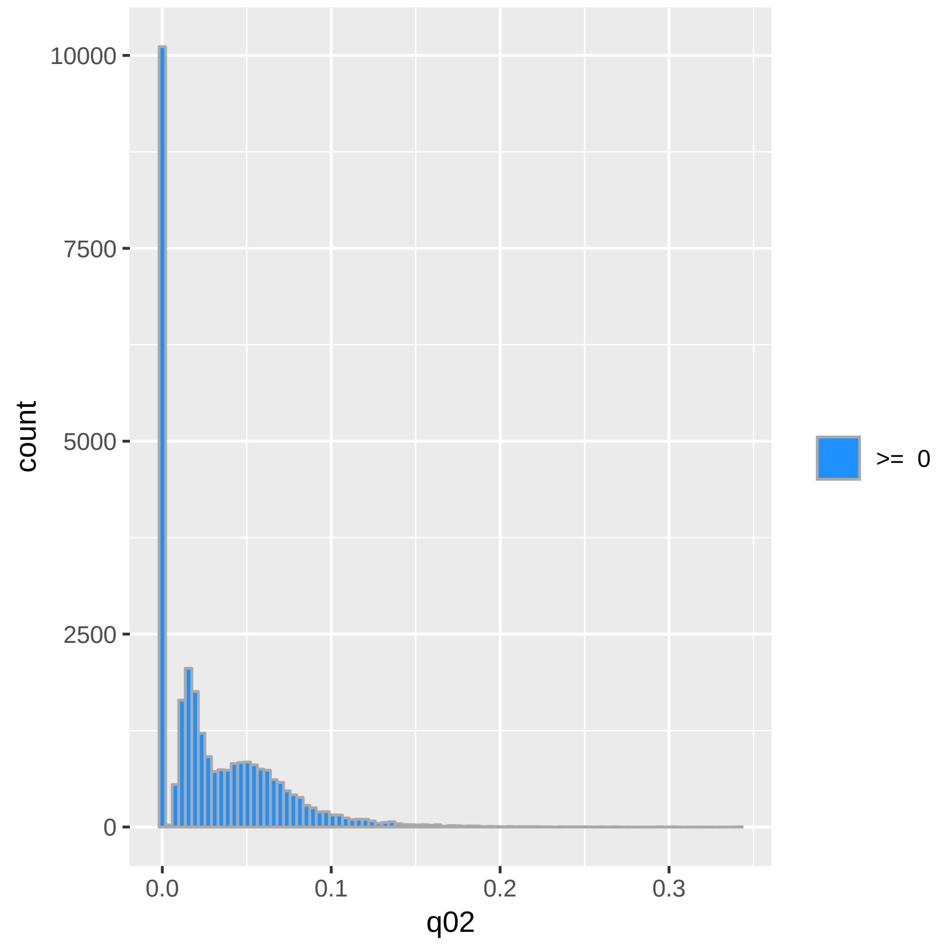
count
10000
7500
5000
2500
0
0.0
0.1
0.2
0.3
q02
>= 0
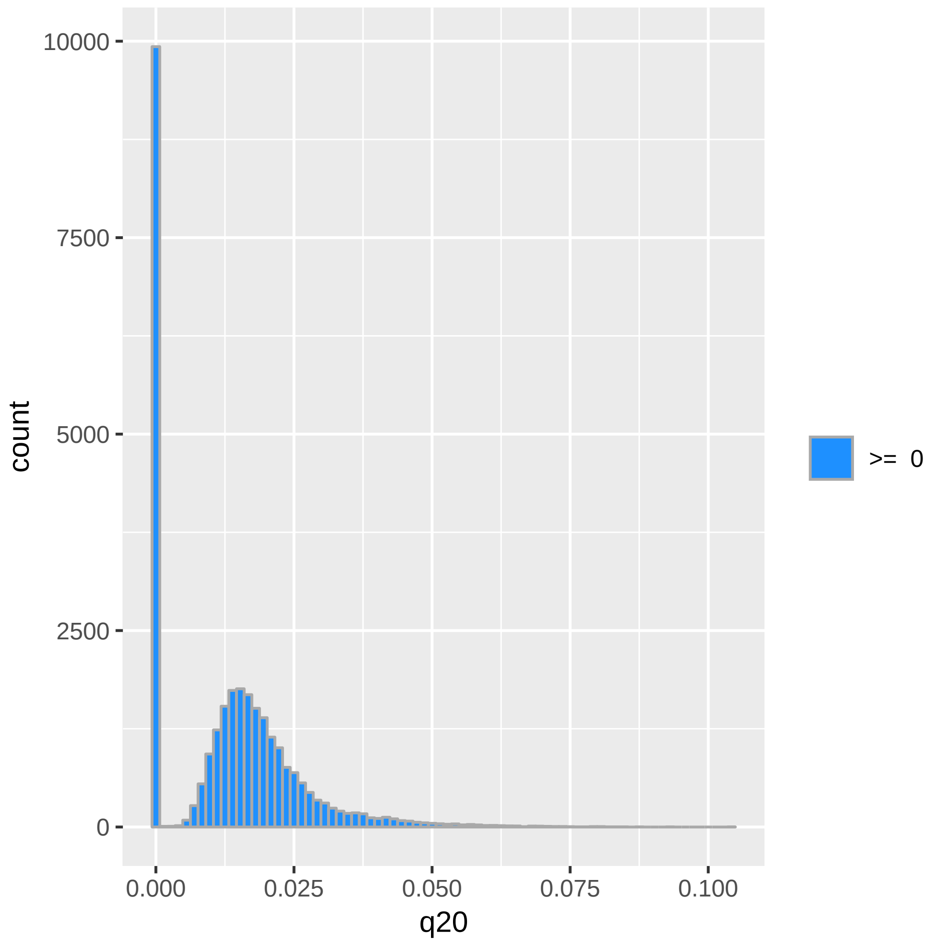
count
10000
7500
5000
2500
0
0.000
0.025
0.050
0.075
0.100
q20
>= 0
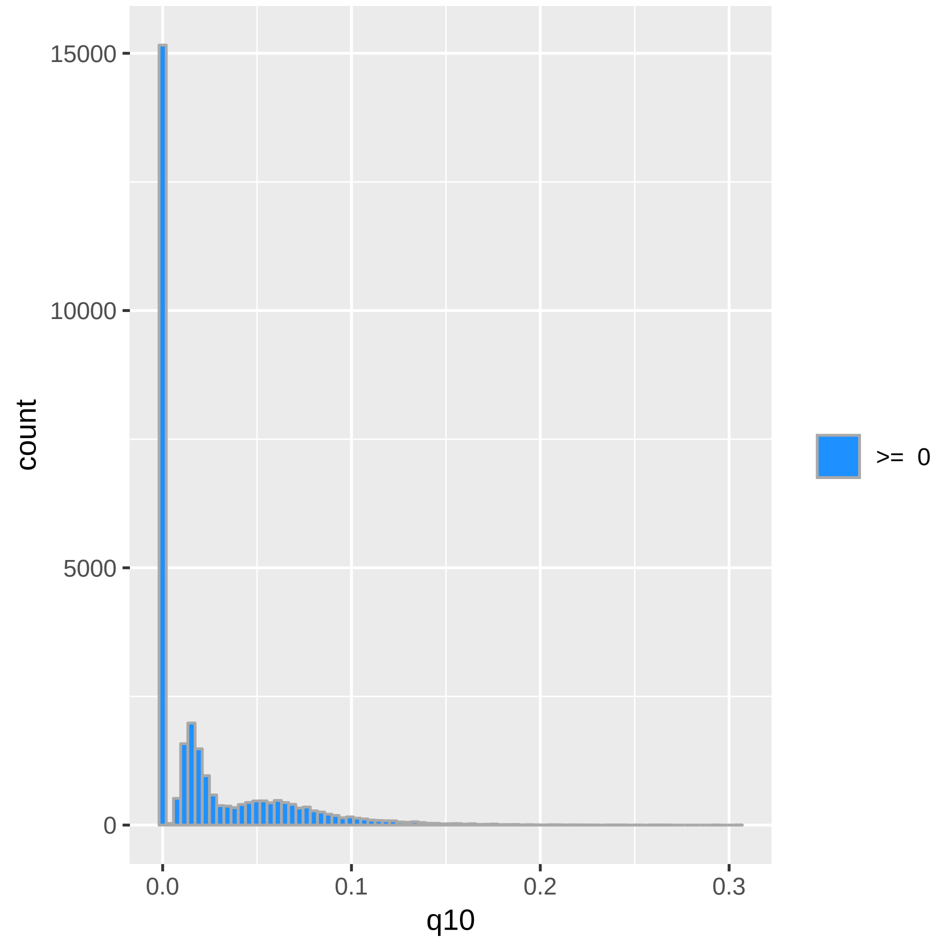
count
15000
10000
5000
0
0.0
0.1
0.2
0.3
q10
>= 0
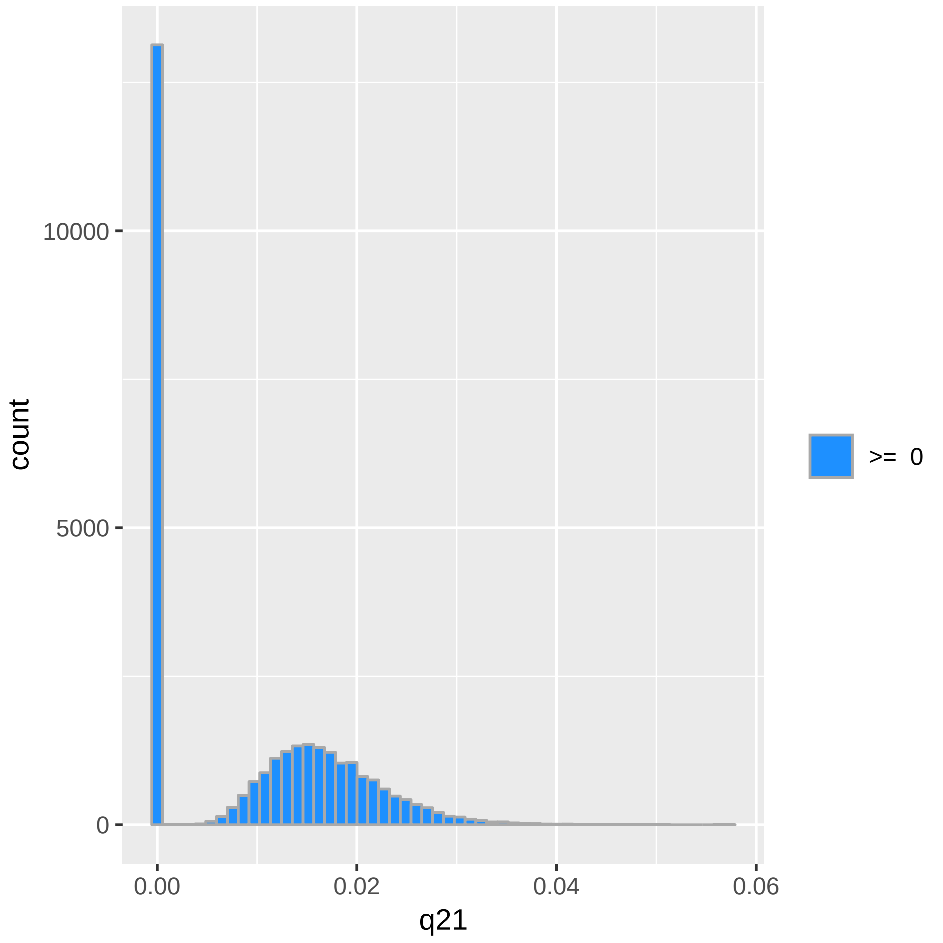
count
10000
5000
0
0.00
0.02
0.04
0.06
q21
>= 0

Appendix S14. Assessment of the MCMC chain from the Reversible-Jump analysis plotted with the function *mcmcPlots* showing the rate coefficients, autocorrelation plot and sliding mean plot.

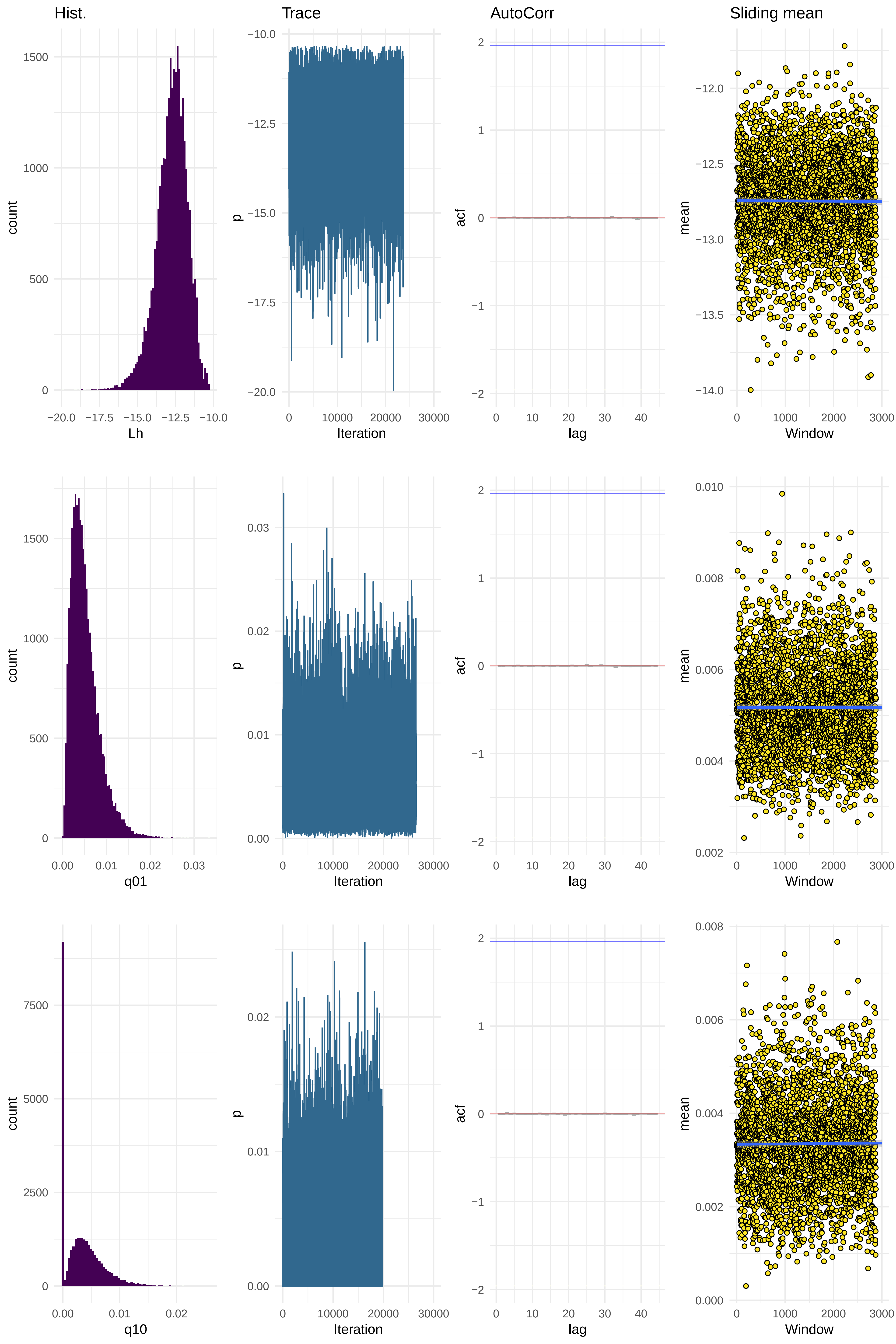
Hist.
Trace
AutoCorr
Sliding mean
count
Lh
q01
q10
p
Iteration
acf
lag
mean
Window

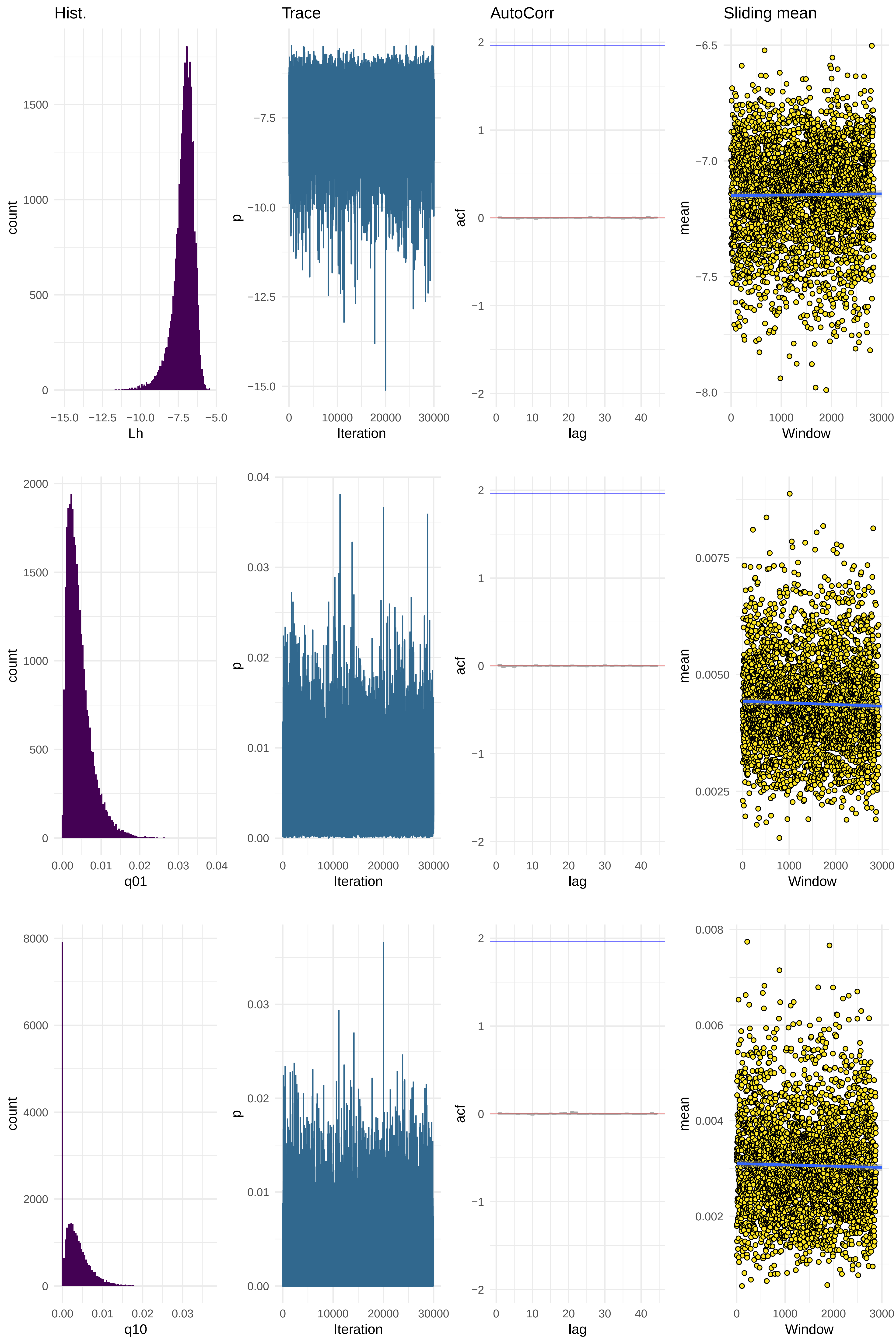
Hist.
Trace
AutoCorr
Sliding mean
count
Lh
p
Iteration
acf
lag
mean
Window
q01
q10

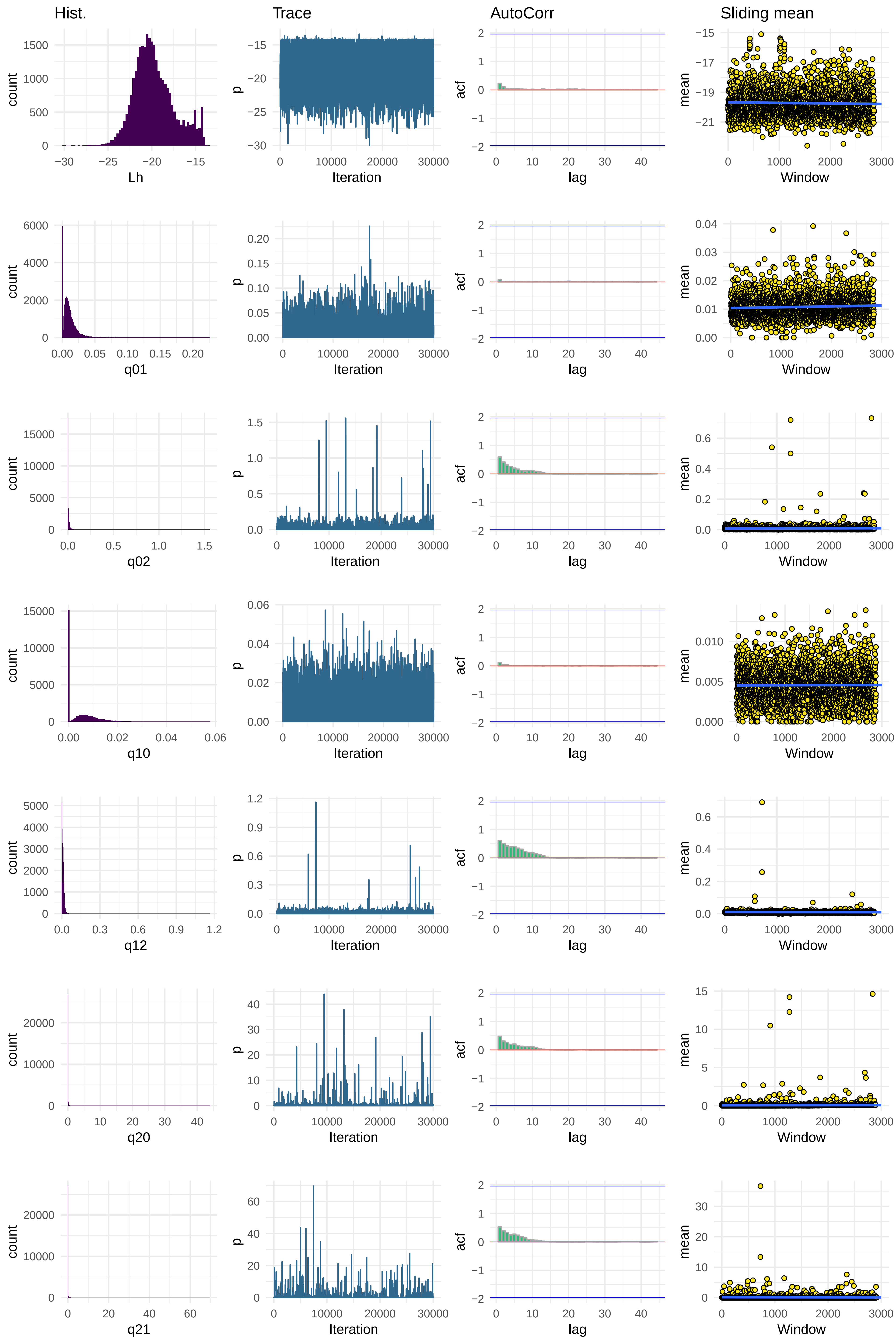

Hist.
Trace
AutoCorr
Sliding mean
count
p
acf
mean
Lh
q01
q02
q10
q12
q20
q21
Iteration
lag
Window

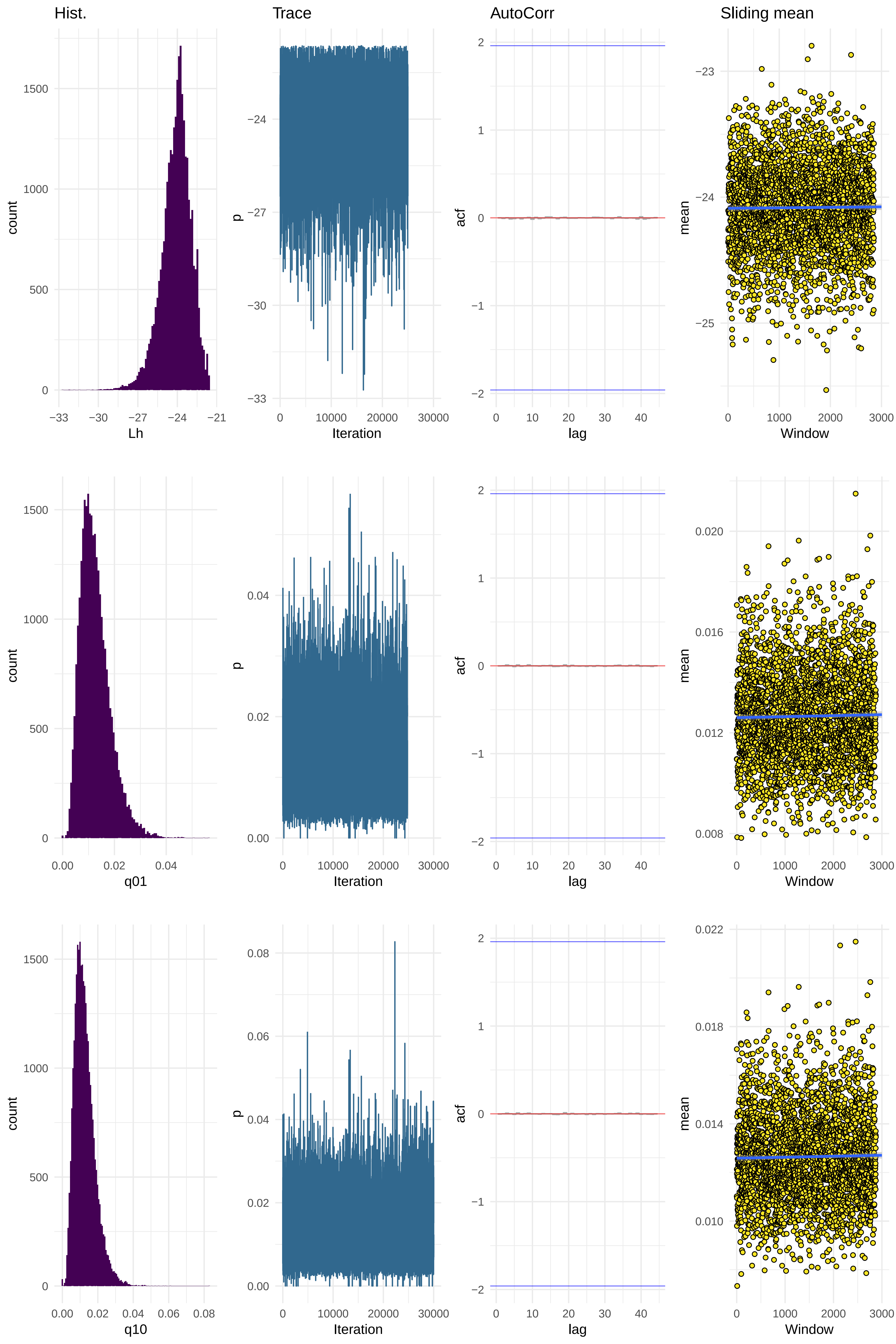

Hist.
Trace
AutoCorr
Sliding mean
count
Lh
p
Iteration
acf
lag
mean
Window
q01
q10

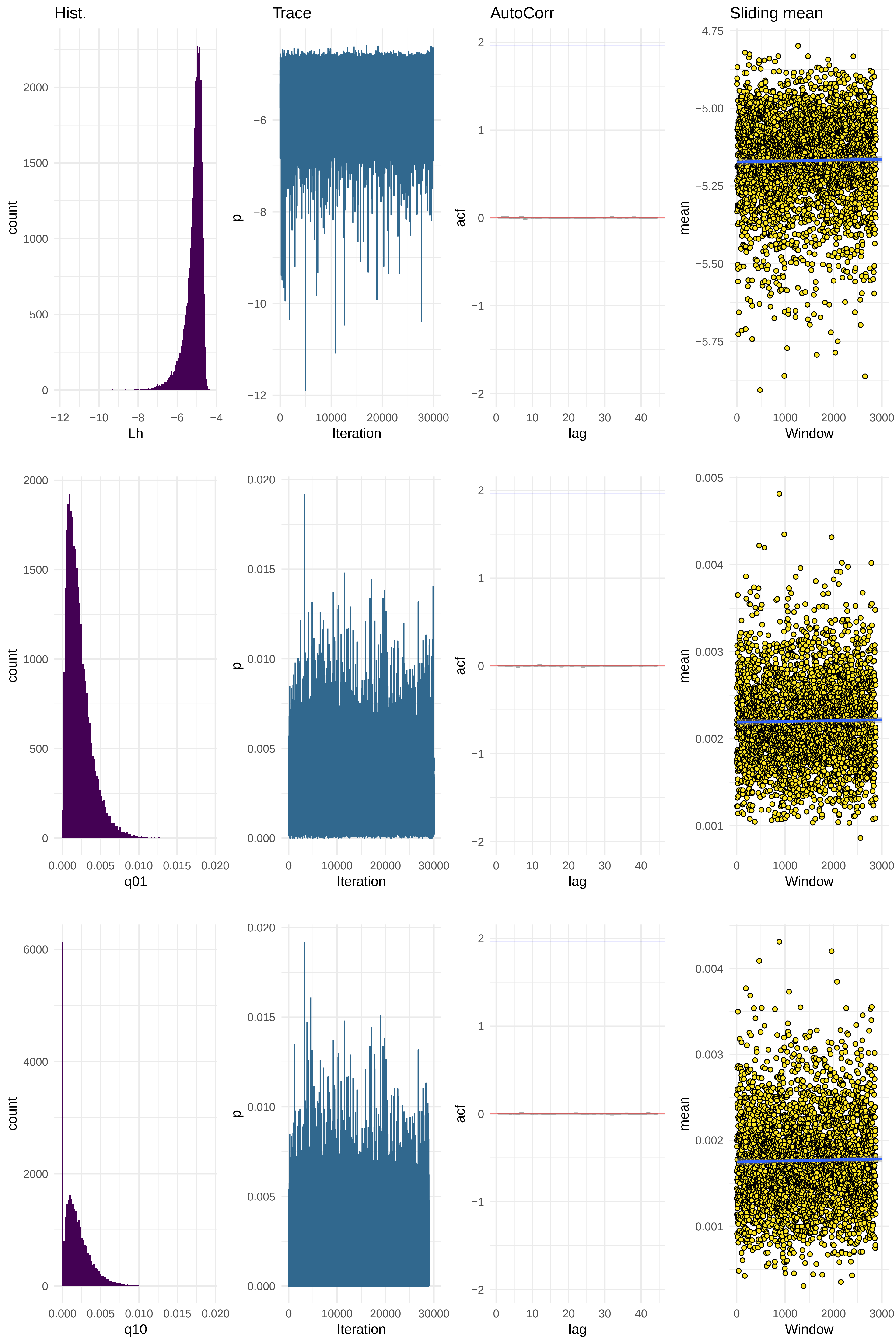
Hist.
Trace
AutoCorr
Sliding mean
count
p
acf
mean
Lh
Iteration
lag
Window
q01
q10

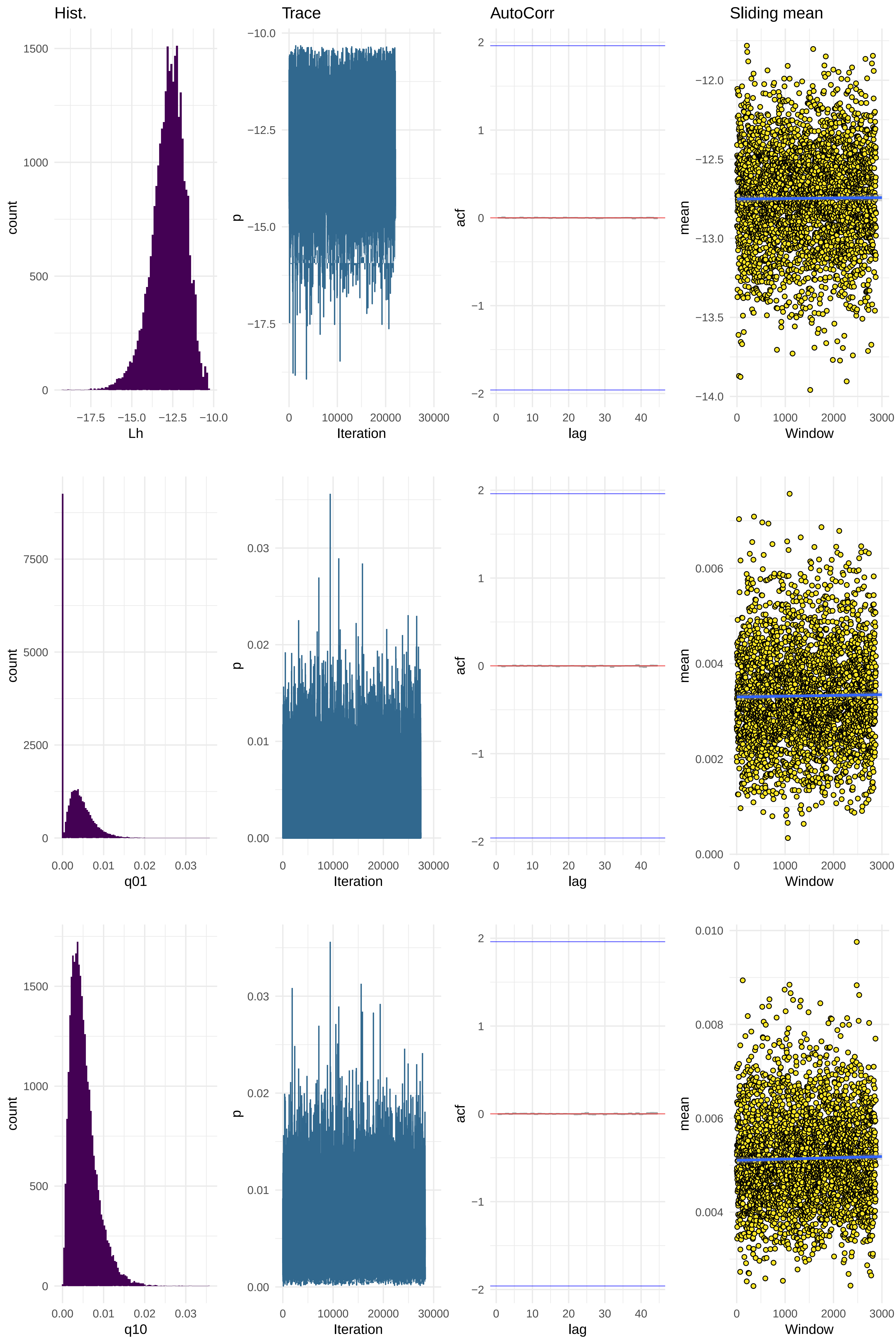

Hist.
Trace
AutoCorr
Sliding mean
count
Lh
p
Iteration
acf
lag
mean
Window
q01
q10

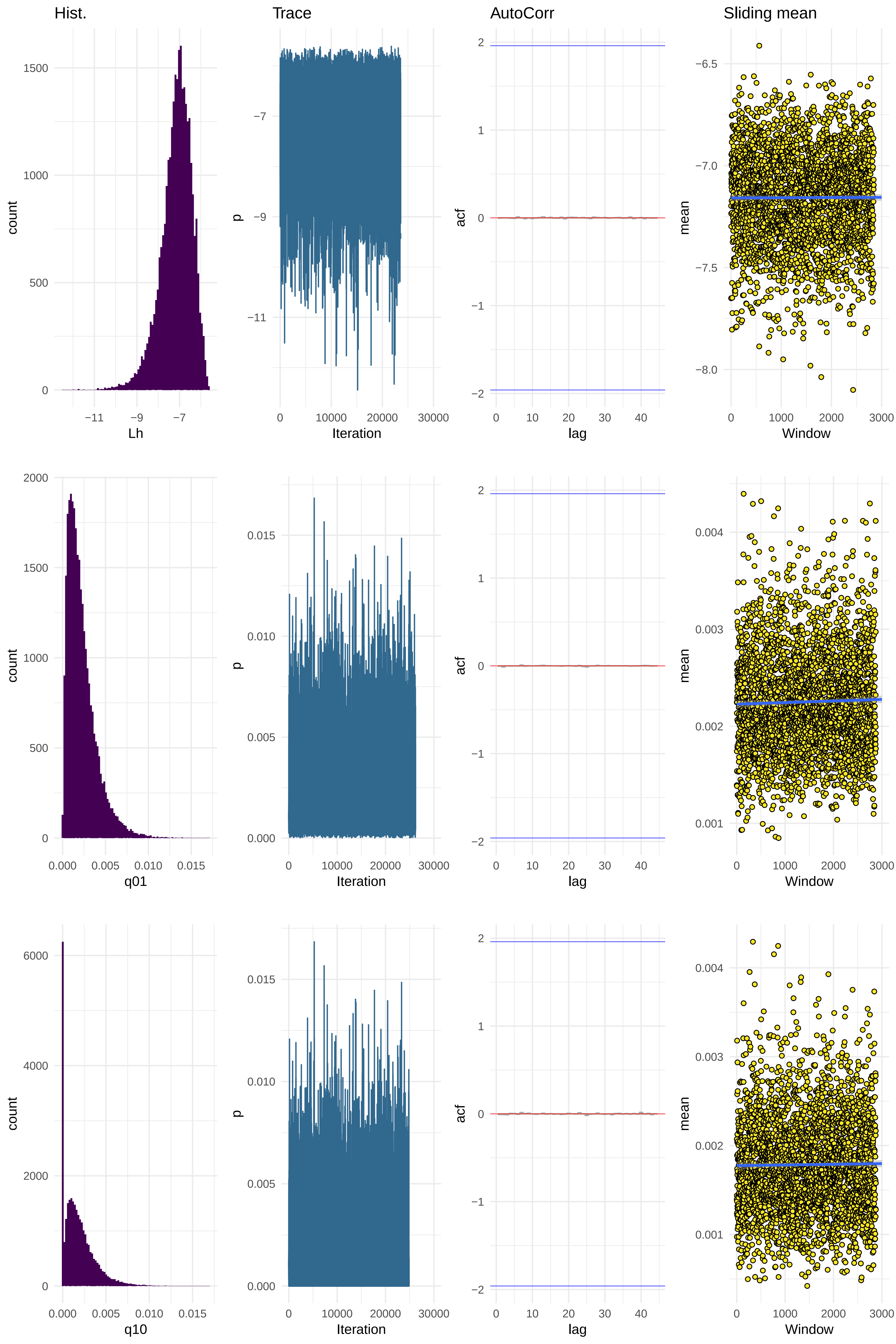
Hist.
Trace
AutoCorr
Sliding mean
count
Lh
p
Iteration
acf
lag
mean
Window
q01
q10

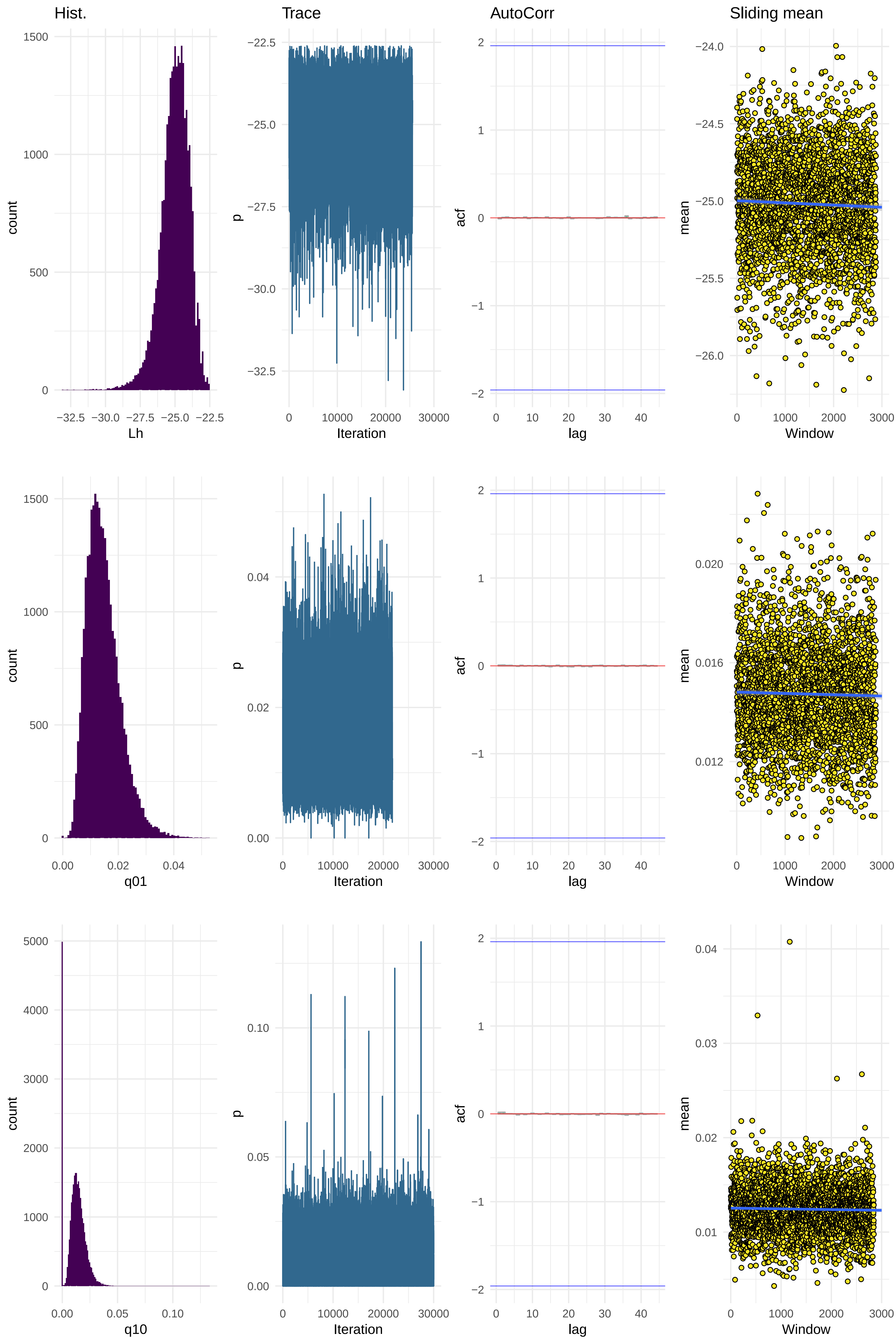
Hist.
Trace
AutoCorr
Sliding mean
count
Lh
p
Iteration
acf
lag
mean
Window
q01
q10

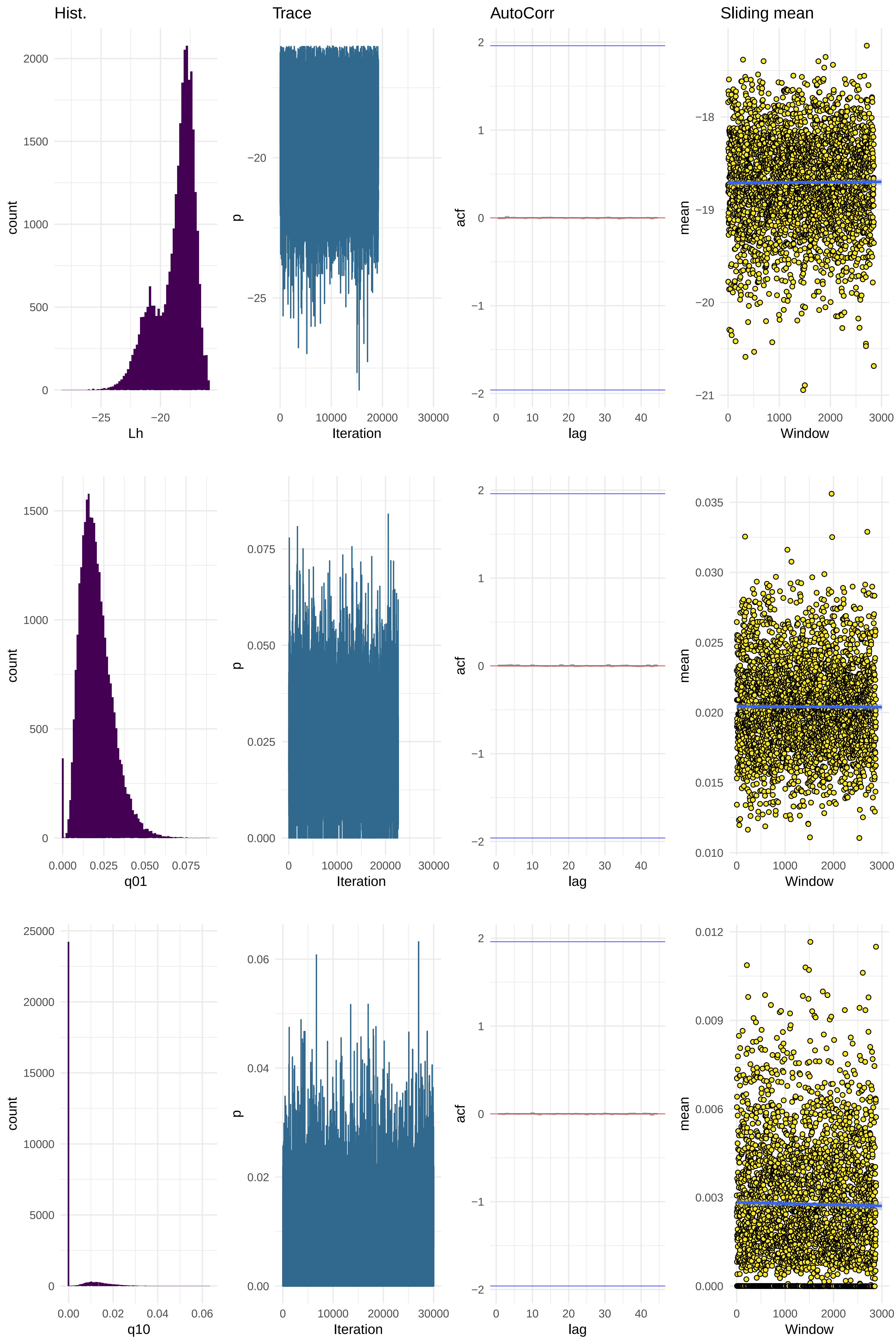

Hist.
Trace
AutoCorr
Sliding mean
count
Lh
p
Iteration
acf
lag
mean
Window
q01
q10

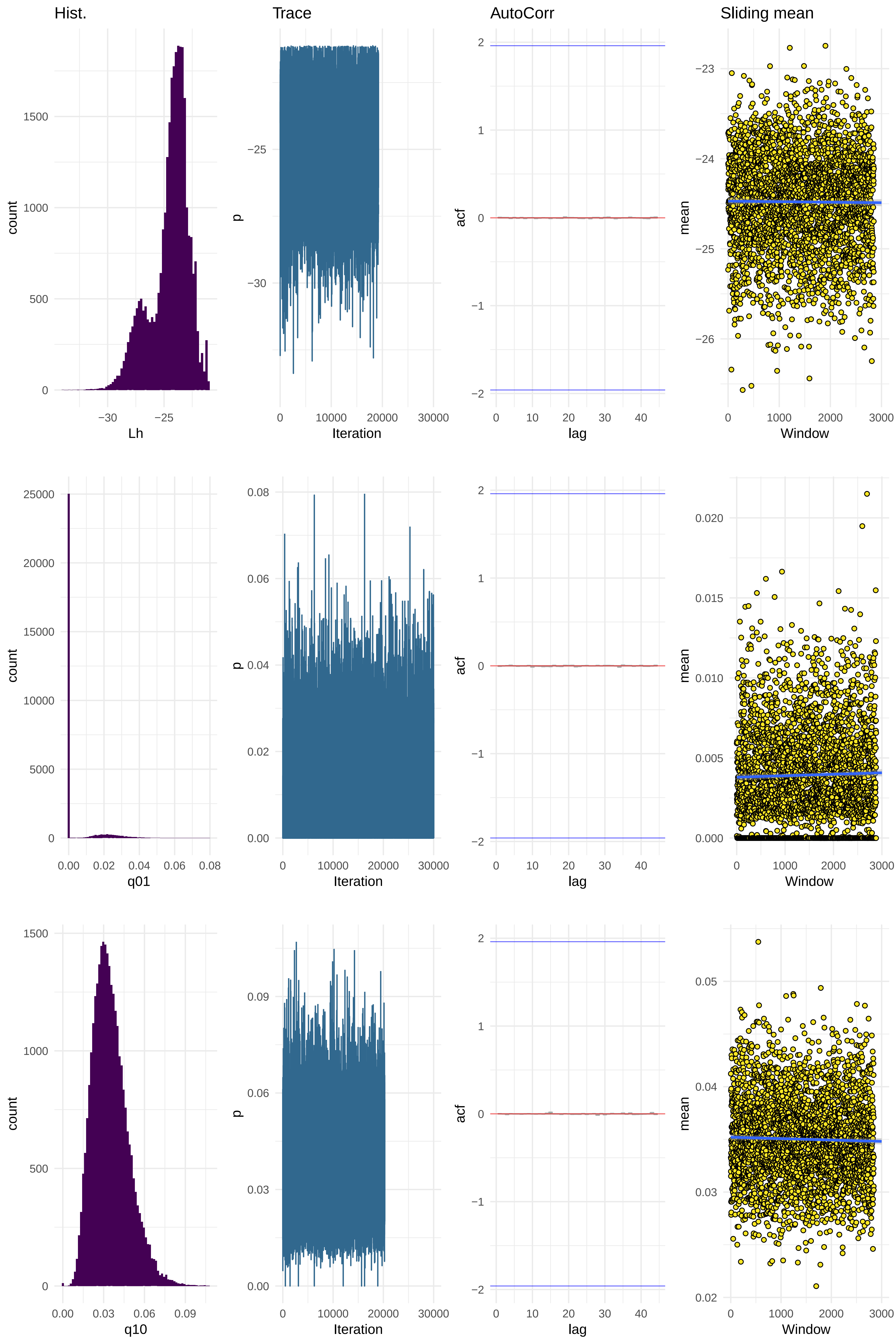
Hist.
Trace
AutoCorr
Sliding mean
count
Lh
p
Iteration
acf
lag
mean
Window
q01
q10

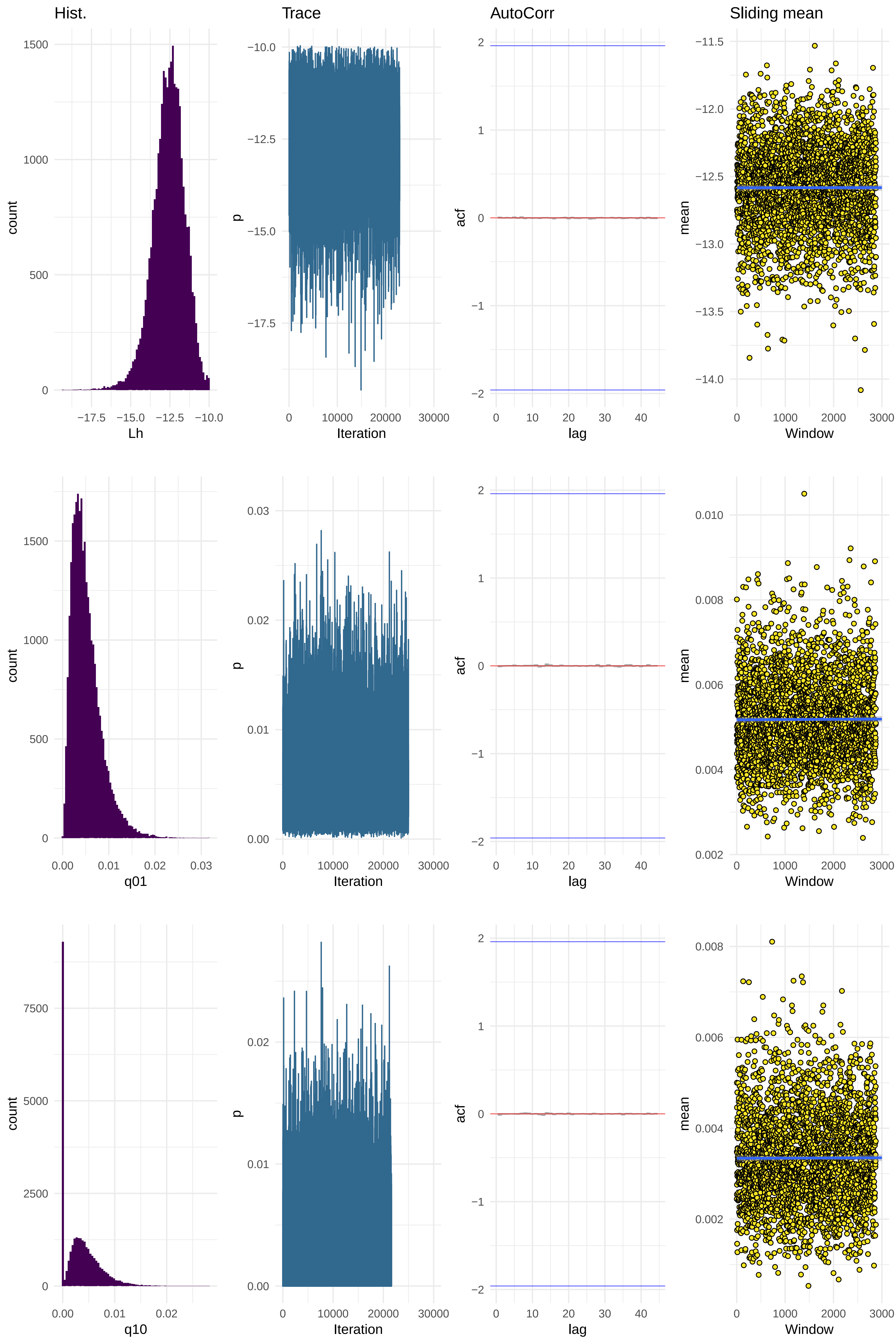

Hist.
Trace
AutoCorr
Sliding mean
count
Lh
p
Iteration
acf
lag
mean
Window
q01
q10

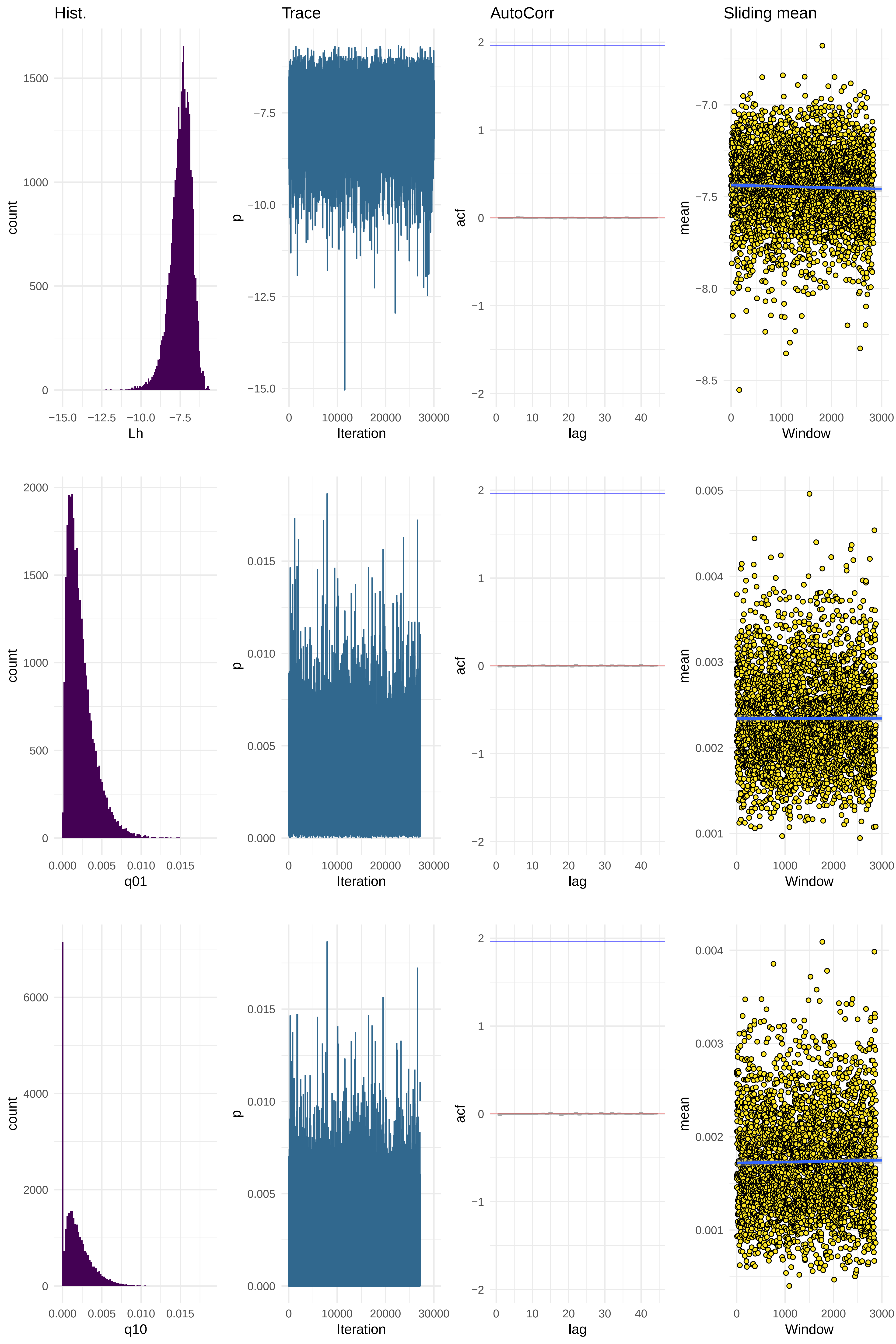

Hist.
Trace
AutoCorr
Sliding mean
count
Lh
p
Iteration
acf
lag
mean
Window
q01
q10

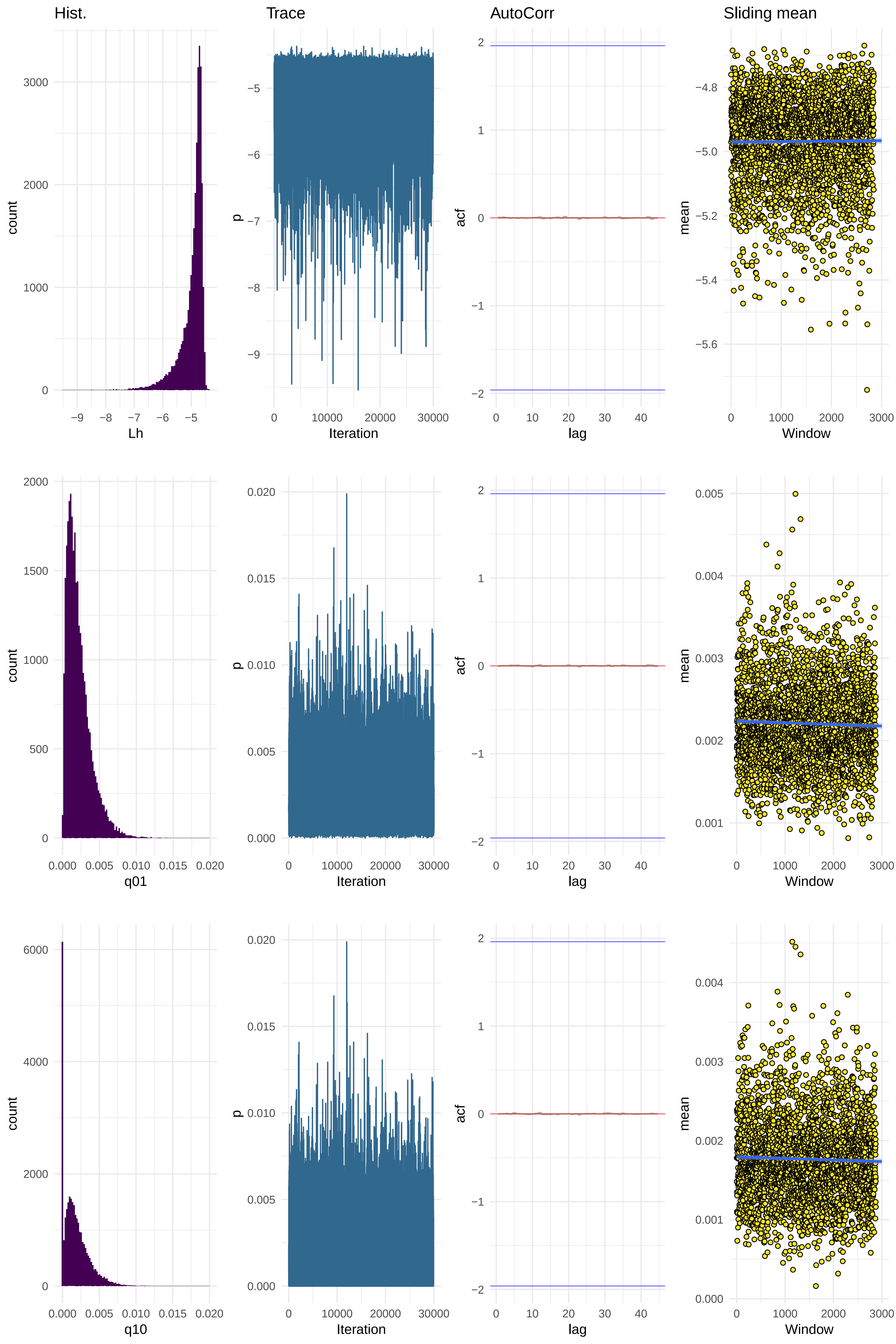
Hist.
Trace
AutoCorr
Sliding mean
count
Lh
p
Iteration
acf
lag
mean
Window
q01
q10

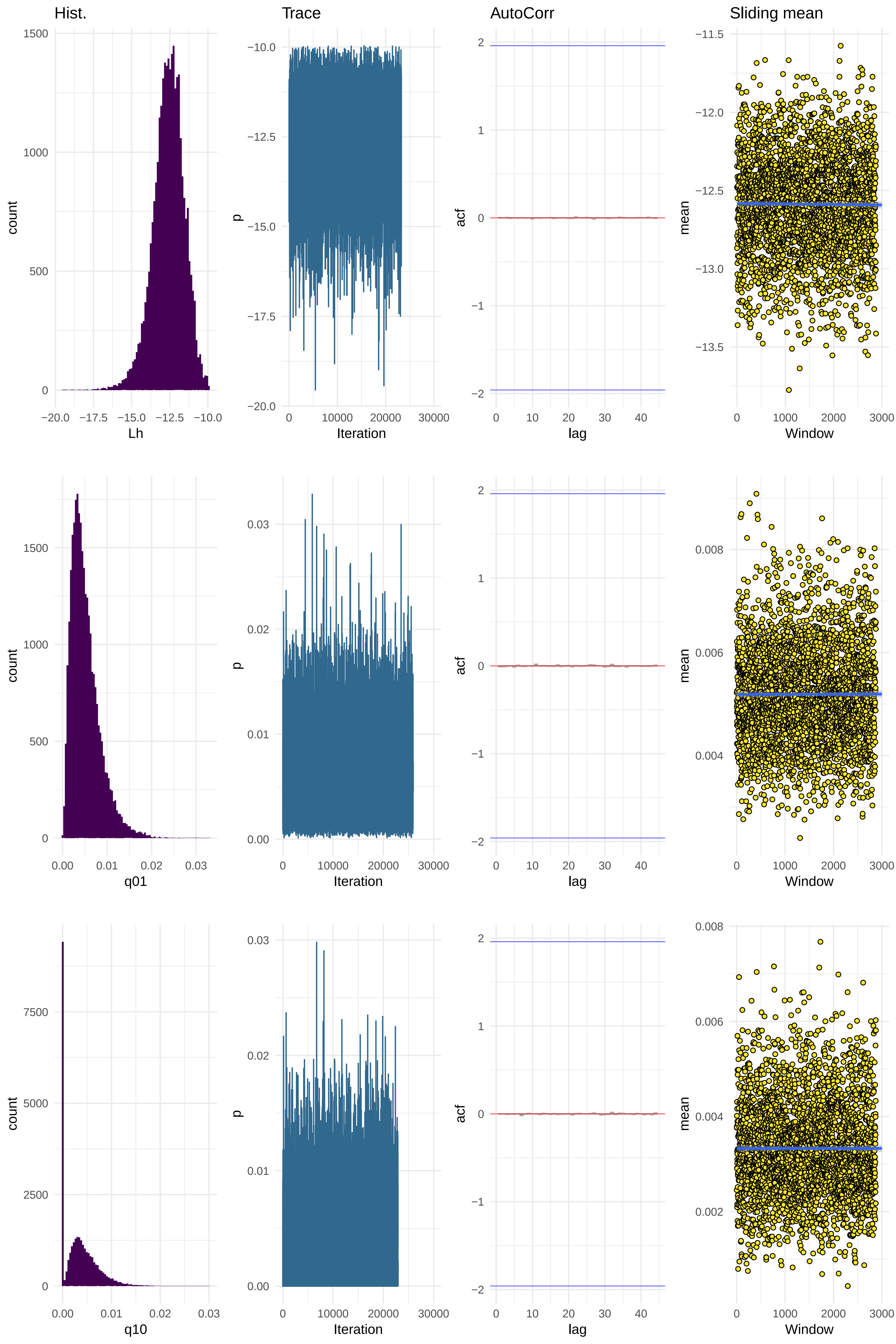
Hist.
Trace
AutoCorr
Sliding mean
count
Lh
p
Iteration
acf
lag
mean
Window
q01
q10

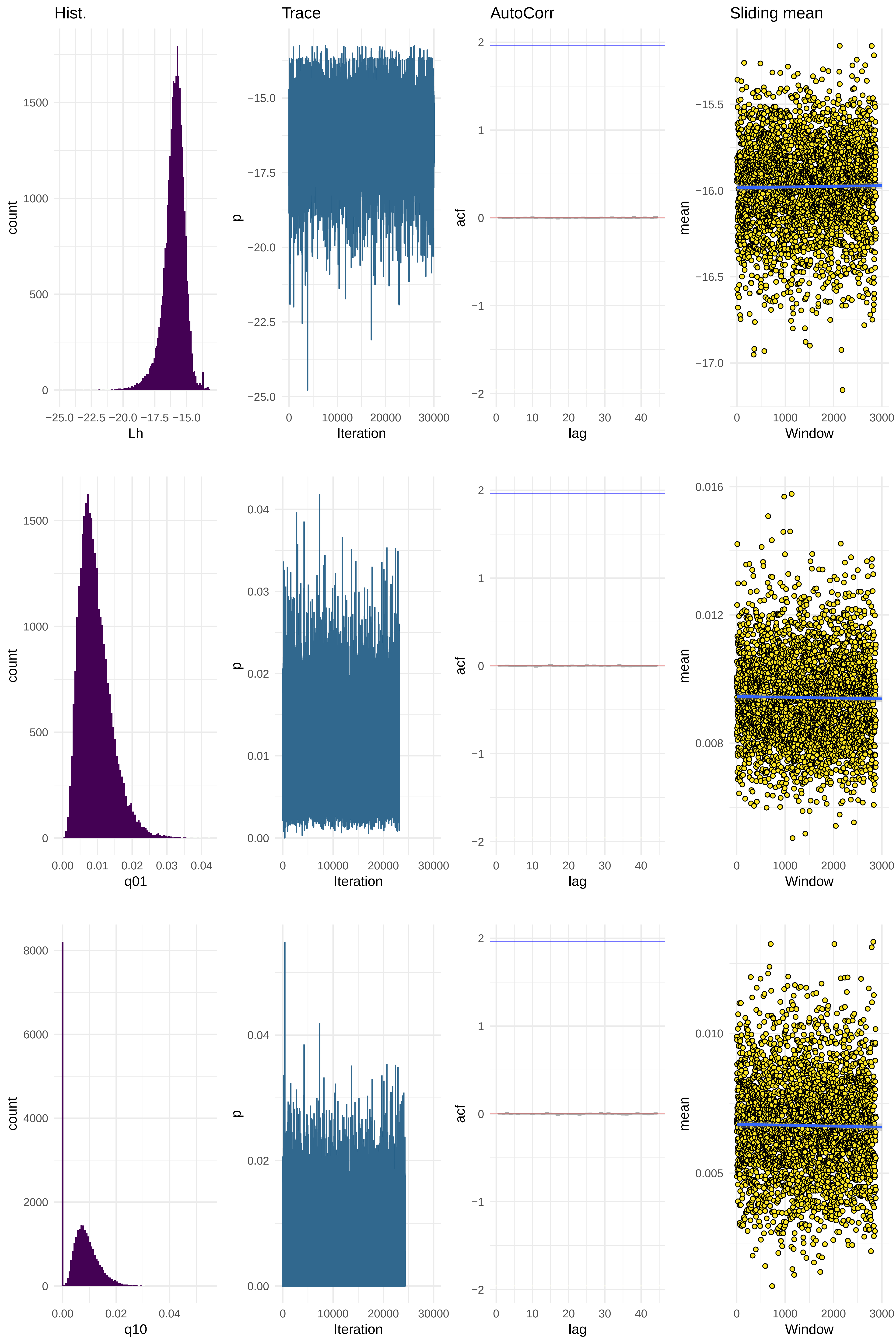

Hist.
Trace
AutoCorr
Sliding mean
count
Lh
q01
q10
p
Iteration
acf
lag
mean
Window

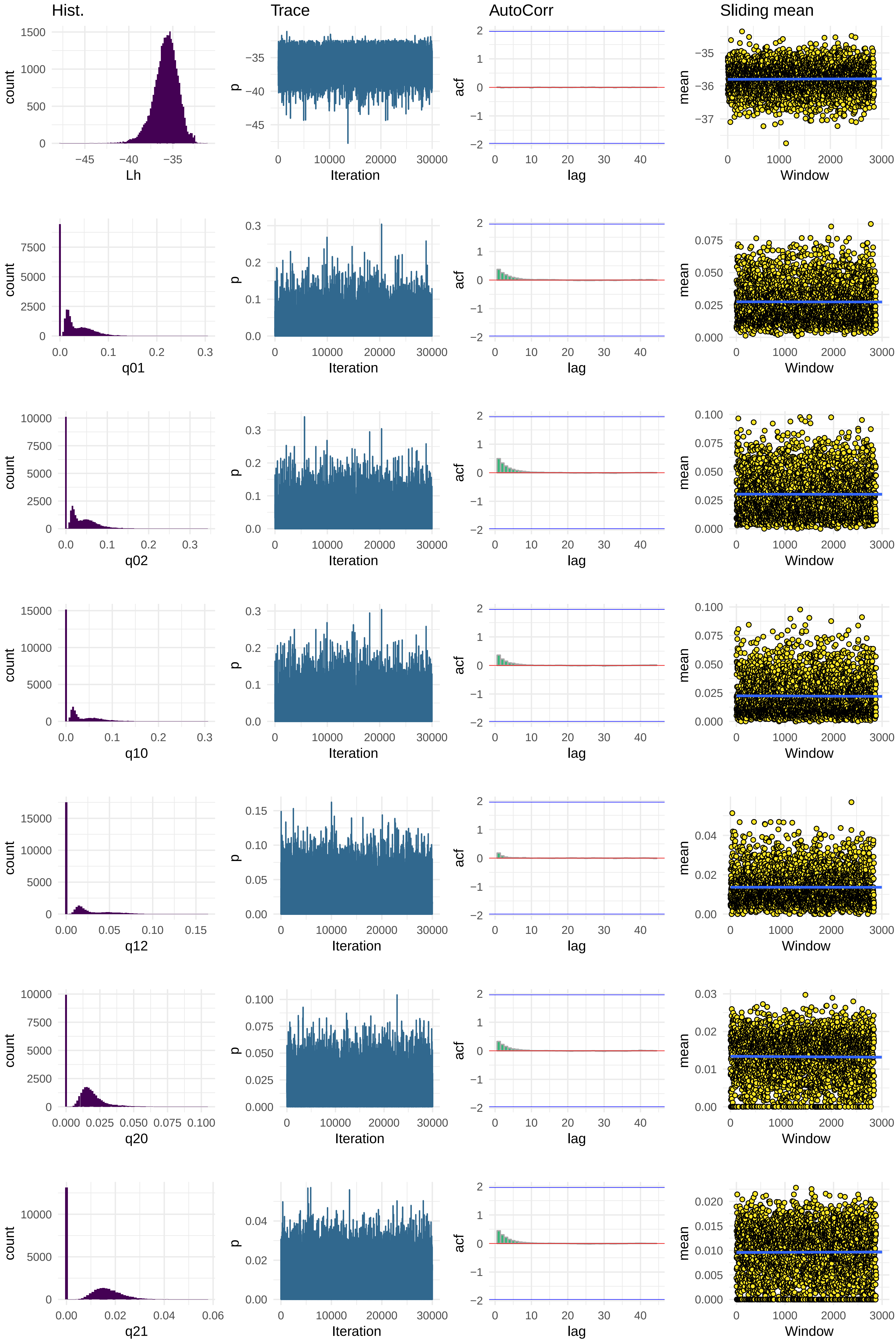
Hist.
Trace
AutoCorr
Sliding mean
count
p
acf
mean
Lh
Iteration
lag
Window
q01
q02
q10
q12
q20
q21

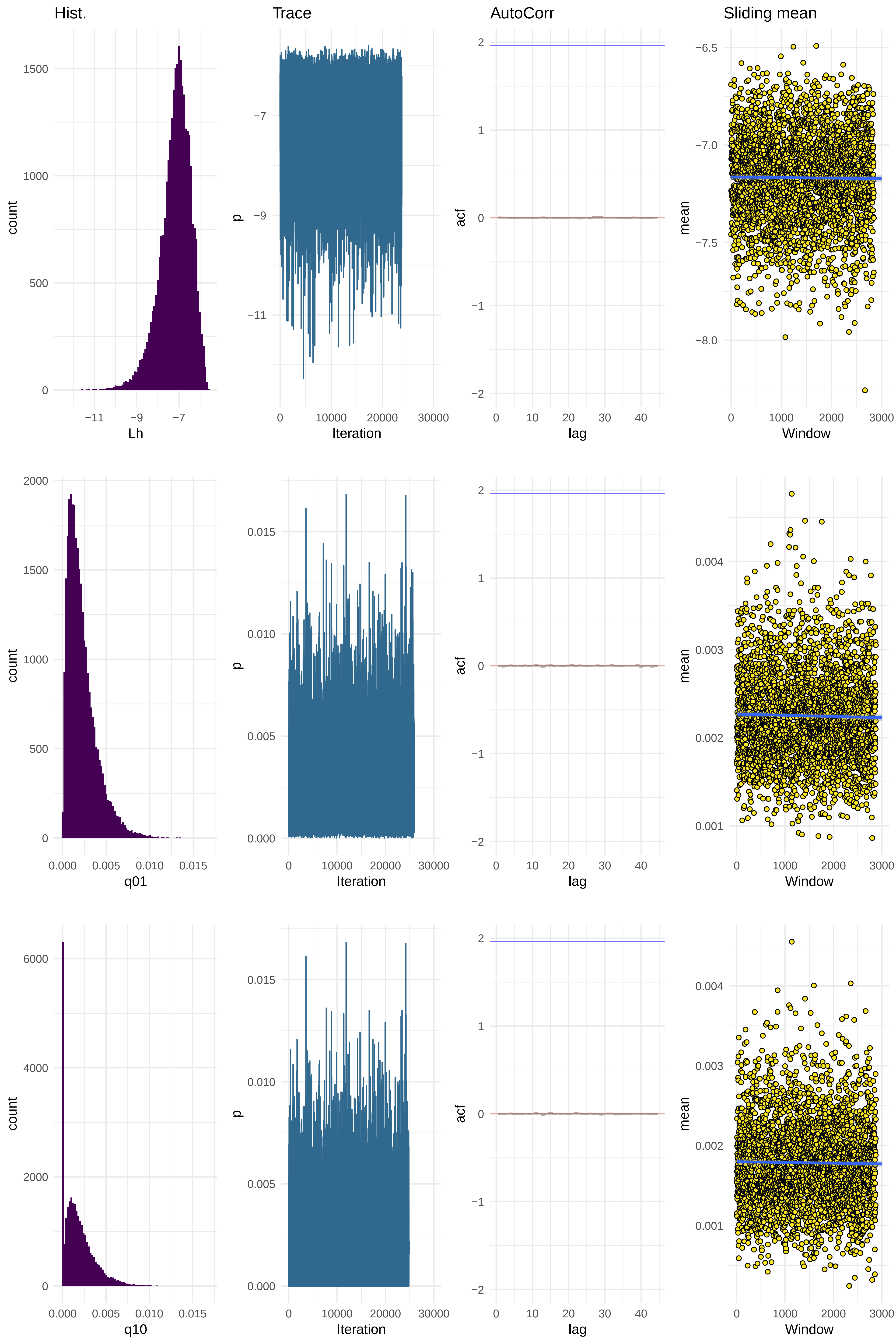
Hist.
Trace
AutoCorr
Sliding mean
count
Lh
p
Iteration
acf
lag
mean
Window
q01
q10

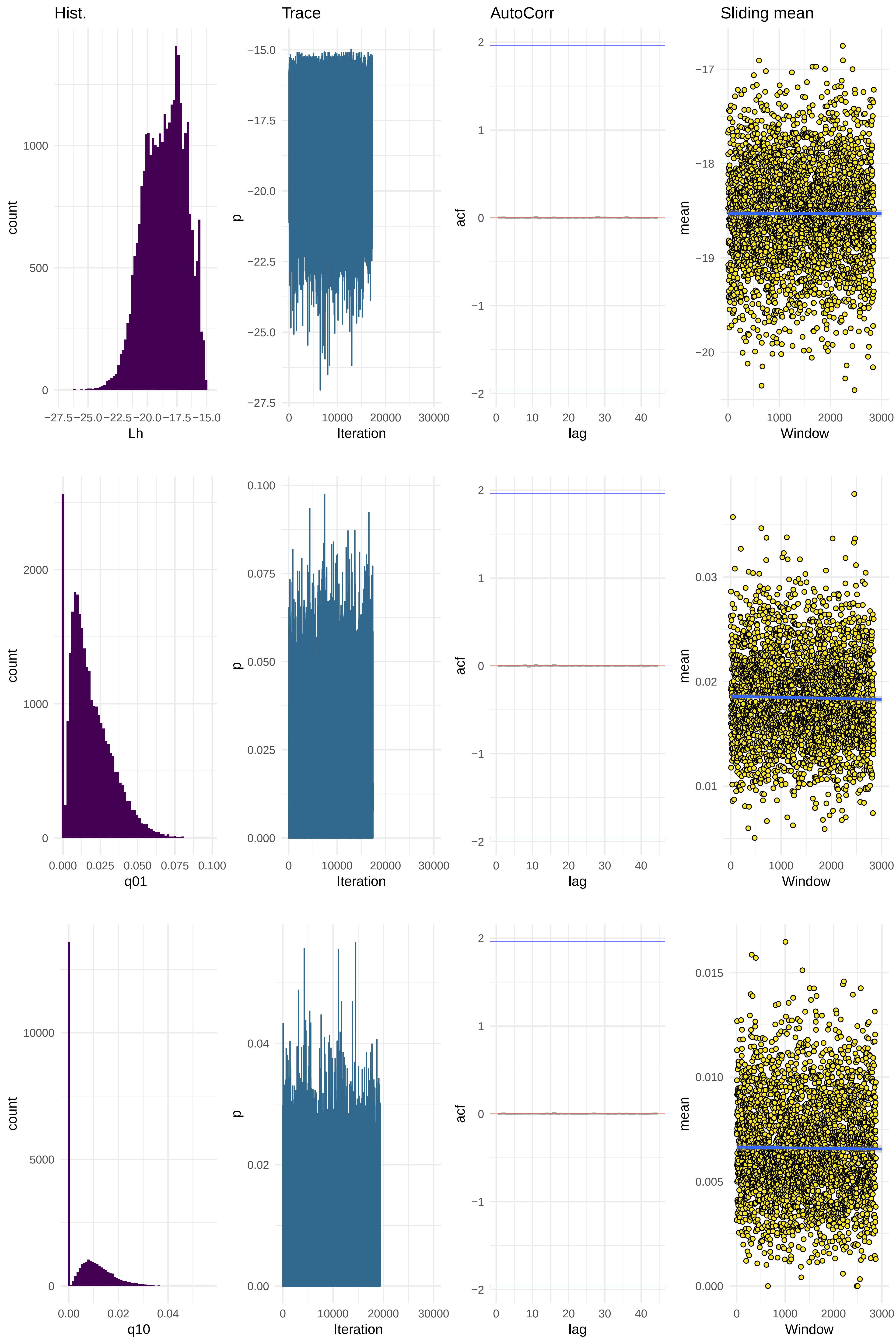
Hist.
Trace
AutoCorr
Sliding mean
count
1000
500
0
−27.5
−25.0
−22.5
−20.0
−17.5
−15.0
Lh
p
−15.0
−17.5
−20.0
−22.5
−25.0
−27.5
0
10000
20000
30000
Iteration
acf
2
1
0
−1
−2
0
10
20
30
40
lag
mean
−17
−18
−19
−20
0
1000
2000
3000
Window
count
2000
1000
0
0.000
0.025
0.050
0.075
0.100
q01
p
0.100
0.075
0.050
0.025
0.000
0
10000
20000
30000
Iteration
acf
2
1
0
−1
−2
0
10
20
30
40
lag
mean
0.03
0.02
0.01
0
1000
2000
3000
Window
count
10000
5000
0
0.00
0.02
0.04
q10
p
0.04
0.02
0.00
0
10000
20000
30000
Iteration
acf
2
1
0
−1
−2
0
10
20
30
40
lag
mean
0.015
0.010
0.005
0.000
0
1000
2000
3000
Window

Appendix S15. The genera previously recognized in the *Lepanthes* clade in the 50% majority-rule consensus tree based on BI analysis of the concatenated dataset. Labels of the genera follow the former generic names proposed[15,30,39]. Plotted branch values for PBP, LPB and PP are given for each well-supported clade of interest. Letters represent genera and numbers clades grouping the genera (see Fig. 3 for the updated proposed classification).

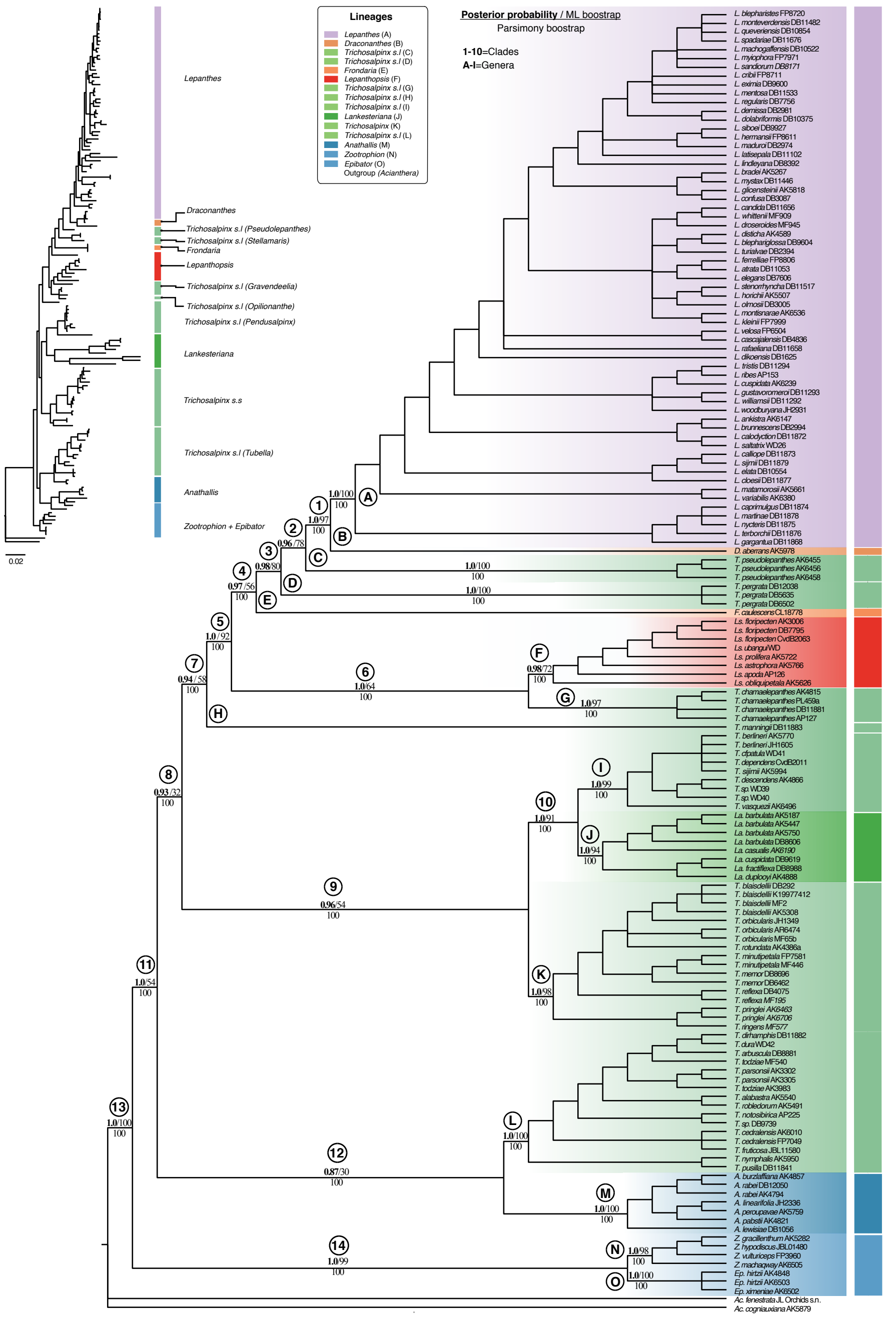

Supplement: Supplementary file 1 — Supporting Information [file 41598_2019_51360_MOESM1_ESM.pdf]
